# Incorporating video telehealth for improving at-home management of chronic health conditions in cats: a focus on chronic mobility problems

**DOI:** 10.3389/fvets.2025.1510006

**Published:** 2025-04-01

**Authors:** Grace Boone, Daniel S. J. Pang, Hao-Yu Shih, Carly M. Moody

**Affiliations:** ^1^Animal Welfare Epidemiology Lab, Department of Animal Science, University of California, Davis, Davis, CA, United States; ^2^Faculty of Veterinary Medicine, University of Calgary, Calgary, AB, Canada; ^3^Faculty of Veterinary Medicine, Université de Montréal, Saint-Hyacinthe, Saint-Hyacinthe, QC, Canada; ^4^Mayo Clinic, Rochester, MN, United States

**Keywords:** veterinary, feline, virtual, accessibility, access to care

## Abstract

**Introduction:**

Feline degenerative joint disease (DJD), commonly referred to as feline arthritis, is one of the most prevalent chronic health conditions in companion cats. DJD results in chronic mobility-related pain and difficulties that require long-term at-home management by the caregiver. Common mitigation strategies include pain control and client education about in-home modifications to make the living environment more comfortable. Cats with chronic mobility problems should receive regular veterinary appointments to monitor the cat’s condition; however, it is well recognized that many caregivers do not bring their cat to see a veterinarian on a routine basis. A possible solution to reducing accessibility barriers, improving compliance, and increasing access to pet education is veterinary video telehealth.

**Methods:**

The current study used video visits to assess the impact of telehealth on caregiver education and home care of cats living with chronic mobility difficulties. US and Canadian caregivers of companion cats with chronic mobility difficulties or arthritis (*N* = 106) filled out a recruitment survey and then two study questionnaires approximately four months apart. The study questionnaires included questions regarding their cat’s mobility, their attitudes toward using video telehealth, and preference for video telehealth or in-person visits for various veterinary appointment types. Participants were randomly allocated to a treatment (*n* = 63; 6 video visits every 3 weeks over approximately 4 months) or a control (*n* = 43; no video visits) group.

**Results and discussion:**

Overall, the results suggest caregivers were interested in and preferred video telehealth appointments to assist with managing their cat’s chronic mobility challenges. In addition, undergoing the synchronous video telehealth appointments increased participant knowledge of their cat’s mobility challenges and perceived helpfulness of their at-home management strategies. This suggests that from the caregiver’s perspective, the video telehealth appointments were beneficial for both themselves and their cat. There was also evidence that caregivers whose cats were more mobility impaired (higher Feline Musculoskeletal Pain Index – short form score) were associated with increased interest in using veterinary telehealth for at-home management of their cat. Further research should assess the impact of common environmental modifications implemented to improve cat comfort, on health and behavior outcomes for cats living with chronic mobility problems.

## 1 Introduction

There are an estimated 37–40 million households with at least one companion cat in the United States (1, 2). A leading chronic health condition for companion cats is degenerative joint disease (DJD), commonly referred to as feline arthritis or osteoarthritis (OA), although OA is a form of DJD (3–5). DJD is an umbrella term encompassing various types of joint-related destruction, including cartilage, bone, ligaments or the joint capsule (4). Estimates of the prevalence of DJD in cats vary widely, due to variations in study design and populations studied. However, estimates suggest 22–92% of cats have at least one joint affected by DJD (4, 5). Older cats are more likely to be affected, with one study showing an estimated 13.6% increase in expected DJD scores with each one-year increase in a cat’s age (5). Thus, older cats may need more frequent veterinary visits for ongoing management (6). Common caregiver-reported symptoms of DJD include reductions in activity and mobility, especially difficulties with jumping and navigating stairs (7–9). In part, this is likely because DJD is often painful (4, 8, 10, 11). In addition, cat mobility problems may lead to difficulties in accessing important resources around the home such as sleeping and resting spots, litter boxes, and food and water (6, 9). Inability to access these resources is a health and welfare concern. Cats with chronic mobility difficulties and/or suspected DJD should be examined by a veterinarian for diagnosis and development of a management plan. This should be followed by regular visits to monitor the cat’s condition and to increase the success of long-term management. Common mitigation strategies for chronic mobility problems and DJD include pain control medications and/or joint supplements, as well as client education about in-home modifications to make the living environment more comfortable (3, 12). Environmental modifications are important because they help cats access necessary resources in the home and are meant to improve cat comfort. These may include, among other things, elevated food and water bowls so the cat does not have to bend their legs or extend their neck to eat or drink, stairs or ramps to help the cat access elevated areas without jumping, and extra-large, shallow litter pans to facilitate easier elimination (12). It is important to note that no research has assessed the efficacy of these types of interventions for cats living with chronic mobility problems.

Although it is essential that cats with chronic mobility difficulties and/or suspected DJD receive regular veterinary visits to monitor their condition, current literature suggests many caretakers do not bring their cat to see a veterinarian on a routine basis (13, 14). Research on barriers to accessing care has identified cat-related barriers which include difficulties with getting a cat into a carrier, stress associated with travel, and stress associated with the veterinary clinic experience (13–17). Caregiver-related barriers often involve socioeconomic factors such as access to transportation and geographic location (18). In addition to these barriers, some caregivers may attribute chronic mobility-related changes to natural aging and leave the condition sub-optimally managed (19) or not managed at all. This is a welfare concern given that unrecognized or underestimated pain may lead to suffering and reduced welfare.

A potential strategy for improving caregiver access to veterinary care and education is the use of video telehealth technologies. Video telehealth is defined as using video technology to deliver health and behavior care, education, and/or information remotely (adapted from the (20)). A benefit of using video telehealth is the ability to assess the cat’s mobility and behavior in the home, where cats are more comfortable, more likely to show normal behavior, and less likely to hide signs of pain (19, 21, 22). In addition, the veterinary team is able to observe the cat’s home environment directly. This is particularly important for cats living with chronic mobility difficulties, as environmental modifications are a large and important component of effective management. Telehealth encompasses a wide range of veterinary applications. The present study is focused on tele-advice via synchronous video calls which can occur in the comfort of the cat’s home. Tele-advice does not involve medical evaluations, diagnostics, or treatments (which require an established veterinarian-client-patient-relationship or VCPR, and would constitute telemedicine), and thus may be performed by either a veterinarian or knowledgeable non-veterinarian staff member (20). Video technology may also reduce follow-up difficulties and increase access to education for those who do not bring their cat to a veterinarian on a regular basis. Research on US cat caregiver attitudes toward using telehealth indicates a high level of interest in video appointments, and that use would increase their accessibility to veterinary care and reduce their own and their cat’s appointment-related stress (16, 17). In addition, this research suggests most US caregivers are willing to pay for these services and prefer this technology over in-clinic appointments for following up on their cat’s health and receiving support with home management of chronic health conditions.

The aim of the current study was to assess how video tele-advice visits may help improve caregiver access to education, home care, and cat health [i.e., body condition score (BCS)] for their cats living with chronic mobility challenges or DJD. Since DJD is a technical term, we opted to use the term arthritis when communicating with participant cat caregivers. We predicted that use of video visits for caregivers of cats with arthritis and chronic mobility challenges would improve the following: (1) confidence in knowledge of their cat’s chronic mobility challenges or arthritis, (2) perceived helpfulness of at-home management strategies implemented to support their cat, (3) cat mobility in the home and improved BCS, (4) interest in using video telehealth appointments to support their cat’s chronic mobility challenges or arthritis, and (5) willingness to pay for these visits.

## 2 Materials and methods

This research was reviewed and deemed exempt (i.e., does not require registration) by the UC Davis Institutional Animal Care and Use Committee (#23338) and the UC Davis Institutional Review Board (#1998883).

**Figure 1:**
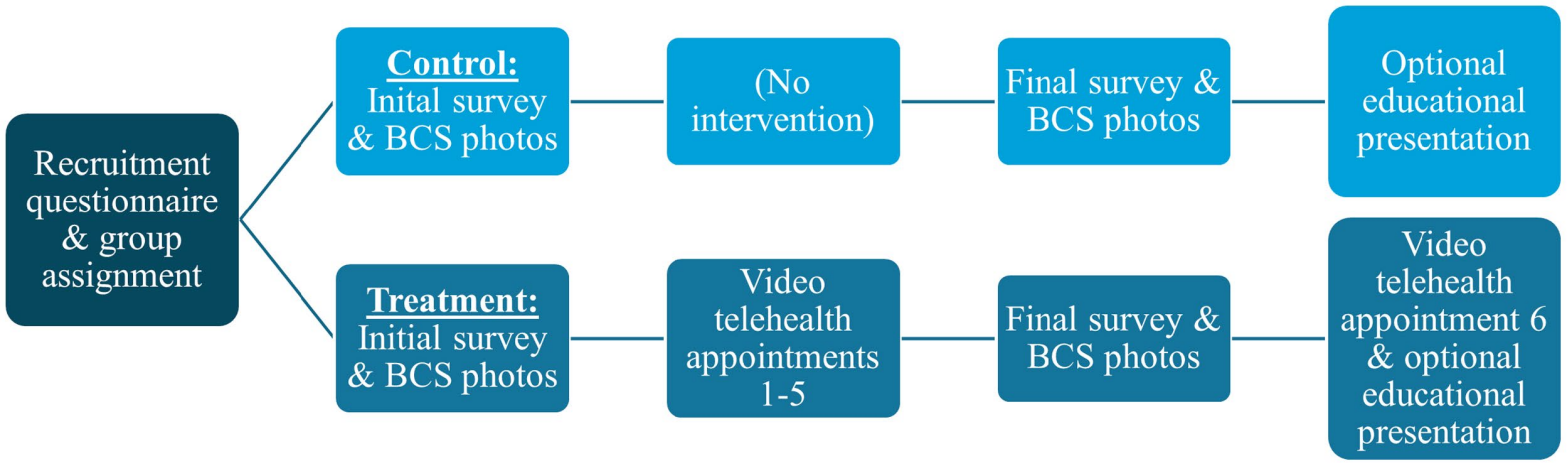
Flowchart showing the sequential process of study participation for cat caregivers in the treatment (*n* = 63) and control (*n* = 43) groups.

This research used a between-subject design and two groups: treatment and control. Treatment group participants underwent 6 video tele-advice appointments, whereas control group participants did not. Participation in the research required the following: (1) recruitment questionnaire, (2) pre-study initial questionnaire, (3) pictures of their cat for pre-study body condition scoring (BCS), (4) six video tele-advice visits over 4-months (treatment group only), (5) final questionnaire (4-months after initial questionnaire), (6) pictures of their cat for post-study BCS, and (7) optional educational presentation on home management of cats with chronic mobility conditions (Figure 1). The three questionnaires (recruitment, initial, final) were created using Qualtrics survey software (Qualtrics Software Company, Provo, Utah, USA) and were available online. A consent form outlined study participation and stated that participation was voluntary. Participants were free to withdraw at any time and only those consenting to the study procedures could access the recruitment questionnaire for study sign-up.

### 2.1 Recruitment and participant screening

To participate in the research, caregivers needed to be at least 18 years of age, currently residing in the US or Canada (including US/Canadian territories), have access to a smart device with video capabilities and internet access, and be the current primary caretaker of at least one companion cat living with chronic mobility challenges or arthritis who spends at least 50% of their time indoors.

The online recruitment questionnaire [17 questions; Supplementary material 1 (S.1.1)] included caregiver and cat-related questions. Caregiver questions included the respondents’ age, location (country and state or territory; rural, suburban or urban), time zone (for appointment scheduling purposes), gender, number of cats for whom they serve as primary caregiver, and whether they had access to internet and a smart device with video capabilities. Cat-related questions included the cat’s name, sex, age, ongoing health issues, veterinary visit frequency, whether their mobility challenges had been discussed with a veterinarian and how recently this occurred (within 3 months or more than 3 months ago). If the respondent was the primary caregiver of more than one cat, they were asked to fill out the survey for their cat whose name comes first in the alphabet.

To recruit cat caregivers, an advertisement was distributed using snowball sampling and shared on various social media platforms, emailed to several cat rescue organizations for dissemination, distributed by US and Canadian telehealth platforms (PAWP and Telus MyPet, respectively), and advertised using an article written in Gizmodo, an online news outlet.

### 2.2 Group assignments

Participants who completed the recruitment survey and met the study inclusion criteria were randomly assigned to either the treatment or control group. Cats were blocked for sex, age, and time since their mobility difficulties had been discussed with a veterinarian (less than 3 months ago or more than three months ago/never), then assigned to one of the two study groups using a random number generator (Random UX v.2.1.9).

### 2.3 Initial survey

Caregivers who had been assigned to treatment groups following successful completion of the recruitment questionnaire were invited to participate in the initial study questionnaire [35 questions; Supplementary material 1 (S.1.2)], via email. Questions included asking about their cat’s current medications, supplements, and treatments; human and cat-related barriers to accessing veterinary care; experience with video telehealth; caregiver confidence regarding managing their cat at home; caregiver feelings of support from their veterinary team; willingness to pay for video telehealth appointments; and appointment preference (video telehealth or in-clinic) for addressing various aspects of their cat’s chronic mobility challenges or arthritis. The survey also included a validated clinical metrology instrument, the Feline Musculoskeletal Pain Index – short form [FMPI-sf (23);]. The FMPI-sf has been developed to standardize caretaker reports of cat pain behavior and mobility, and thus was used to assess standardized mobility-related behavior changes between initial and final survey responses. No diagnoses or treatments were suggested to caregivers based on these scores.

### 2.4 Video telehealth visits

Caregivers assigned to the treatment group participated in 6 synchronous video (Zoom Video Communications, Inc., San Jose, California, USA) visits with the same researcher (GB) approximately every 3 weeks over a 4-month period. All video visits were carried out with the primary caregiver(s). One couple completed two visits while on vacation, and their live-in pet sitter collected information on their cat so they could go over it with the researcher. One other cat was re-homed during the study and thus the individual who was their primary caregiver changed. The first appointment took approximately 30–60 min depending on the length of participant responses and the number of questions they had, while the following appointments took approximately 15-30 min each. The last appointment took approximately 60 min for those participants opting to receive the optional educational presentation. The video visits followed a prescribed script [Supplementary material 1 (S.1.3)] which included questions asking how the cat was doing at home and more specific questions about behaviors such as eating, drinking, sleeping/resting, litter box use, play, and jumping behaviors. Caregivers were also asked if they had made any recent modifications to the cat’s environment; if so, they were asked to describe these modifications. These visits adhered to providing tele-advice and educational information to the caregivers and did not involve discussions on medical evaluations, diagnostics, or treatments. If a caregiver asked a medical question, they were requested to speak with their veterinarian.

Participants assigned to the control group did not receive video visits; they completed the initial survey then, 4-months later, completed the final survey.

### 2.5 Body condition scores

Two photos (1-top down, 2-side view) of each cat were requested at the start and the end of the study to visually assess and analyze cat BCS [score: 1–9; (24)]. Caregivers were instructed to take the top-down photo looking directly down at their cat from above, and that either the left or right side of their cat may be used for the side profile view. For both photos they were instructed to have their cat standing and not crouching or sitting, and the background should contrast with the cat’s coat color. BCS reference scores were provided by a single observer (DP) blinded to the assigned group and with extensive experience of feline veterinary care. Given that GB was familiar with cats in the study, a research assistant scored the BCS photos. The assistant received two rounds of BCS training from GB using BCS training information provided by DP. By the second round of training, comparison of DP and the assistant’s BCS scores achieved >0.75% agreement (1.00% = perfect agreement) using Kendall’s Coefficient of Concordance (round 1: W = 0.712, round 2: W = 0.785%). Cat BCS photos were scored in a randomized order.

### 2.6 Final survey

After the initial questionnaire was completed, participants completed the final questionnaire an average of 14.4 (min: 11.6, max: 16.7) weeks later. All participants were emailed a link to the online final study questionnaire [34 questions; Supplementary material 1 (S.1.4)]. The final questionnaire repeated the same questions in the same order as the initial questionnaire, to assess the caregiver’s change in ratings/attitudes after participating/not participating in the video visits.

Study recruitment and data collection took place between July 2023–January 2024.

### 2.7 End of study – educational presentation

After each participant completed the final questionnaire, they were given the optional opportunity to receive a 30-min educational presentation on the home management of cats with chronic mobility conditions (Supplementary material 2). This synchronous virtual presentation was given by GB and contained information on chronic mobility problems, chronic pain and arthritis, as well as adjustments made to at-home management strategies to improve cat comfort and ease of access to resources. This included adjustments made to food and water, litter boxes, grooming, sleeping and resting areas, and play. The presentation also contained education on cat body language and behavior, considerations for caregiver-cat and cat-cat interactions, tips on giving medication, and guidance on seeking veterinary care or advice. This presentation was created in collaboration with DP and CM.

## 3 Statistical analyses

Sample size was estimated using Mead’s Resource Equation and the one-in-ten rule for regression models (25). Descriptive statistics (frequencies and percentages) were produced using statistical software (Jamovi, Jamovi Project, Sydney, Australia) and generated for each survey question and BCS scores. Statistical tests were conducted using SAS Studio (v 3.7, SAS Institute, Cary, North Carolina, USA). For all tests, a *p*-value < 0.05 was considered statistically significant.

Wilcoxon Signed Rank Tests (paired test) were used to assess differences between initial and final survey responses for treatment and control groups for the following Likert-scale variables: confidence in knowledge of cat’s arthritis/mobility needs, perceived helpfulness of current management strategies to keep the cat comfortable in the home, interest in using video telehealth appointments to support their cat’s chronic mobility challenges or arthritis, and willingness to pay for these visits. A Wilcoxon Signed Rank Test was used to assess differences between pre-study and post-study BCS for the treatment and control groups, given graphing of BCS data showed a non-normal distribution; not uncommon for discrete ordinal scales.

A linear mixed model was used to assess the impact of various explanatory variables on FMPI-sf score. Explanatory variables included: experimental group; cat-related data (i.e., age, arthritis diagnosis, etc.); and survey data (initial, final) such as participant ratings on cat knowledge, in-home management, and attitude toward using video telehealth. Respondent identification was included as a random effect and model assumptions were tested and satisfied. The model was built using a backwards stepwise selection technique whereby variables were removed one-by-one based on non-significance (*p* > 0.05). Plausible two-way interactions were tested for inclusion, and the Akaike Information Criterion/Bayesian Information Criterion values (lower value preferred) were used to guide model fit. A Tukey’s adjustment for multiple comparisons was used for *post hoc* comparisons that had 4 or more categories.

## 4 Results

### 4.1 Study participants

One hundred and ninety respondents completed the recruitment survey and fit the inclusion criteria. Of these respondents, 41 withdrew from the study, 16 participants did not complete the final survey, 14 cats passed away during the study, 5 cats were from the same household and thus were not included, 3 were removed due to missing multiple appointments, and 1 cat was expected to be re-homed before study completion and withdrew from the study. Of the remaining 110 participants, 4 were used during the pre-test phase to evaluate the study design and make necessary methodological refinements. The remaining 106 caregivers and their cats participated in the full study; thus the final dataset included 106 cats and their caregivers (treatment = 63, control = 43). The numbers in the two groups are uneven due to participant drop out, which occurred more frequently in the control group.

Participants assigned to the treatment group (*n* = 63) were scheduled to undergo 6 synchronous video visits over a 4-month period, however not all caregivers attended all 6 visits. Attendance for each video visit was as follows: first (*n* = 63, 100%), second (*n* = 62, 98.4%), third (*n* = 61, 96.8%), fourth (*n* = 61, 96.8%), fifth (*n* = 63, 100%), and sixth (*n* = 62, 98.4%). Caregiver responses from questions asked during the 6 video visits (*n* = 372 video appointments) are not included in the current manuscript.

### 4.2 Caretaker demographic and cat information

Many participants self-identified as a woman (79.2%; Table 1), were between the ages of 40–49 (30.2%) or 30–39 (26.4%), owned two (34.0%), one (18.9%), or five or more (18.9%) cat(s), and lived in a suburban (50.0%) or urban (32.1%) area. The majority of respondents were from the United States (89.6%), with 10.4% from Canada. Thirty-two states and the US Virgin Islands (*n* = 95) were represented in our study population, with California residents representing the largest proportion [29.5%; Supplementary material 1 (S.1.5)] of participants. Four Canadian provinces were represented (*n* = 11), with most Canadian participants residing in British Columbia or Nova Scotia (36.4%, each).

**Table 1:** Cat caregiver demographic and cat information (*N* = 106) from the recruitment questionnaire.

Category	Variable	Percentage
Country of residence	United States	89.6
	Canada	10.4
Location of residence	Rural	17.9
	Suburban	50.0
	Urban	32.1
Caregiver age	18–29	7.5
	30–39	26.4
	40–49	30.2
	50–59	16.0
	60–69	13.2
	70 or older	6.6
Caregiver gender	Woman	79.2
	Man	15.1
	Non-binary/third gender	5.7
Caregiver number of cats	One	18.9
	Two	34.0
	Three	16.0
	Four	12.3
	Five or more	18.9
Spay/neuter status	Spayed female	59.4
	Neutered male	40.6
Cat ongoing health issues	Yes	80.2
	No	19.8
Health issue (*n* = 85) *could select multiple*	Neurologic/cognitive	8.2
	Chronic pain	78.8
	Severe dental	12.9
	Skin	5.9
	Heart	10.6
	Kidney	24.7
	Hyperthyroid	8.2
	Diabetes	4.7
	Gastrointestinal	15.3
	Obesity	4.7
	Other	36.5
Veterinary visit frequency *could select multiple*	At least once a year	79.2
	When a health issue arises	38.7
	Every 1–2 years	3.8
	Every 3–5 years	2.8
	Every 5+ years	0.0
	Had cat <1 yr	3.8
Discussed cat’s mobility with veterinarian	No	13.2
	Yes, within last 3mos	55.7
	Yes, more than 3mos ago	31.1
Arthritis or degenerative joint disease diagnosis (*n* = 92)	No	19.6
	Yes	60.9
	Other	19.6

Study cats (*N* = 106) included 43 neutered males and 63 spayed females with an average age of 12.2 years (range: 1–21) years old. Overall, most participants indicated they take their cat to the veterinary clinic at least once per year (79.2%), with 38.7% of participants also stating they take their cat whenever a health issue arises. Additionally, most participants indicated their cat had been diagnosed with an ongoing health issue (85/106, 80.2%), which consisted primarily of chronic pain conditions (67/85, 78.8%). Most caregivers indicated they had discussed their cat’s mobility problems with their veterinarian (86.8%), while 13.2% had not. Of the 92 cats whose mobility challenges had been discussed with a veterinarian, most caregivers had discussed these issues within 3 months of completing the recruitment survey (64.1%), and 60.9% of these cats had been diagnosed with arthritis.

### 4.3 Initial and final questionnaire data

#### 4.3.1 Medications and supplements

Table 2 reports the total number of caregivers providing supplements, medications, and other interventions for their cat’s mobility problems or arthritis, presented by group (treatment *n* = 63, control *n* = 43) and time point (initial and final survey). Supplementary material 1 (S.1.6) presents this data by survey (initial, final; *N* = 106) and presents data describing the length of time (<3 m, 3-6 m, 7-12 m, >1 yr) their cat had been receiving these interventions (initial survey only, *N* = 106). In summary, for both the initial and final surveys, most participants reported that their cat was not currently taking any over-the-counter or non-prescription supplements for their chronic mobility challenges or arthritis (initial control: 83.7%, 36/43; final control: 86.0%, 37/43; initial treatment: 73.0%, 46/63; final treatment: 69.8%, 44/63). At the time of the initial survey, the most common supplement given was glucosamine/chondroitin (control: 14.0%, treatment: 19.0%), followed by omega-3 fatty acids (control: 4.7%, treatment: 11.1%), and for cats in the treatment group, other supplements (11.1%). Results for the final survey indicated participants provided glucosamine/chondroitin (control: 11.6%, treatment: 17.5%), followed by omega-3 fatty acids (control: 2.3%, treatment: 11.1%) and other supplements (control: 4.7%, treatment: 9.5%).

**Table 2:** Interventions provided for cat mobility challenges or arthritis, split by group (treatment *n* = 63, control *n* = 43) and time point (initial and final survey).

Category	Variable	Control initial % (*n*)	Treatment initial % (*n*)	Control final % (*n*)	Treatment final % (*n*)
Supplements	Overall	16.3 (7)	27.0 (17)	14.0 (6)	30.2 (19)
	Glucosamine	14.0 (6)	19.0 (12)	11.6 (5)	17.5 (11)
	Omega-3 s	4.7 (2)	11.1 (7)	2.3 (1)	11.1 (7)
	CBD Products	2.3 (1)	4.8 (3)	0.0 (0)	3.2 (2)
	Green-lipped mussel	2.3 (1)	4.8 (3)	2.3 (1)	1.6 (1)
	Joint diet	2.3 (1)	3.2 (2)	0.0 (0)	3.2 (2)
	Other	2.3 (1)	11.1 (7)	4.7 (2)	9.5 (6)
Medications	Overall	51.2 (22)	54.0 (34)	46.5 (20)	58.7 (37)
	Non-steroidal anti-inflammatory drugs	7.0 (3)	6.3 (4)	4.7 (2)	6.3 (4)
	Gabapentin	18.6 (8)	19.0 (12)	14.0 (6)	17.5 (11)
	Maropitant	9.3 (4)	1.6 (1)	7.0 (3)	3.2 (2)
	Opioids	4.7 (2)	0.0 (0)	4.7 (2)	0.0 (0)
	Tramadol	0.0 (0)	0.0 (0)	0.0 (0)	0.0 (0)
	Amantadine	0.0 (0)	0.0 (0)	0.0 (0)	0.0 (0)
	Frunevetmab	41.9 (18)	34.9 (22)	37.2 (16)	36.5 (23)
	Polysulfated glycosaminoglycan	7.0 (3)	9.5 (6)	4.7 (2)	9.5 (6)
	Other	9.3 (4)	6.3 (4)	4.7 (2)	9.5 (6)
Other interventions	Overall	25.6 (11)	15.9 (10)	25.6 (11)	14.3 (9)
	Laser therapy	7.0 (3)	1.6 (1)	2.3 (1)	3.2 (2)
	Acupuncture	4.7 (2)	6.3 (4)	4.7 (2)	4.8 (3)
	Chiropractic	0.0 (0)	1.6 (1)	0.0 (0)	1.6 (1)
	Massage	0.0 (0)	0.0 (0)	0.0 (0)	3.2 (2)
	Warm/cold compress	7.0 (3)	1.6 (1)	7.0 (3)	1.6 (1)
	Physical therapy	2.3 (1)	1.6 (1)	4.7 (2)	0.0 (0)
	Stem cell therapy	0.0 (0)	0.0 (0)	0.0 (0)	0.0 (0)
	Platelet-rich-plasma	0.0 (0)	0.0 (0)	0.0 (0)	0.0 (0)
	Weight management	16.3 (7)	14.3 (9)	18.6 (8)	9.5 (6)
	Surgery	0.0 (0)	3.2 (2)	0.0 (0)	0.0 (0)
	Other	4.7 (2)	1.6 (1)	4.7 (2)	3.2 (2)
Ability to give oral tablets/pills	Very easy	16.7 (4/24)	26.2 (11/42)	16.7 (4/24)	25.6 (11/43)
	Somewhat easy	25.0 (6/24)	21.4 (9/42)	20.8 (5/24)	14.0 (6/43)
	Neutral	12.5 (3/24)	4.8 (2/42)	12.5 (3/24)	4.7 (2/43)
	Somewhat difficult	25.0 (6/24)	21.4 (9/42)	16.7 (4/24)	18.6 (8/43)
	Very difficult	12.5 (3/24)	26.2 (11/42)	25.0 (6/24)	27.9 (12/43)
	N/A	8.3 (2/24)	0.0 (0)	8.3 (2/24)	9.3 (4/43)
Ability to give oral liquids	Very easy	16.7 (4/24)	19.0 (8/42)	25.0 (6/24)	9.3 (4/43)
	Somewhat easy	25.0 (6/24)	16.7 (7/42)	12.5 (3/24)	23.3 (10/43)
	Neutral	4.2 (1/24)	7.1 (3/42)	8.3 (2/24)	9.3 (4/43)
	Somewhat difficult	33.3 (8/24)	26.2 (11/42)	25.0 (6/24)	27.9 (12/43)
	Very difficult	12.5 (3/24)	21.4 (9/42)	16.7 (4/24)	14.0 (6/43)
	N/A	8.3 (2/24)	9.5 (4/42)	12.5 (3/24)	16.3 (7/43)

Many caregivers indicated their cat received prescription medications for their chronic mobility challenges or arthritis (initial control: 51.2%, final control: 46.5%; initial treatment: 54.0%, final treatment: 58.7%). Frunevetmab (Solensia, Zoetis, Parsippany, NJ, USA) was the most common (initial – control: 41.9%, treatment: 34.9%; final – control: 37.2% treatment: 36.5%), followed by gabapentin (initial – control: 18.6%, treatment: 19.0%; final – control 14.0%, treatment: 17.5%), and polysulfated glycosaminoglycan (Adequan, American Regent Animal Health, Shirley, NY., USA) (initial – control: 7.0%, treatment: 9.5%; final – control: 4.7%, treatment: 9.5%). Other prescriptions made up 14.2% of the medications in the final survey, with prednisolone the most common (6/8, 75%; control: 2, treatment: 4).

Most caregivers reported that they were not currently using any non-medication/supplement treatments (e.g., laser therapy, acupuncture, chiropractic care) for their cat’s chronic mobility challenges or arthritis (initial and final control: 74.4%; initial treatment: 84.1%, final treatment: 85.7%). The most common strategy used was weight management (initial – control: 16.3%, treatment: 14.3%; final – control: 18.6%, treatment: 9.5%). This was followed by veterinary acupuncture for treatment group cats (initial: 6.3%, final: 4.8%), and warm/cold compresses (initial and final: 7.0%) and laser therapy (initial survey only: 7.0%) for control group cats.

Respondents whose cats were taking supplements or prescription medications (initial: 66/106; control: 24, treatment: 42; final: 67/106; control: 24, treatment: 43; Table 2) were asked how easy or difficult it is for them to give oral tablets or pills. Participants initially reported it to be very easy (control: 16.7%, treatment: 26.2%), somewhat easy, or somewhat difficult (control: 25.0%, treatment: 21.4%) or very difficult (control: 12.5%, treatment: 26.2%). In the final survey, they stated it was very easy (control: 16.7%, treatment: 25.6%), somewhat easy (control: 20.8%, treatment: 14.0%), somewhat difficult (control: 16.7%, treatment: 18.6%), or very difficult (control: 25.0%, treatment: 27.9%). Similarly, when asked about the ease of giving oral liquids, they were reported to be very easy (initial – control: 16.7%, treatment: 19.0%; final – control: 25.0%, treatment: 9.3%), somewhat easy (initial – control: 25.0%, treatment: 16.7%; final – control: 12.5%, treatment: 23.3%), somewhat difficult (initial – control: 33.3%, treatment: 26.2%; final – control: 25.0%, treatment: 27.9%), or very difficult (initial – control: 12.5%, treatment: 21.4%; final – control: 16.7%, treatment: 14.0%).

#### 4.3.2 FMPI-sf survey responses

Caregivers reported their cat’s level of mobility using the FMPI-sf scale, and the answers were converted to numerical rankings (normal = 0, not quite normal = 1, somewhat worse than normal = 2, barely, or with great effort = 3, not at all = 4). The FMPI-sf scale responses (average ± SD) for initial survey data were: (treatment: 13.5 ± 6.4, control: 13.8 ± 5.7), and for final survey data were: (treatment: 12.3 ± 7.8, control: 13.2 ± 6.6). A summary of participant responses for each FMPI-sf scale question can be found in Table 3.

**Table 3:** Feline Musculoskeletal Pain Index short form (FMPI-sf) mean ± SD cat scores from the initial and final surveys for each treatment group (control *n* = 43, treatment *n* = 63).

Variable	Survey			
	Control initial (average ± SD)	Treatment initial (average ± SD)	Control final (average ± SD)	Treatment final (average ± SD)
Jump up	2.0 ± 1.1	1.8 ± 0.9	1.7 ± 1.0	1.7 ± 1.1
Jump to kitchen counter	3.3 ± 1.2	3.2 ± 1.2	3.1 ± 1.3	3.0 ± 1.3
Jump down	1.7 ± 1.1	1.7 ± 0.9	1.6 ± 1.2	1.6 ± 1.1
Play with toys	1.7 ± 1.3	1.5 ± 1.2	1.6 ± 1.3	1.3 ± 1.3
Interact other pets	1.8 ± 1.6	1.6 ± 1.5	1.7 ± 1.5	1.3 ± 1.5
Get up	1.0 ± 1.0	1.2 ± 0.9	0.9 ± 1.0	1.0 ± 0.9
Lie down	0.7 ± 0.8	0.7 ± 0.8	0.7 ± 0.8	0.7 ± 0.8
Stretch	0.8 ± 0.9	0.7 ± 1.0	0.7 ± 0.9	0.9 ± 1.2
Groom	0.9 ± 1.2	1.1 ± 1.2	1.1 ± 1.1	1.0 ± 1.1
Total	13.8 ± 5.7	13.5 ± 6.4	13.2 ± 6.6	12.3 ± 7.8

The mixed regression model results suggest an association between FMPI-sf score and interest in using veterinary telehealth for at-home management of their cat’s mobility challenges or arthritis (*p* = 0.0460). Caregivers stating that they were ‘somewhat interested’ tended to have a cat with a lower average (± SD) FMPI-sf score by 2.05 ± 0.77 units compared to those indicating they were ‘very interested’ in using veterinary telehealth for at-home management of their cat’s mobility challenges or arthritis. No other significant effects were detected.

#### 4.3.3 Attitudes towards accessing veterinary care for their cat

**Figure 2:**
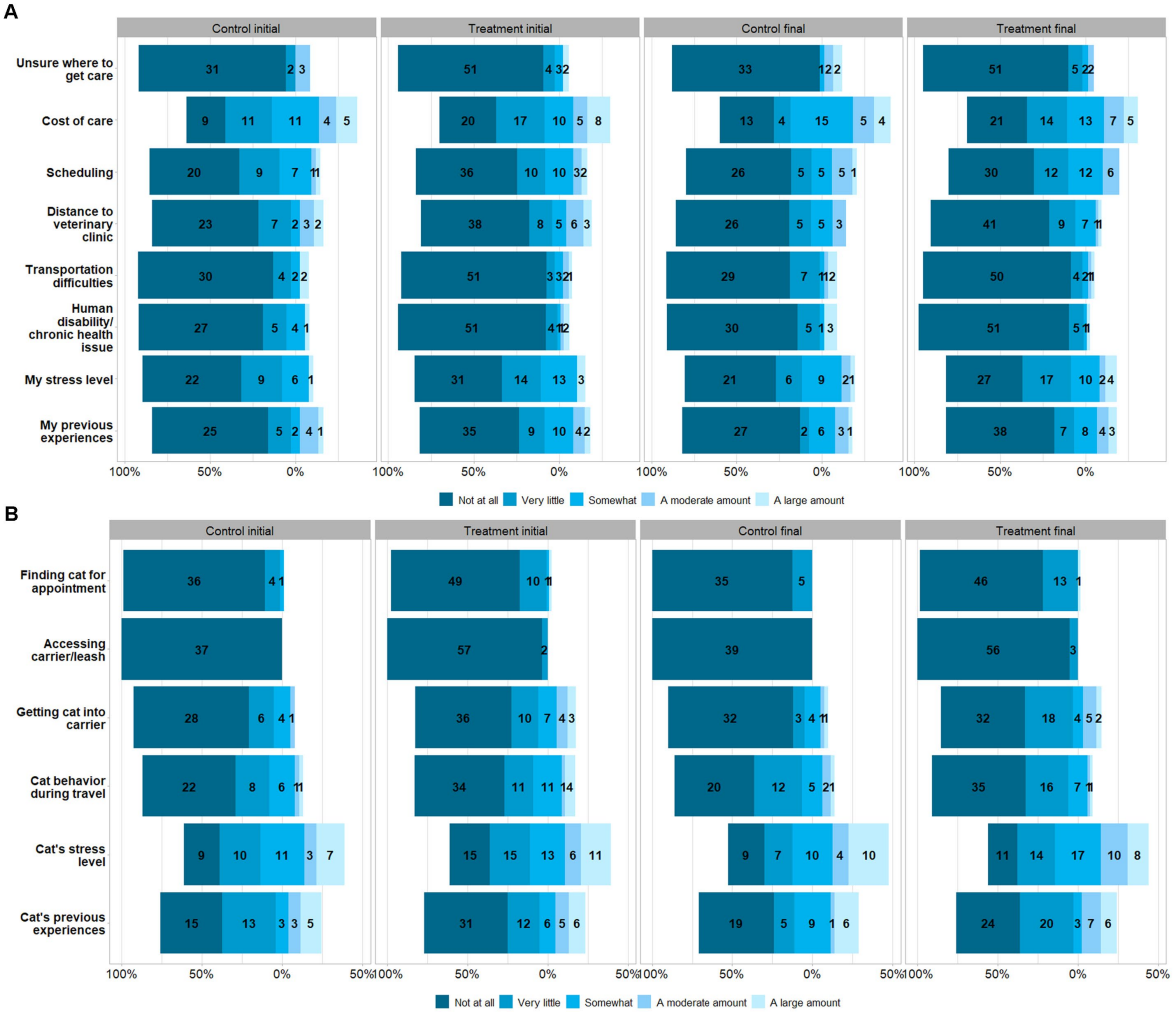
Participants’ initial and final questionnaire Likert-scale ratings of **(A)** caregiver-related, and **(B)** cat-related barriers affecting their access to veterinary care, given as counts and separated by treatment and control groups. The number of responses for each variable differs.

Participants were asked to rate how a range of factors prevent them from accessing veterinary care using a scale between 1 to 5 (1 = not at all, 2 = very little, 3 = somewhat, 4 = a moderate amount, 5 = a large amount). A summary of these data are provided in Figure 2A (caregiver-related barriers) and Figure 2B (cat-related barriers). The barriers frequently reported to have the most impact on access to care were their cat’s stress level, the cost of veterinary care, and their perception of their cat’s past experiences with veterinary appointments. Barriers reported to have the least impact on access to care were being unsure of where to get care, human disability/chronic health conditions, and finding their cat.

Respondents frequently indicated it was very important (initial control: 46.5%, final control: 44.2%; initial and final treatment: 55.6%) for their cat to see a veterinarian for their chronic mobility challenges or arthritis, while others indicated it was somewhat important (initial control: 34.9%, final control: 39.5%; initial and final treatment: 33.3%; Table 4).

**Table 4:** Participant answers for veterinary care & video telehealth questions in each survey, split by group (treatment *n* = 63, control *n* = 43) and timepoint (initial and final survey).

Category	Variable	Control initial % (*n*)	Treatment initial % (*n*)	Control final % (*n*)	Treatment final % (*n*)
Importance of veterinary appointments for mobility	Very important	46.5 (20)	55.6 (35)	44.2 (19)	55.6 (35)
	Somewhat important	34.9 (15)	33.3 (21)	39.5 (17)	33.3 (21)
	Neither	11.6 (5)	7.9 (5)	11.6 (5)	7.9 (5)
	Somewhat unimportant	4.7 (2)	1.6 (1)	4.7 (2)	1.6 (1)
	Very unimportant	2.3 (1)	1.6 (1)	0.0 (0)	1.6 (1)
Used video conferencing before	Yes	90.7 (39)	95.2 (60)	93.0 (40)	93.7 (59)
	No	9.3 (4)	4.8 (3)	7.0 (3)	6.3 (4)
Comfort with video conferencing	Very comfortable	81.4 (35)	61.9 (39)	76.7 (33)	76.2 (48)
	Somewhat comfortable	16.3 (7)	28.6 (18)	16.3 (7)	15.9 (10)
	Neutral	0.0 (0)	4.8 (3)	2.3 (1)	4.8 (3)
	Somewhat uncomfortable	2.3 (1)	1.6 (1)	2.3 (1)	1.6 (1)
	Very uncomfortable	0.0 (0)	3.2 (2)	2.3 (1)	1.6 (1)
Used video telehealth for a pet *could select multiple*	No	88.4 (38)	93.7 (59)	90.7 (39)	84.1 (53)
	Yes, for cat	7.0 (3)	1.6 (1)	4.7 (2)	4.8 (3)
	Yes, for another pet	7.0 (3)	4.8 (3)	4.7 (2)	12.7 (8)
Used video telehealth for self	Yes	79.1 (34)	65.1 (41)	86.0 (37)	66.7 (42)
	No	20.9 (9)	34.9 (22)	14.0 (6)	33.3 (21)
Accessing technology	Very easy	90.7 (39)	85.7 (54)	83.7 (36)	87.3 (55)
	Somewhat easy	7.0 (3)	9.5 (6)	7.0 (3)	6.3 (4)
	Neither	2.3 (1)	4.8 (3)	7.0 (3)	4.8 (3)
	Somewhat difficult	0.0 (0)	0.0 (0)	2.3 (1)	1.6 (1)
	Very difficult	0.0 (0)	0.0 (0)	0.0 (0)	0.0 (0)
Ensuring reliable internet	Very easy	76.7 (33)	73.0 (46)	72.1 (31)	73.0 (46)
	Somewhat easy	16.3 (7)	20.6 (13)	20.9 (9)	17.5 (11)
	Neither	2.3 (1)	6.3 (4)	4.7 (2)	7.9 (5)
	Somewhat difficult	4.7 (2)	0.0 (0)	2.3 (1)	1.6 (1)
	Very difficult	0.0 (0)	0.0 (0)	0.0 (0)	0.0 (0)
Using websites/applications	Very easy	81.4 (35)	73.0 (46)	86.0 (37)	79.4 (50)
	Somewhat easy	16.3 (7)	20.6 (13)	11.6 (5)	14.3 (9)
	Neither	2.3 (1)	6.3 (4)	2.3 (1)	4.8 (3)
	Somewhat difficult	0.0 (0)	0.0 (0)	0.0 (0)	1.6 (1)
	Very difficult	0.0 (0)	0.0 (0)	0.0 (0)	0.0 (0)
Willingness to pay for video telehealth	Much more	0.0 (0)	1.6 (1)	0.0 (0)	0.0 (0)
	A little more	2.3 (1)	1.6 (1)	0.0 (0)	3.2 (2)
	About the same	27.9 (12)	38.1 (24)	25.6 (11)	28.6 (18)
	A little less	34.9 (15)	39.7 (25)	58.1 (25)	49.2 (31)
	Much less	30.2 (13)	15.9 (10)	11.6 (5)	19.0 (12)
	Unwilling to pay	4.7 (2)	3.2 (2)	4.7 (2)	0.0 (0)

Almost all participants had used a video conferencing platform before (initial – control: 90.7%, treatment: 95.2%; final – control: 93.0%, treatment: 93.7%), most were very comfortable with using them (initial – control: 81.4%, treatment: 61.9%; final – control: 76.7%; treatment: 76.2%), and had used video telehealth for their own healthcare (initial – control: 79.1%, treatment: 65.1%; final – control: 86.0%, treatment: 66.7%), but had never had a video telehealth visit with their veterinarian for their cat or another pet (initial – control: 88.4%, treatment: 93.7%; final – control: 90.7%, treatment: 84.1%). The majority of participants indicated it would be very easy for them to access the necessary technology for a telehealth appointment (initial – control: 90.7%, treatment: 85.7%; final – control: 83.7%, treatment: 87.3%), ensure reliable internet (control – initial: 76.7%, final: 72.1%; initial & final treatment: 73.0%), and access the websites or applications needed for an appointment (initial – control: 81.4%, treatment: 73.0%; final – control: 86.0%, treatment: 79.4%).

Caregivers indicated they would be willing to pay a little less (initial – control: 34.9%, treatment: 39.7%; final – control: 58.1%, treatment: 49.2%) or about the same (initial – control: 27.9%, treatment: 38.1%; final – control: 25.6%, treatment: 28.6%) for a video telehealth recheck as they would expect to pay for an in-clinic recheck appointment to help with managing their cat’s mobility challenges or arthritis. Differences in willingness to pay responses were not detected for either the treatment or control group’s initial versus final survey responses (*p* > 0.05).

**Figure 3:**
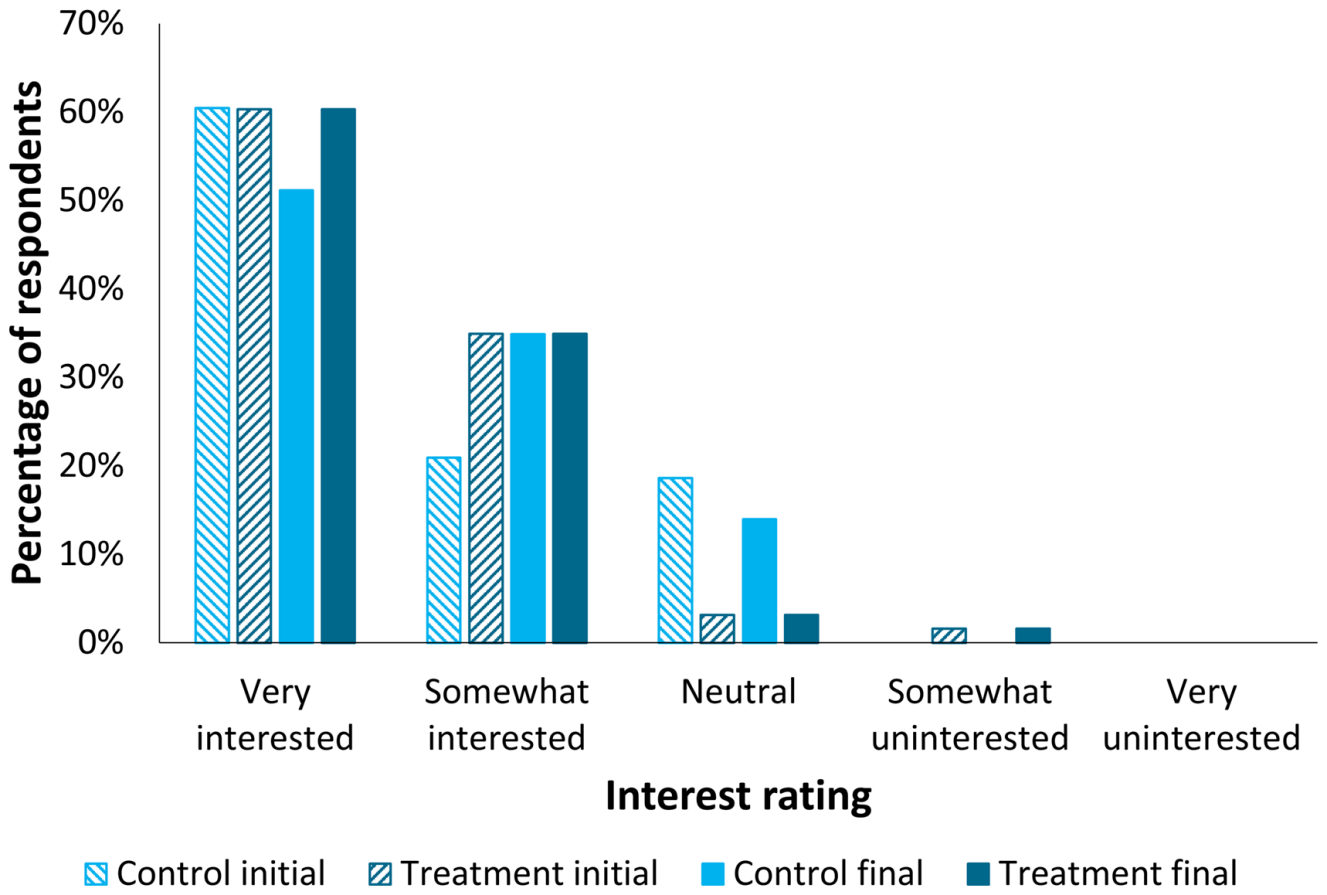
Participant interest in video telehealth appointments for their cat’s mobility challenges or arthritis, by treatment group (treatment *n* = 63, control *n* = 43) and time point (initial and final surveys). Differences were not detected for both the treatment and control group’s initial versus final survey responses (*p* > 0.05; Wilcoxon Signed-Rank Test).

Respondents frequently reported they were very interested (control – initial: 60.5%, final: 51.2%; initial & final treatment: 60.3%) or somewhat interested (control – initial: 20.9%, final: 34.9%; initial & final treatment: 34.9%) in using veterinary video telehealth to help with at-home management of their cat’s chronic mobility challenges or arthritis, if it were available to them (Figure 3). Differences were not detected for either the treatment or control group’s initial versus final survey Likert-scale responses (*p* > 0.05).

**Figure 4:**
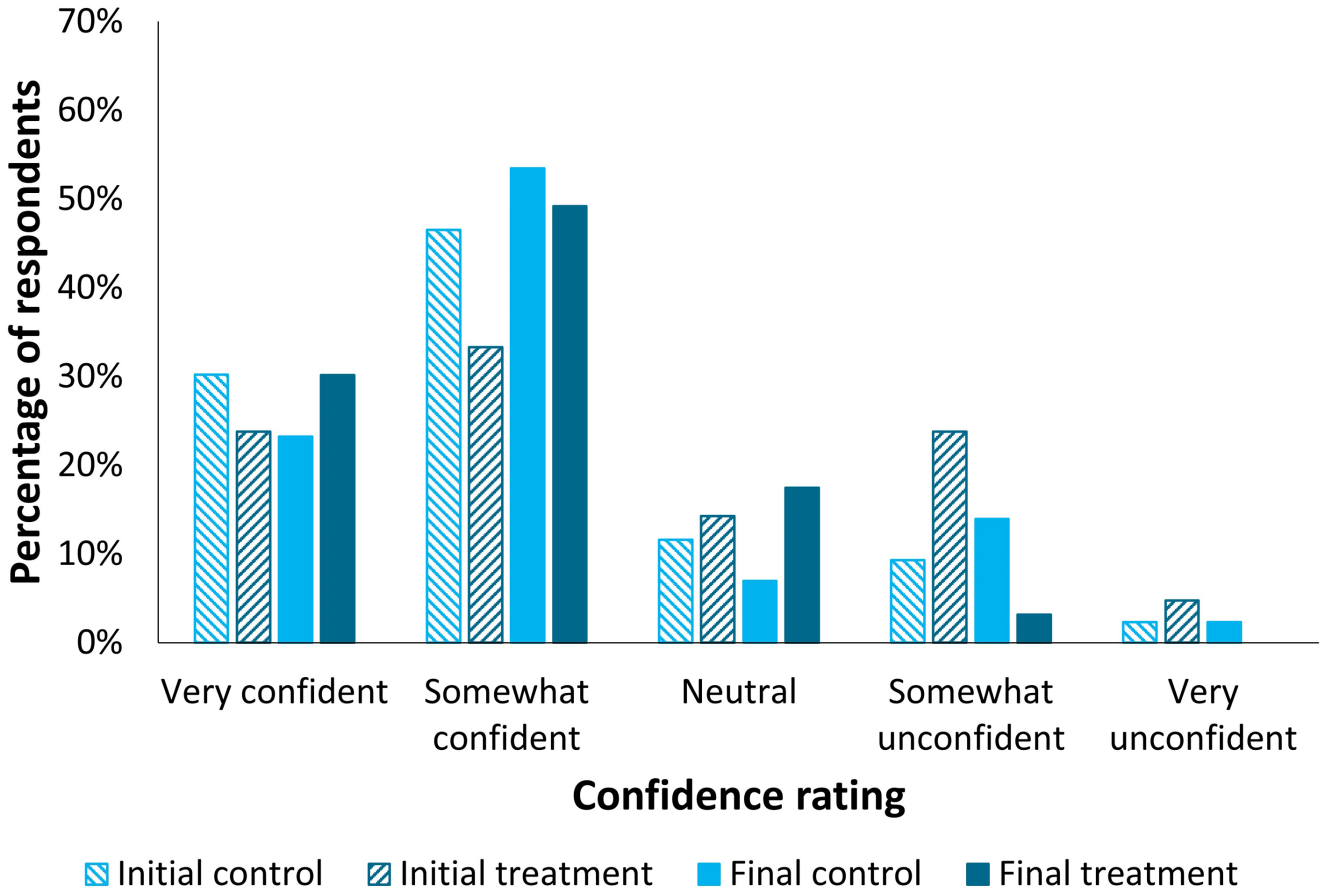
Participant ratings on how confident they feel in their knowledge of their cat’s chronic mobility challenges or arthritis needs, by treatment group (treatment *n* = 63, control *n* = 43) and time point (initial and final surveys). A difference in confidence ratings was detected for the treatment group’s initial versus final survey responses (*p* < 0.0001, Wilcoxon Signed-Rank Test), but not for the control group (*p* > 0.05).

Participants reported that they felt somewhat confident (initial – control: 46.5%, treatment: 33.3%; final – control: 53.5%, treatment: 49.2%; Figure 4) or very confident (initial – control: 30.2%, treatment: 23.8%; final – control: 23.3%, treatment: 30.2%) in their knowledge about their cat’s chronic mobility challenges or arthritis needs. A difference in confidence ratings was detected for the treatment group’s initial versus final survey responses (*p* < 0.0001), but not for the control group (*p* > 0.05). This difference represented an increase in confidence in their own knowledge of their cat’s needs for treatment group participants.

**Figure 5:**
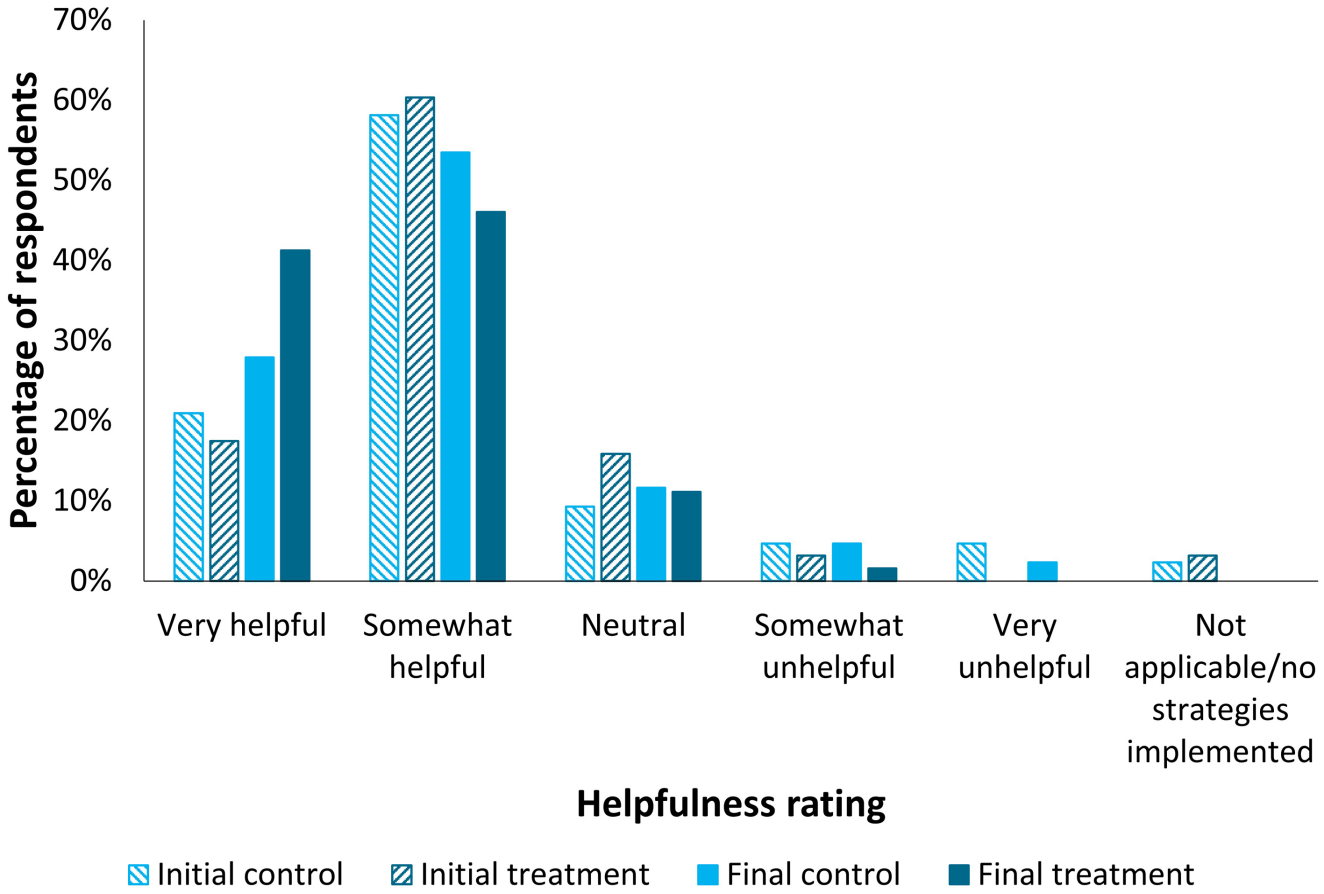
Participant perceptions regarding the helpfulness of their current management strategies for keeping their cat comfortable at home, by treatment group (treatment *n* = 63, control *n* = 43) and time point (initial and final surveys). A difference in Likert-scale ratings was detected for the treatment group’s initial versus final survey responses (*p* = 0.0032, Wilcoxon Signed-Rank Test), but not for the control group (*p* > 0.05).

Most respondents indicated current management strategies were somewhat helpful for keeping their cat comfortable at home (initial – control: 58.1%, treatment: 60.3%; final – control: 53.5%, treatment: 46.0%; Figure 5). A difference in ‘helpfulness of current management strategy’ Likert-scale ratings was detected for the treatment group’s initial versus final survey responses (*p* = 0.0032), but not for the control group (*p* > 0.05). This difference represented an increase in perceived helpfulness of home management strategies for participants in the treatment group.

Caregivers also reported feeling very supported (initial & final control: 37.2%; treatment – initial: 42.9%, final: 46.0%) or somewhat supported (initial & final control: 20.9%; treatment – initial: 17.5%, final: 28.6%) by their veterinarian and veterinary staff in their efforts to care for their cat’s mobility challenges or arthritis.

When asked which appointment type (video telehealth, in-clinic, no preference) they would prefer to address certain aspects of providing care to their cat, participants indicated that they preferred video telehealth for: their cat’s stress level (initial – control: 90.7%, treatment: 87.3%; final – control: 76.7%, treatment: 77.8%; Table 5), implementing environmental changes their veterinarian has recommended for their cat (initial – control: 65.1%, treatment: 63.5%; final – control: 53.5%, treatment: 71.4%), and education about their cat’s mobility challenges or arthritis (initial – control: 53.5%, treatment: 49.2%; final – control: 41.9%, treatment: 69.8%). In-clinic appointments were preferred for physical examinations (initial – control: 93.0%, treatment: 95.2%; final – control: 83.7%, treatment: 96.8%) and diagnosing mobility challenges or arthritis (control – initial: 53.5%, final: 55.8%, initial & final treatment: 50.8%). Many participants reported they had no preference between appointment types for addressing their own stress level (initial – control: 60.5%, treatment: 47.6%; final – control: 41.9%, treatment: 49.2%). Initially, caregivers also reported no preference of appointment type for developing treatment plans for their cat’s mobility challenges or arthritis (control: 53.5%, treatment: 44.4%), or for helping them feel supported and prepared to meet their cat’s needs (control: 60.5%, treatment: 52.4%). While no preference remained the most common choice for those in the control group on the final survey (treatment plans: 53.5%, support: 55.8%), video telehealth was preferred by those in the treatment group (treatment plans: 57.1%, support: 58.7%). Video telehealth and no preference were rated evenly for guidance on giving prescribed medications to their cat initially (control: 44.2% each; treatment: 44.4% & 46.0%, respectively), but video telemedicine was preferred on the final survey (control: 46.5%, treatment: 50.8%). Appointment preference for a recheck on mobility challenges or arthritis was: no preference (initial – control: 44.2%3, treatment: 34.9%; final – control: 32.6%, treatment: 31.7%), video telehealth (initial – control: 34.9%, treatment: 31.7%; final – control: 39.5%, treatment: 38.1%), and in-clinic (initial – control: 20.9%, treatment: 33.3%; final – control: 27.9%, treatment: 30.2%).

**Table 5:** Participant appointment preferences for addressing various aspects of cat care, split by group (treatment *n* = 63, control *n* = 43) and time point (initial and final survey).

Category	Variable	Control initial % (*n*)	Treatment initial % (*n*)	Control final % (*n*)	Treatment final % (*n*)
Cat stress	In-clinic	0.0 (0)	4.8 (3)	9.3 (4)	7.9 (5)
	No preference	9.3 (4)	7.9 (5)	14.0 (6)	14.3 (9)
	Video telehealth	90.7 (39)	87.3 (55)	76.7 (33)	77.8 (49)
Human stress	In-clinic	4.7 (2)	4.8 (3)	18.6 (8)	3.2 (2)
	No preference	60.5 (26)	47.6 (30)	41.9 (18)	49.2 (31)
	Video telehealth	34.9 (15)	47.6 (30)	39.5 (17)	47.6 (30)
Physical examinations	In-clinic	93.0 (40)	95.2 (60)	83.7 (36)	96.8 (61)
	No preference	4.7 (2)	1.6 (1)	7.0 (3)	3.2 (2)
	Video telehealth	2.3 (1)	3.2 (2)	9.3 (4)	0.0 (0)
Diagnosing mobility issues	In-clinic	53.5 (23)	50.8 (32)	55.8 (24)	50.8 (32)
	No preference	25.6 (11)	34.9 (22)	25.6 (11)	34.9 (22)
	Video telehealth	20.9 (9)	14.3 (9)	18.6 (8)	14.3 (9)
Mobility treatment plans	In-clinic	4.7 (2)	6.3 (4)	4.7 (2)	4.8 (3)
	No preference	53.5 (23)	44.4 (28)	53.5 (23)	38.1 (24)
	Video telehealth	41.9 (18)	49.2 (31)	41.9 (18)	57.1 (36)
Medication guidance	In-clinic	11.6 (5)	9.5 (6)	14.0 (6)	6.3 (4)
	No preference	44.2 (19)	44.4 (28)	39.5 (17)	42.9 (27)
	Video telehealth	44.2 (19)	46.0 (29)	46.5 (20)	50.8 (32)
Support to meet cat’s needs	In-clinic	7.0 (3)	6.3 (4)	11.6 (5)	1.6 (1)
	No preference	60.5 (26)	52.4 (33)	55.8 (24)	39.7 (25)
	Video telehealth	32.6 (14)	41.3 (26)	32.6 (14)	58.7 (37)
Mobility education	In-clinic	2.3 (1)	4.8 (3)	4.7 (2)	0.0 (0)
	No preference	44.2 (19)	46.0 (29)	53.5 (23)	30.2 (19)
	Video telehealth	53.5 (23)	49.2 (31)	41.9 (18)	69.8 (44)
Environment changes	In-clinic	0.0 (0)	1.6 (1)	4.7 (2)	0.0 (0)
	No preference	34.9 (15)	34.9 (22)	41.9 (18)	28.6 (18)
	Video telehealth	65.1 (28)	63.5 (40)	53.5 (23)	71.4 (45)
Mobility rechecks	In-clinic	20.9 (9)	33.3 (21)	27.9 (12)	30.2 (19)
	No preference	44.2 (19)	34.9 (22)	32.6 (14)	31.7 (20)
	Video telehealth	34.9 (15)	31.7 (20)	39.5 (17)	38.1 (24)

#### 4.3.4 Body condition scores

We received initial BCS photos from 94.3% (100/106; control: 40, treatment: 60) of participants, and final BCS photos from 85.9% (91/106; control: 36, treatment: 55) of participants. Differences were not detected for either the treatment or control group’s initial versus final BCS data (*p* > 0.05; Wilcoxon Signed-Rank Test). Median (lower quartile, upper quartile) data for each study group and time point (initial, final survey) are as follows: initial control: 6.0 (5.0, 6.0; *n* = 43), initial treatment: 6.0 (5.0, 6.0; *n* = 63), final control: 5.0 (5.0, 6.0; *n* = 43), final treatment: 6.0 (5.0, 6.0; *n* = 63).

### 4.4 Educational presentation

The majority of caregivers (86.8%, 92/106; control: 28, treatment: 55) elected to receive the 30-min educational presentation that was offered at the conclusion of the study. We did not collect data regarding their satisfaction with the presentation, but GB notes that most participants verbally expressed thankfulness at the conclusion of the presentation for the opportunity to receive the information. Anecdotally, many stated they found the information helpful, even if they felt well educated on their cat’s mobility challenges or arthritis and had already implemented many of the strategies presented.

## 5 Discussion

Participant ratings of their knowledge of their cat’s chronic mobility challenges or arthritis and helpfulness of their current at-home management strategies for keeping their cat comfortable improved between initial and final survey responses. These results were specific to treatment group participants who underwent 5–6 video tele-advice visits over a 4-month period and were not detected for control group participants. This research indicates that from the caregiver’s perspective, these appointments were beneficial for both themselves and their cat. Caregivers also indicated an interest in and preference for using video telehealth appointments to receive education on their cat’s mobility challenges or arthritis, and guidance regarding implementing environmental modifications to assist their cat around the home. It is important to note that participants indicated a preference for in-clinic appointments for physical examinations and diagnosing mobility challenges or arthritis, showing that they recognize the need for a hands-on examination for certain aspects of their cat’s care. However, participants’ preference for using virtual video appointments to support other aspects of their cat’s care aligns with previous research. For example, past research also shows caregiver interest in using virtual appointments for receiving medication refills, follow up appointments, and management of chronic conditions, and for reducing their own and their pet’s appointment-related stress (16, 17, 26). Together, these results indicate that caregivers would like the option to use virtual video appointments as a supplement to in-clinic care, while also recognizing the need for in-person appointments. We also found that caregivers who were ‘very interested’ in using veterinary telehealth for at-home management of their cat’s mobility challenges or arthritis had cats with higher mobility impairment, as indicated by their FMPI-sf scores. This suggests that caregivers of cats with more severe mobility problems may be more motivated to use virtual veterinary appointments for their cat.

Virtual appointments, which allow animals and caregivers to remain in the home, may be particularly useful for cats (16, 17). An important benefit of virtual visits is the ability to observe the cat’s normal behavioral repertoire in the home, something which is missed during clinical visits due to stress-induced behavioral changes in which cats may hide signs of mobility-related discomfort or pain (19, 21, 22). While not appropriate for every appointment type, virtual visits may be particularly useful for triage, monitoring, and providing advice and education (20). During these visits, healthcare providers can take questions and provide suggestions on environmental modifications to make cats more comfortable. In addition, these appointments should be relatively easy to conduct, as a large majority of study participants indicated familiarity and comfort with video platforms, and that it was easy for them to access to the technology, internet, and website/applications necessary for a video telehealth visit; consistent with previous research (16, 17, 26). Based on this information, video visits, especially tele-advice appointments, represent a potentially practical and beneficial addition to standard veterinary care for cats.

Most caregivers in this study had not previously used a video telehealth visit for their cat or other pets, although many indicated they had used telehealth for their own healthcare. This aligns with prior research on cat caregivers’ use of video telemedicine appointments (16, 17, 27). While there is a need for more research in this area, surveys of veterinarians have shown that video appointments are not a common form of telehealth provided (21, 28). These data suggest a potential lack of availability/offering of virtual video appointments by veterinary clinics. This may reflect concerns surrounding use of video telehealth technology, such as risks of misdiagnoses and data security (21, 29). Another explanation could be a lack of time for clinicians to devote to telehealth services. However, some virtual telehealth appointments, such as tele-triage or tele-advice visits (which do not involve medical diagnostics, or treatments), do not have to be performed by a veterinarian, and may be performed by knowledgeable non-veterinarian staff members (20). This may be beneficial for these employees, since virtual visits provide the ability for flexible work hours and remote work. In addition, clients may benefit from the ability to schedule appointments outside of traditional work hours. Potential benefits for the practice include improved effectiveness of clinic resources, such as freeing up clinic space and time for other appointment types. However, data are needed to corroborate these potential benefits. We recommend that further research should assess the impact of incorporating non-medical virtual tele-advice appointments into regular clinical practice on pets, clients, clinic staff, and the veterinary practice as a whole.

Overall, average FMPI-sf scores were low for most scale items, with the exception of jumping up to kitchen counter height in one try, which was frequently reported to be ‘impossible’ for cats in this study. This suggests low mobility-related impairment. Other studies have shown greater impairment on parameters such as stretching and grooming than were reported here, however, difficulties with jumping are a common symptom of DJD in cats (7, 8, 11, 23). Most study cats were senior cats [over the age of ten; (30)] and the most commonly diagnosed mobility issue reported was arthritis. This was not surprising, given that radiographic evidence of DJD increases with age and may be prevalent in over 90% of senior cats (5, 19, 31). Many caregivers also reported their cat had chronic pain. Cats experiencing chronic mobility-related pain may display a variety of different and sometimes subtle symptoms, such as: reductions in mobility, activity, and grooming; increased irritability; and/or changes in appetite or vocalizations; which may not be recognized as signs of pain and may be attributed instead to aging (19). Validated questionnaires for assessing an animal’s state, such as the FMPI-sf or the Feline Grimace Scale (FGS), can be valuable additions to a virtual appointment, especially regarding chronic pain, as they can help caregivers objectively and clearly convey information about their cat’s wellbeing to their veterinarian, increasing the utility of the visit. For instance, the FGS has been found to show very good agreement between veterinarians, veterinary nurses, and caregivers who all received the same training on the tool (32).

Low levels of mobility-related impairment may explain why approximately one third of cats in the study were not receiving any interventions to help manage mobility-related challenges, pain, or discomfort. It is possible that caregivers chose not to medicate their cats due to challenges with administering oral medications, as almost half of caregivers reported some level of difficulty. A lack of interventions for these cats could also indicate an underestimation of pain and discomfort by some caregivers and/or their veterinarians. While DJD may result in varying severity of pain, research indicates that identifying mobility-related pain in cats is particularly challenging due to the variety and subtlety of symptoms (33–35), cats masking behavioral signs of mobility-related discomfort and/or pain in a clinic environment (19, 21, 22), and radiographs not identifying DJD (10, 36). Approximately half of the caregivers in our study reported providing their cat with prescription medication for their mobility challenges or arthritis. Frunevetmab (brand name: Solensia), a monoclonal antibody and novel treatment for chronic pain in cats, was the most common medication provided to study cats, representing approximately one-third of our study population. Most participants indicated they did not provide their cat with mobility supplements or therapies such as acupuncture. Veterinary guideline recommendations for cats with DJD list Non-Steroidal Anti-Inflammatory (NSAID) medications, gabapentin, and tramadol as potentially useful analgesic treatments, and suggest non-medication treatments such as massage and acupuncture for painful soft tissue restriction due to DJD or other disease states (6). Virtual appointments, combined with caregiver training and use of validated pain scales such as the FMPI-sf and FGS, may be useful to assess ongoing analgesic and non-medication management of cats with chronic pain conditions. We recommend further research should assess the efficacy of using telehealth technologies to monitors analgesic treatment of cats with DJD.

Most caregivers in the current study reported feeling very supported by their veterinary team and recognized the importance of veterinary care for their cat, which is in agreement with previous findings (16, 17, 26). In addition, participants indicated that they would be willing to pay a little less or about the same amount as an in-clinic appointment for a video telehealth appointment to support their cat’s mobility challenges or arthritis at home. Although we did not see a difference in ratings of willingness to pay between initial and final survey responses for either the treatment or control group, the study results suggest participants value video telehealth appointments regardless of whether they had experienced one or not. Telehealth appointments can be used to provide education, advice, and support to caregivers and are a viable option to assist with providing care to cats in situations when a physical exam is not needed. Considering telehealth as a portion of a holistic, multimodal approach to care, and incorporating home evaluation tools alongside virtual visits may help improve the effectiveness and usefulness of this technology to veterinarians, veterinary staff, caregivers, and their companion animals.

### 5.1 Study limitations

Caregivers in the current study reported relatively few barriers to accessing veterinary care, with cost and cat-related factors rated as higher barriers than other factors. This was a bit surprising and indicates that we likely did not reach a population with major access-to-care difficulties. Given that caregivers consented to participating in a study that was advertised as 15-weeks long, it is also possible our participants may have been more motivated to access veterinary care for their cat’s chronic mobility problems than the general cat caregiver population. It is a risk of selection bias that the study population differs from the target population, something which can be minimized but not completely avoided. Thus, further research focused more specifically on under-served communities is needed to assess the impact of virtual telehealth appointments for under-served and harder to reach cat caregiver populations.

A large majority of caregivers showed an interest in using video telehealth to access cat health care both at the beginning and end of this study. It is likely that study participants were motivated to participate due to an increased interest in veterinary telehealth compared to other cat caregivers. The majority of participants indicated a high level of comfort using video technology which may have also influenced participation. Thus, it is possible our study did not include caregivers who are less comfortable using this type of technology. This could also be a result of selection bias, since recruitment for this study took place entirely online. However, computer technology and video application use has increased in the last few years among both young and older adults (37), suggesting many caregivers could engage in veterinary video telehealth visits. Most of our respondents also elected to receive the educational presentation at the conclusion of the study, which indicates that interest in this incentive may also have motivated participation.

## 6 Conclusion

Overall, our results suggest caregivers caring for cats with chronic mobility challenges and arthritis are interested in video telehealth appointments to assist with managing their cats’ mobility challenges. In addition, undergoing 6 non-medical synchronous video tele-advice appointments over a 4-month period increased participants’ perceived knowledge of their cat’s mobility challenges or arthritis and perceived helpfulness of their at-home management strategies. This suggests that from the caregiver’s perspective, the video tele-advice appointments were beneficial for both themselves and their cat. We recommend further research should assess environmental modifications that are recommended to improve cat comfort, to elucidate how these interventions impact health and behavior outcomes for cats living with chronic mobility problems.

## Data Availability

The raw data supporting the conclusions of this article will be made available by the authors, without undue reservation.
