# Erratum: Incorporating video telehealth for improving at-home management of chronic health conditions in cats: a focus on chronic mobility problems

**DOI:** 10.3389/fvets.2025.1612906

**Published:** 2025-04-29

**Authors:** Frontiers Production Office

**Affiliations:** Frontiers Media SA, Lausanne, Switzerland

**Keywords:** veterinary, feline, virtual, accessibility, access to care

Due to a production error, Figure 1 was omitted from the article, and Figures 2A and 2B were split into Figures 2 and 1, respectively. The corrected Figures 1 and 2, along with their legends, appear below.

**Figure 1:**
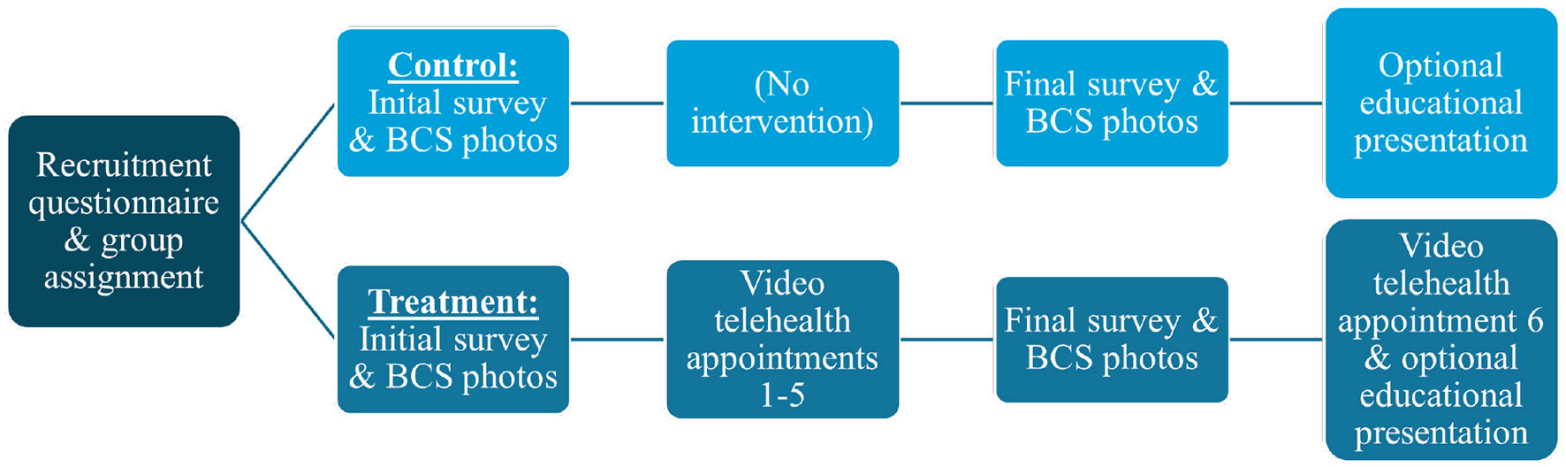
Flowchart showing the sequential process of study participation for cat caregivers in the treatment (*n* = 63) and control (*n* = 43) groups.

**Figure 2:**
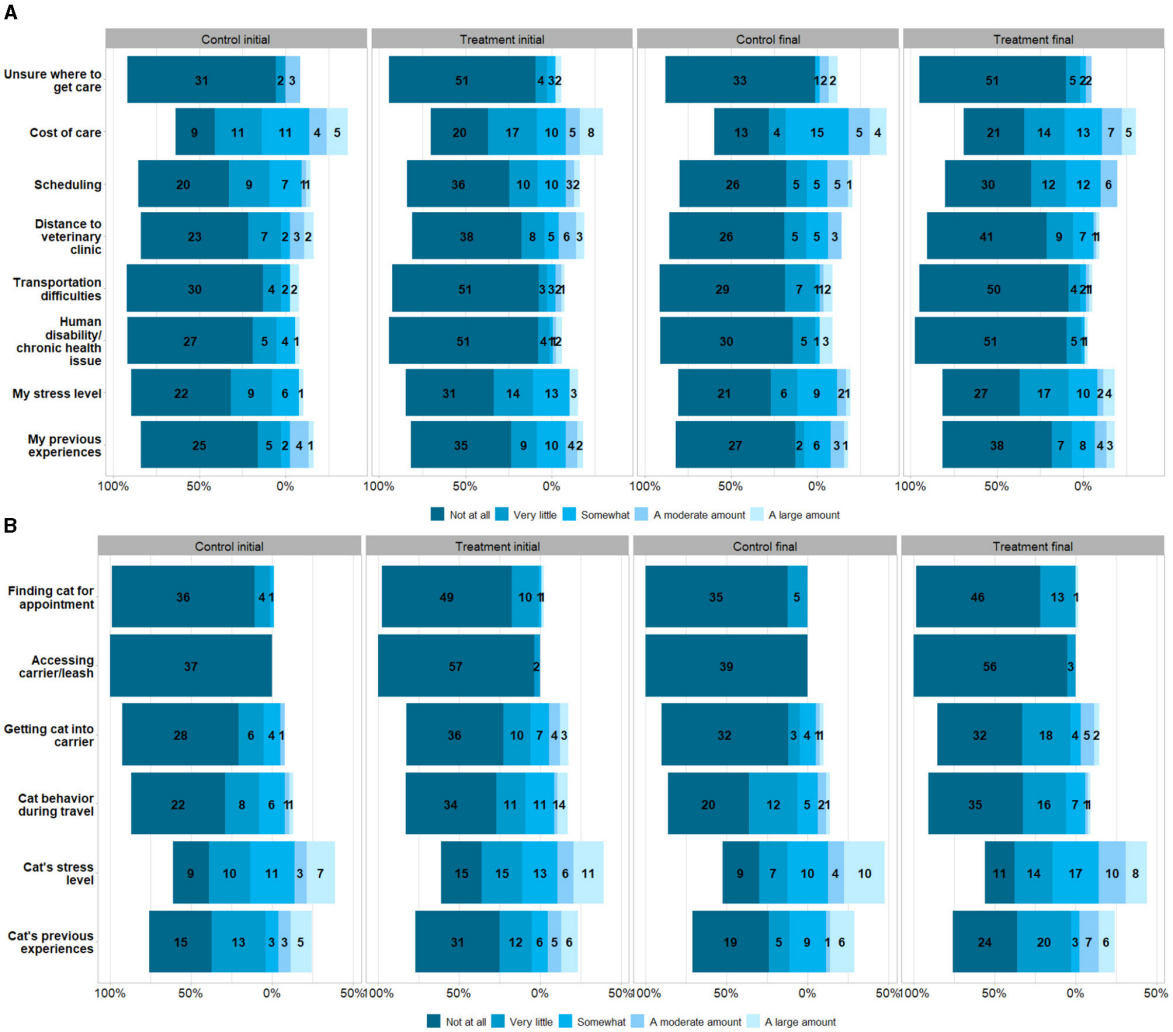
Participants' initial and final questionnaire Likert-scale ratings of **(A)** caregiver-related, and **(B)** cat-related barriers affecting their access to veterinary care, given as counts and separated by treatment and control groups. The number of responses for each variable differs.

The publisher apologizes for this mistake. The original version of this article has been updated.

