# Supplementary material for: Incorporating video telehealth for improving at-home management of chronic health conditions in cats: a focus on chronic mobility problems
Source: Front Vet Sci. 2025 Apr 1;12:1510006. doi: 10.3389/fvets.2025.1510006 (PMC11997976; doi:10.3389/fvets.2025.1510006)
Supplement: Supplementary file 1 [file Supplementary_file_1.docx]

Supplementary Materials 1

S.1.1 - Mobility Recruitment Questionnaire

*Consent Form*

**Use of Veterinary Video Telemedicine for Cats with Chronic Mobility Issues**

**Introduction**

You are invited to join a research study conducted by Dr. Carly Moody (Assistant Professor, Dept of Animal Science, UC Davis), and Grace Boone, MSc (Assistant Specialist) from the University of California, Davis. No prior experience with telemedicine is required to participate in this survey.
Mobility issues, such as those caused by feline degenerative joint disease (DJD) or osteoarthritis (OA) (both referred to as "arthritis" in this study) are a leading chronic health and behavior concern in companion cats. Cats may experience pain and difficulty with everyday behaviors such as grooming, play and litterbox use. These behavior changes may be frustrating or confusing for the cat's caretakers and can feel challenging to manage. Sometimes a cat's pain can go unnoticed, since they can be very skilled at hiding pain or discomfort. This can become an issue for cat welfare, because the cat may be suffering with undetected pain. Telehealth (digital exchange of a pet’s health and/or behavior information and education from a distance) may help increase access to care for cats, reduce follow-up challenges, accessibility barriers, and increase access to education for caretakers.

**Purpose**
The purpose of this study is to investigate video telehealth as a way to provide non-medical advice on at-home management for caretakers of cats suffering with chronic mobility-related challenges.

**Participation and Withdrawal**
Participation in this research is completely voluntary.
Participants must be:

- At least 18 years of age
- Currently residing within the United States, a US territory, or Canada
- The current primary caregiver of at least one companion cat who is living with chronic mobility challenges and spends at least 50% of their time indoors
- Able to access a smart device with video capabilities (e.g., computer, tablet, phone) & internet access

Study participation includes:

- This recruitment survey, which should take 5-10 minutes to complete and will help determine eligibility for the study.
- Assignment to a treatment or control group
- 15-week commitment, which will contain (for all participants):
  - Initial 15 minute survey to gather opinions & demographic information
  - Final survey to gather opinions (15min)
  - Taking photos of your cat (via smartphone, iPad, camera, etc.) and emailing them to us at the beginning and end of the 15 weeks
  - Optional video visit (20min) to answer questions and provide education on feline arthritis at the end of the 15 weeks
  - *Those in the treatment group will additionally receive:*
    - Six video check-ins (one every 3 weeks) with one of our researchers. These check-ins should take approximately 10-15 minutes and will be recorded.
    - The video visits with our team will not involve any veterinary or medical advice, but if we notice anything abnormal with a cat, the caretakers will be informed and directed to contact their veterinarian.

**Risk to Participant**
The risks of this research are minimal. You do not have to answer any of the questions you do not want to answer and you can withdraw at any time without consequence.

**Benefit to Participant**
You will have the opportunity to receive education on at-home management of your cat's mobility challenges. You and your cat should both benefit from this information. Cat health and behavior care communities, as well as other cat caregivers may also indirectly benefit from this research.

**Confidentiality**
As with all research, there is a chance that confidentiality could be compromised; however, we are taking precautions to minimize this risk. Your responses to the surveys will include information that identifies you (your name and email address). This identifiable information will be handled as confidentially as possible. However, individuals from UC Davis who oversee research may access your data during audits or other monitoring activities. Video records of appointments will be kept in password-protected cloud storage and deleted after no later than 10 years. They will not be accessed by anyone except the research team and will not be shared publicly in any way without prior express written permission from participants. We may use the survey data to answer additional research questions or share them with other investigators for additional research. If we do so, we will remove all identifiable information before use or sharing. Once identifiers have been removed, we will not ask your consent for the use of sharing of your data or specimens in additional research.

**Compensation**
You will not be paid for taking part in this study.

**Rights**
Participation in research is completely voluntary. You are free to decline to take part in the project. You can decline to answer any questions (please note: this will prevent you from being able to complete the survey) and you can stop taking part in the project at any time. Whether or not you choose to participate, or answer any question, or stop participating in the project, there will be no penalty to you or loss of benefits to which you are otherwise entitled. If you have any questions about this research, please feel free to contact the researcher, Grace Boone, at gcboone@ucdavis.edu.
Please save or print this page now if you would like a copy for your records.

**Note:** Your responses for this survey will automatically be saved as you go, and you *cannot* go back and change them. If you make an error while completing this survey, please contact us at the email listed above. For the remaining two surveys, your responses will automatically be saved as you go, and you *will* be able to go back and change them.

**If you have read the above information and agree to have you and your cat take part in the research, please click on the arrow button below.**

Please provide your name and email so we can contact you if you and your cat qualify for the study.
Please enter your name here:______________________________________________________
Please enter your email here:_________________________________________________

**Q1**: Are you 18 years of age or older?

- No
- Yes

**Q2:** Do you live within the United States, a US territory, or Canada?

- United States or US territory
- Canada
- Neither

**Display this question if:** Q2 = *“United States or US territory”*

**Q3**: What state or territory do you live in? Please select your state from the list:

▼ Alabama (1) ... Wyoming (56)

**Display this question if:** Q2 = *“Canada”*

**Q4**: What province or territory do you live in? Please select your province from the list:

▼ Alberta (1) ... Yukon (13)

**Q5**: What time zone are you located in?

▼ Guam & Northern Mariana Islands (UTC+10) (15) ... Newfoundland Daylight (UTC-2:30) (8)

**Q6:** To participate, we require that you are the primary caretaker of at least one cat with chronic mobility issues (ongoing for 3 months or more) who spends at least 50% of their time indoors.

Do you have at least one companion cat who is both:

**Living with chronic mobility challenges AND spends at least 50% of their time indoors?**

- No
- Yes

**Q7:** Do you have a smart device with video capabilities (e.g., computer, tablet, phone) and access to the internet?

- Yes
- No

**Q8**: Please provide the following information for your cat.

If you have more than one cat who qualifies, please choose the cat whose name appears first in the alphabet, and list their name, age (approximate if unsure), sex, and basic health information below:
 *For example: If I have two male cats with mobility challenges who spend most of their time indoors, Pebbles and Whiskers, I would list Pebbles here.*

|  | Name: | Age: | Has this cat been diagnosed by a veterinarian with any ongoing health issues? | Spay/neuter status: |
| --- | --- | --- | --- | --- |
| Cat | __ | 1-…21 or older | Yes/No | Spayed female  Neutered male  Intact female |

**Display this question if Q8 =** “*Yes” on ongoing health issues*

**Q9:** Please list the diseases/illnesses a vet has diagnosed for {*Cat’s name*}:
 (Choose all that apply)

- Neurologic/cognitive disease (e.g. seizures)
- Chronic pain (e.g., arthritis, DJD)
- Severe dental disease
- Skin disease (other than fleas, e.g., allergies)
- Heart disease
- Kidney disease
- Hyperthyroid
- Diabetes
- Gastrointestinal Issues (e.g., chronic diarrhea)
- Obesity
- Other (please describe):__________________________________________________

**Q10**: On average, how often does{*Cat’s name*}go to the veterinarian (for any issue)?
 *(select all that apply)*

- At least once a year
- When a health issue arises
- Every 1-2 years
- Every 3-5 years
- Every 5+ years
- I have had this cat for less than 1 year

**Q11**: Have you discussed {*Cat’s name*}'s mobility challenges with a veterinarian?

- Yes, within last 3 months
- Yes, more than 3 months ago
- No

**Display this question if Q11 =** *“Yes, within last 3 months” OR “Yes, more than 3 months ago”*

**Q12**: Did a veterinarian diagnose {*Cat’s name*} with arthritis (also known as osteoarthritis (OA) or degenerative joint disease (DJD))?

- No
- Yes
- Other (please explain)__________________________________________________

Finally, we would like to collect some **demographic information** to help us better understand the ways in which demographics may impact cat healthcare experiences and opinions.

Please answer each question to the best of your ability.

**Q13**: What is your age?

- 18 - 29
- 30 - 39
- 40 - 49
- 50 - 59
- 60 - 69
- 70 or older
- Prefer not to say, but over 18 years of age

**Q14**: Which gender identity do you most identify with?

- Male
- Female
- Non-binary / third gender
- Prefer not to say
- Other__________________________________________________

**Q15**: For how many cats are you the primary caretaker?

- One
- Two
- Three
- Four
- Five or more

**Q16:** Which option best describes where you currently live?

- Urban area (city center or metropolis)
- Suburban (residential area on the outskirts of a city)
- Rural (settled place outside of a city)

**Q17**: Where did you hear about this study?

- Friend or family member
- Social media (e.g., Facebook, Twitter, Instagram)
- Veterinary telemedicine app (e.g., TELUS MyPet, PAWP)
- My veterinary clinic
- Other (please describe)__________________________________________________

Endings:

**Display This Question: If Q1 =** “Yes”**, Or Q2 =** “Neither”**, Or Q6 =** “No”**, Or Q7 =** “No”

Unfortunately you do not qualify for our current study. However, we sincerely appreciate your interest and willingness to participate.

**Display This Question: If Q1 =** “No”**, And Q6 =** “Yes”**, And Q7 =** “Yes”

Thank you for taking our survey, we appreciate your interest and willingness to participate in our research!
Your cat may qualify for inclusion in our study. We will contact you within one week with more information.

**S.1.2 – Mobility Initial Survey**

Welcome to the initial survey for the **Use of Veterinary Video Telehealth for Cats with Chronic Mobility Challenges** study!
We appreciate your continued interest in our work, and your willingness to participate in our study.

Please fill out this questionnaire prior to your first video visit with us and answer each question to the best of your ability. If you make a mistake, you can use the back button to return to previous questions. This survey should take 10-15 minutes to complete.

Thank you!

**Q1a:** We will be video-recording the Zoom visits in order to collect data from the videos. Do you consent to allow us to record you and your cat?

- Yes
- No

**Q1b**: It is helpful for us to be able to use portions of the videos for academic presentations, social media, and other media outlets to share our research results with others. Do you consent to allow us to use video clips of you and your cat in this way?

- Yes
- Yes, but please crop me out or blur my face
- No

**Q2:** Please type your cat's name here:_______________________________________________

**Q3:** Please type your email here:___________________________________________________

The following questions will provide us some general information about your cat and their mobility challenges or arthritis.

Please answer each question to the best of your ability. If you make a mistake, you can use the back button to return to previous questions.

**Q4:** Is {*Cat’s name*} currently taking any over-the-counter, or non-prescription supplements (e.g., glucosamine/chondroitin, therapeutic diets) for their chronic mobility challenges or arthritis?

- Yes
- No

**Display this question if Q4 =** *Yes*

**Q5:** Please list how long you have been giving {*Cat’s name*} any of the following **supplements** to help manage their chronic mobility challenges or arthritis.

|  | Less than 3 months | 3-6 months | 7-12 months | More than a year | Not currently taking this supplement |
| --- | --- | --- | --- | --- | --- |
| Glucosamine/chondroitin (e.g., Dasuquin or Cosequin) |  |  |  |  |  |
| Omega-3 fatty acids |  |  |  |  |  |
| Cannabidiol (CBD) products |  |  |  |  |  |
| Green-lipped mussel |  |  |  |  |  |
| Joint diet (e.g., Mobility Support) |  |  |  |  |  |
| Other (please list) |  |  |  |  |  |

**Q6:** {*Cat’s name*} currently receiving or taking any prescription medications (e.g., gabapentin, NSAIDs, Solensia, Adequan) for their chronic mobility challenges or arthritis?

- Yes
- No

**Display This Question if:** Q6 = Yes

**Q7:** Please list how long you have been giving {*Cat’s name*} any of the following medications to help manage their chronic mobility challenges or arthritis.

|  | Less than 3 months | 3-6 months | 7-12 months | More than a year | Not currently taking this medication |
| --- | --- | --- | --- | --- | --- |
| Non-steroidal anti-inflammatories (NSAIDS), e.g., meloxicam, Onsior® (robenacoxib) |  |  |  |  |  |
| Gabapentin |  |  |  |  |  |
| Cerenia® (maropitant citrate) |  |  |  |  |  |
| Opioid pain medications (e.g., buprenorphine) |  |  |  |  |  |
| Tramadol |  |  |  |  |  |
| Amantadine |  |  |  |  |  |
| Solensia® (frunevetmab), a monoclonal antibody treatment |  |  |  |  |  |
| Adequan® (Polysulfated glycosaminoglycan) |  |  |  |  |  |
| Other (please list) |  |  |  |  |  |

**Q8:** Is {*Cat’s name*} currently utilizing any non-medication/supplement treatments (e.g., acupuncture, weight management, physical therapy) for their chronic mobility challenges or arthritis?

- Yes
- No

**Display This Question:** if Q8 = Yes

**Q9:** Please list how long you have been using any of the following treatments to help {*Cat’s name*} manage their chronic mobility challenges or arthritis.
 *(For surgery, times represent how long ago surgery was done, if applicable.)*

|  | Less than 3 months | 3-6 months | 7-12 months | More than a year | Not currently using this treatment |
| --- | --- | --- | --- | --- | --- |
| Laser therapy/cold laser |  |  |  |  |  |
| Veterinary acupuncture |  |  |  |  |  |
| Veterinary chiropractic care |  |  |  |  |  |
| Medical massage |  |  |  |  |  |
| Warm/cold compresses |  |  |  |  |  |
| Physical therapy (incl. hydrotherapy) |  |  |  |  |  |
| Stem cell therapy |  |  |  |  |  |
| Platelet-rich-plasma (PRP) |  |  |  |  |  |
| Weight management |  |  |  |  |  |
| Surgery (e.g., femoral head ostectomy or FHO, wrist fusion) |  |  |  |  |  |
| Other (please list) |  |  |  |  |  |

FMPI intro **Feline Musculoskeletal Pain Index - short form**

 Please take some time to complete the following questions.
 Please mark the circle that best describes your cat's ability to perform the following activities.

 *(Copyright 2020 North Carolina State University, reproduced with permission)*

**Q10:** 1. Jump up?

- Normal
- Not quite normal
- Somewhat worse than normal
- Barely, or with great effort
- Not at all

**Q11:** 2. Jump up to kitchen-counter height in one try?

- Normal
- Not quite normal
- Somewhat worse than normal
- Barely, or with great effort
- Not at all

**Q12:** 3. Jump down (how well and how easily)?

- Normal
- Not quite normal
- Somewhat worse than normal
- Barely, or with great effort
- Not at all

**Q13:** 4. Play with toys and/or chase objects?

- Normal
- Not quite normal
- Somewhat worse than normal
- Barely, or with great effort
- Not at all

**Q14:** 5. Play and interact with other pets?

- Normal
- Not quite normal
- Somewhat worse than normal
- Barely, or with great effort
- Not at all

**Q15:** 6. Get up from a resting position?

- Normal
- Not quite normal
- Somewhat worse than normal
- Barely, or with great effort
- Not at all

**Q16:** 7. Lie and/or sit down?

- Normal
- Not quite normal
- Somewhat worse than normal
- Barely, or with great effort
- Not at all

**Q17:** 8. Stretch?

- Normal
- Not quite normal
- Somewhat worse than normal
- Barely, or with great effort
- Not at all

**Q18:** 9. Groom himself or herself?

- Normal
- Not quite normal
- Somewhat worse than normal
- Barely, or with great effort
- Not at all

The following questions will focus on your experiences with and opinions on, using **in-clinic** veterinary appointments for {*Cat’s name*}'s chronic mobility challenges or arthritis.

Please answer each question to the best of your ability. If you make a mistake, you can use the back button to return to previous questions.

**Q19:** How do you feel about veterinary appointments for {*Cat’s name*}'s chronic mobility challenges or arthritis?

- It is very important for {Cat’s name} to see a veterinarian for this
- It is somewhat important for {Cat’s name} to see a veterinarian for this
- It is neither important nor unimportant for {Cat’s name} to see a veterinarian for this
- It is somewhat unimportant for {Cat’s name} to see a veterinarian for this
- It is very unimportant for {Cat’s name} to see a veterinarian for this

**Q20:** Do any of the following **human-related factors** prevent you from accessing veterinary care for {Cat’s name}'s chronic mobility challenges or arthritis?
 *(Please move the slider to the number on the slide that is most accurate.)*

|  |  | Not at all | Very  little | Somewhat | Moderate  amount | Large  amount |
| --- | --- | --- | --- | --- | --- | --- |
|  | 0 | 1 | 2 | 3 | 4 | 5 |

| Not sure where to get veterinary care | 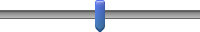 |
| --- | --- |
| Cost of veterinary care | 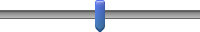 |
| Finding appointments that work with my schedule | 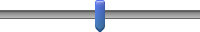 |
| Distance from/to preferred veterinary clinic | 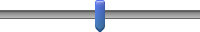 |
| Transportation challenges (e.g., vehicle availability, human mobility/injury, etc.) | 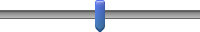 |
| Living with a disability or chronic health condition | 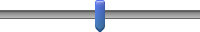 |
| My stress level | 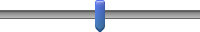 |
| My past experiences with clinic visits | 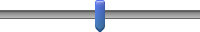 |

**Q21:** Do any of the following **cat-related factors** prevent you from accessing veterinary care for {Cat’s name}'s chronic mobility challenges or arthritis?
 *(Please move the slider to the number on the slide that is most accurate.)*

|  |  | Not at all | Very  little | Somewhat | Moderate  amount | Large  amount |
| --- | --- | --- | --- | --- | --- | --- |
|  | 0 | 1 | 2 | 3 | 4 | 5 |

| Finding my cat for their appointment | 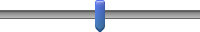 |
| --- | --- |
| Access to a carrier or leash for my cat | 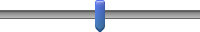 |
| Getting my cat into the carrier or leash | 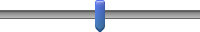 |
| Cat behavior during travel to the clinic | 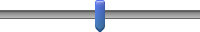 |
| Cat's stress level | 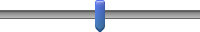 |
| Cat's past experiences with clinic visits | 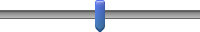 |

The following questions will focus on your experiences with and opinions on, **video conferencing software and telehealth** appointments.

Please answer each question to the best of your ability. If you make a mistake, you can use the back button to return to previous questions.

**Q22:** Have you ever used a video conferencing platform (e.g., Zoom, Facetime, Skype, Microsoft Teams, Google Meet) before?

- No
- Yes

**Q23:** How comfortable are you with using video conferencing platforms (e.g., Zoom, Facetime, Skype, Microsoft Teams, Google Meet)?

- Very comfortable
- Somewhat comfortable
- Neutral
- Somewhat uncomfortable
- Very uncomfortable

**For the following questions, please keep this definition in mind:** Telehealth is the overarching term that encompasses all uses of technology to remotely gather and deliver health information, advice, education, and care.

**Q24:** Have you ever had a **video telehealth** visit for a pet?

In other words, have you ever used video conferencing software such as Zoom, Facetime, Skype, Microsoft Teams, Google Meet, with your veterinarian?
 *(Please select all that apply)*

- Yes, for {*Cat’s name*}
- Yes, for a pet other than {*Cat’s name*}
- No

**Q25:** Have you ever had a **video telehealth** visit for yourself?

 In other words, have you ever used video conferencing software such as, but not limited to, Zoom, Facetime, Skype, Microsoft Teams, Google Meet, with a healthcare provider (i.e. physician, nurse, dermatologist, etc.)?

- Yes
- No

**Q26:** Please rate your ability to access the following, for a veterinary video telehealth appointment:

|  | Very easy | Somewhat easy | Neither easy nor difficult | Somewhat difficult | Very difficult |
| --- | --- | --- | --- | --- | --- |
| Accessing the necessary **technology** (e.g., computer, tablet, smartphone, camera/webcam) for an appointment is: |  |  |  |  |  |
| Ensuring **strong,** **reliable internet connection** for an appointment is: |  |  |  |  |  |
| Accessing and using the **websites/apps** needed for an appointment is: |  |  |  |  |  |

**Q27:** If veterinary **video telehealth** were available to you, please rate your interest in using it to help with at-home management of {Cat’s name}'s chronic mobility challenges or arthritis?

- Very interested
- Somewhat interested
- Neutral
- Somewhat uninterested
- Very uninterested

**Q28:** In comparison to what you would expect to pay for a normal in-clinic veterinary recheck appointment (to help with chronic mobility challenges or arthritis management for {Cat’s name}), how much are you willing to pay for a video telehealth recheck?

- I would not be willing to pay for this service
- Much less
- A little less
- About the same
- A little more
- Much more

The following questions will focus on your **at-home experiences** with {Cat’s name}.

Please answer each question to the best of your ability. If you make a mistake, you can use the back button to return to previous questions.

**Q29:** Overall, how confident do you feel in your knowledge about {Cat’s name}'s chronic mobility challenges or arthritis needs?

- Very confident
- Somewhat confident
- Neutral
- Somewhat unconfident
- Very unconfident

**Q30:** How supported do you feel by your veterinarian and veterinary staff in your efforts to care for {Cat’s name}'s chronic mobility challenges or arthritis?

- Very unsupported
- Somewhat unsupported
- Neutral
- Somewhat supported
- Very supported
- Not applicable

**Q31:** How helpful do you feel your **current** management strategies are for keeping {Cat’s name} comfortable at home?

- Very helpful
- Somewhat helpful
- Neutral
- Somewhat unhelpful
- Very unhelpful
- Not applicable/no strategies implemented

**Display This Question:** If Q4 = *Yes* **Or** Q6 = Yes

**Q32:** How easy or difficult is it for you to give {Cat’s name}'s medications or supplements by mouth (pills/tablets, liquids)?
 *Note: This does not include medications mixed in with food.*

|  | Very Difficult | Somewhat difficult | Neutral | Somewhat easy | Very easy | Not applicable |
| --- | --- | --- | --- | --- | --- | --- |
| Oral tablets/pills |  |  |  |  |  |  |
| Oral liquids |  |  |  |  |  |  |

**Q33:** Which would you prefer, video telehealth or in-clinic, for addressing the following aspects of {Cat’s name}'s chronic mobility challenges or arthritis:

|  | Video telehealth | In-clinic | No preference |
| --- | --- | --- | --- |
| Cat's stress level |  |  |  |
| My stress level |  |  |  |
| Physical examinations |  |  |  |
| Diagnosing my cat's mobility challenges/arthritis |  |  |  |
| Developing treatment plans for my cat's mobility challenges/arthritis |  |  |  |
| Providing guidance on giving prescribed medications to my cat |  |  |  |
| Helping me feel supported & prepared to meet my cat's needs |  |  |  |
| Education about my cat's mobility challenges/arthritis |  |  |  |
| Implementing environmental changes my veterinarian has recommended for my cat |  |  |  |
| Rechecks for my cat's mobility challenges/arthritis |  |  |  |

**Q34:** Is there anything else you would like to add about your experiences with in-clinic or video telehealth for managing {Cat’s name}'s chronic mobility challenges or arthritis?

________________________________________________________________

________________________________________________________________

________________________________________________________________

________________________________________________________________

________________________________________________________________

S.1.3 – Appointment Script

**Visit Script**

Welcome

- “Hello (participant and cat’s names), and welcome to our cat chronic mobility challenges/arthritis project!

My name is Grace, I am an Assistant Specialist at the University of California, Davis, and I will be conducting the video appointments for this project.

We would like to record these appointments so we can analyze the data later and get screenshots of the appointments for publications, as mentioned in the consent form of the recruitment survey you filled out online. Do you consent to being recorded during these appointments?”

- - Yes: move on
  - No: unable to proceed
- Webcam
  - If webcam on and well-positioned:

“It looks like your webcam is working great, and I should be able to see you and your cat, so we are good to go there!”

- - If webcam on but ill-positioned:

“It looks like your webcam is working great, but I will need you to move the camera… (direct as needed to see person, and their cat, if present)”.

- - If webcam off:

“We would like to be able to capture portions of these video appointments for publications or academic presentations. Do you mind turning on your video?” (Then continue with positioning as above)

- - If webcam doesn’t work:
    Try to reschedule.
  - If decline:
    Unable to continue.

*For initial visit, all questions were asked with regard to the cat’s mobility and overall changes, for follow-up visits, changes were regarding the past 3 weeks.*

Questions:

- “Alright, let’s get started!
- “On a scale of one to five, with one being very poorly, two somewhat poorly, three neutral, four somewhat well and five being very well, how has (cat’s name) been doing at home?”
  - Record answer:
  - Give them time to explain.
- “Do you know what breed your cat is?” *first visit only
  - Record answer
- For cats with a mobility discussion in last 3 months *first visit only:
  “It looks like you said in your recruitment survey that you discussed (cat’s name)’s chronic mobility challenges/arthritis with your veterinarian within the last three months. Do you know the exact date of that visit?”
  - Give them time to answer.
    - If yes: record date
    - If no: move on
- “Has (cat’s name) had an in-clinic veterinary visit related to their chronic mobility challenges/arthritis since our last video visit?” *follow-up visits only
  - Record yes/no
  - If yes: give time to explain & take notes.
- “Have you noticed any changes in (cat’s name)’s behavior since their most recent veterinary visit for their chronic mobility challenges/arthritis?” *first visit only
  - Record yes/no
  - Give them time to explain & take notes.
- “Have you noticed any changes in (cat’s name)’s behavior since our last video visit?” *follow-up visits only
  - Record yes/no
  - If yes: give time to explain & take notes.
- “Next, I am going to ask more specific questions about several aspects of (cat’s name)’s daily life that may have been impacted by their chronic mobility challenges/arthritis. You may have seen changes in all or none of these areas, and either is okay! I will also ask if you have made changes to (his/her) environment, but this is just to get an idea of how their environment and daily life may have changed because of their chronic mobility challenges/arthritis. There are no right or wrong answers to any of these questions!”
  - “How would you rate (cat’s name)’s overall mobility? Low, medium or high?”
    - Record answer (low, med, high).
    - Give them time to explain.
  - “Is (he/she) still jumping up to places (he/she) likes to sit, such as the bed or their cat tree?”
    - Record yes/no
    - If no: give time to explain & take notes.
    - “Have you made any changes to (cat’s name)’s environment to help (him/her) access higher spots?”
      - Record yes/no
      - If yes: give time to explain & take notes.
  - “Have you noticed any changes to (cat’s name)’s eating or drinking behaviors?”
    - Record yes/no
    - If yes: “Are they eating or drinking more or less than they were?”
      - Record more/less. Give time to explain & take notes.
    - Have you made any changes to (his/her) food or water setup?
      - Record yes/no
      - If yes: give time to explain & take notes.
  - “Is (cat’s name) using their litter box normally?”
    - Record yes/no
    - If no: give time to explain & take notes.
    - “Have you noticed any issues for (him/her) getting into or out of the litter box?”
      - Record yes/no
      - If yes: give time to explain & take notes.
    - “Does (he/she) have any difficulties accessing their litter box wherever it is in the house?”
      - Record yes/no
      - If yes: give time to explain & take notes.
    - “Have you made any changes to (his/her) litter box setup?”
      - Record yes/no
      - If yes: give time to explain & take notes.
  - “Have you noticed any changes in (cat’s name)’s resting behaviors?
    - Record yes/no
    - If yes: “Is (he/she) resting more or less than they were?”
      - Record more/less. Give time to explain & take notes.
    - “Have you made any changes to (his/her) sleeping or resting spots?”
      - Record yes/no
      - If yes: give time to explain & take notes.
  - “Does (cat’s name) meow, growl or hiss more, less, or the same as they did?
    - Record more/less/same
    - If changed: give time to explain & take notes.
  - “Is (cat’s name) more or less cuddly than they were, or the same?”
    - Record more/less/same
    - If changed: give time to explain & take notes.
  - “Does (cat’s name) like to be picked up more, less, or the same amount?
    - Record more/less/same
    - If changed: give time to explain & take notes.
  - “Does (cat’s name) like to be pet more, less, or the same amount?”
    - Record more/less/same
    - If changes: give time to explain & take notes.
  - “Does (cat’s name) prefer different spots for petting?”
    - Record yes/no
    - If yes: give time to explain & take notes.
  - “Have you noticed any changes in (cat’s name)’s grooming behaviors?”
    - Record yes/no
    - If yes: “Do they groom themselves more or less?”
      - Give time to explain & take notes.
  - “Have you noticed any changes in (cat’s name)’s play behaviors, for example: “zoomies” or playing with toys?”
    - Record yes/no
    - If yes: “Do they play more or less?”
      - Record more/less.
    - “Do they prefer different toys?”
      - Record yes/no.
      - If yes, give them time to explain.
  - “Have you noticed any changes in (cat’s name)’s interactions with other pets in your home
    - Record yes/no
    - If yes: “Are they more or less cuddly than they were?”
      - Record yes/no
    - “Do they like to play together more or less than they did?”
      - Record more/less, give them time to explain.
  - “Have you noticed any changes in (cat’s name)’s overall activity level?”
    - Record yes/no
    - If yes: give time to explain & take notes.
  - If on medications:
    “Are you having any difficulties with giving (cat’s name) their medications?”
    - Record yes/no
    - If yes: give time to explain & take notes.
- “Is there anything else you think I should know about (cat’s name), or about their chronic mobility challenges/arthritis?”
  - Record yes/no
  - If yes: give time to explain & take notes.
- “Do you have any concerns about (cat’s name)?”
  - Record yes/no
  - If yes: give time to explain & take notes
    Medical concerns: direct caretaker to talk to a veterinarian.
- “Any other questions or anything else you want to chat about?”
  - Record yes/no
  - If yes: give time to explain & take notes.
- “Great, thank you! We will see you in three weeks for your next video check-in.

Suggestions (if they had concerns, choose the appropriate suggestions only)

- High area access
  - “I noted that you mentioned earlier (cat’s name) has difficulties with jumping or accessing high areas. One thing that may help (him/her) with that is adding steps or a ramp to help them get up on the (bed, couch, etc.)”
- Litterbox
  - “I noted that you mentioned earlier (cat’s name) has difficulties with accessing their litter box. Some things that can help arthritic cats to better use their litter boxes are to cut down the sides or change to a box with lower sides, so they don’t have to step over a tall box edge to get into the box. Having an extra-large box so there is plenty of room for them to make wider turns inside the box can also be helpful. Taking the covers off of covered boxes may help as well. You could also consider adding a mat outside/around the box for traction. Finally, if your home has more than one level, keeping the litter boxes on the same floor as the main living area will reduce the need for your cat to use stairs to access their litter box. If possible, place a box on each floor of the home. Having multiple litter box options so they are easily accessible for the cat (especially in a larger home), is also useful.”
- Food/water
  - “I noted that you mentioned earlier (cat’s name) has difficulties with their food and water. You might consider elevating their bowls a bit, so they don’t have to bend down or reach their neck to access the bowl’s contents. If your home has more than one level, it would be best to have their food and water located on the main floor of the home to minimize the need for them to go up or down the stairs. It would also be ideal to have a water bowl available on each floor of the home.”
- Bedding
  - “I noted that you mentioned earlier (cat’s name) seems extra stiff when they get up from a nap or seems uncomfortable resting. Soft bedding can be helpful for arthritic cats, so if they don’t already have some, consider adding some soft blankets to areas they like to rest. Heat can also be helpful for a variety of things, including arthritic joint pain, so a heated mat/pad made for pets may be a good addition. If you aren’t comfortable with heating pads, you can warm up towels or blankets in the dryer for a few minutes.”
- Interactions & handling
  - “I noted that you mentioned earlier (cat’s name) seems more challenging to handle/you are concerned about handling (cat’s name) because of their chronic mobility challenges/arthritis.”
    - “If you can, avoid picking up (cat’s name). When it is necessary, go slow, support them and avoid placing pressure on painful areas.”
    - “When petting (cat’s name), go slow, use a light touch, and avoid painful areas.”
    - “For nail trims and other things that may use restraint, less is more. Low stress handling methods such as passive restraint and towel wraps, as opposed to scruffing, can be helpful. Let me show you some examples of what this looks like.” *Show photos and descriptions from educational presentation.*
    - “Pay attention to the cat’s body language and be alert for signs of negative arousal/stress (e.g., ears to the side or back, dilated pupils, increased breathing rate, struggling or vocalizing, lip-licking without a food or grooming reason), and respect your cat’s choices (e.g., give them a break from petting, set them down, etc.)”

S.1.4 - Mobility Final Survey

Welcome to the final survey for the **Use of Veterinary Video Telehealth for Cats with Chronic Mobility Challenges** study!
We appreciate your continued interest in our work, and your willingness to participate in our study.

Please answer each question to the best of your ability. If you make a mistake, you can use the back button to return to previous questions. This survey should take 10-15 minutes to complete. 

Thank you!

**Q1:** Please type your cat's name here:_______________________________________________

**Q2:** Please type your email here:___________________________________________________

The following questions will provide us updated information about your cat and their mobility challenges or arthritis.

Please answer each question to the best of your ability. If you make a mistake, you can use the back button to return to previous questions.

**Q3:** Supplements y/n Is your cat currently taking any over-the-counter, or non-prescription supplements (e.g., glucosamine/chondroitin, therapeutic diets) for their chronic mobility challenges or arthritis?

- Yes
- No

**Display This Question:** If Q3 = *Yes*

**Q4:** Please list which of the following supplements you are currently giving your cat to help manage their chronic mobility challenges or arthritis.

|  | Yes | Not currently taking this supplement |
| --- | --- | --- |
| Glucosamine/chondroitin (e.g., Dasuquin or Cosequin) |  |  |
| Omega-3 fatty acids |  |  |
| Cannabidiol (CBD) products |  |  |
| Green-lipped mussel |  |  |
| Joint diet (e.g., Mobility Support) |  |  |
| Other (please list) |  |  |

**Q5:** Is your cat currently receiving or taking any prescription medications (e.g., gabapentin, NSAIDs, Solensia, Adequan) for their chronic mobility challenges or arthritis?

- Yes
- No

**Display This Question:** If Q5 = *Yes*

**Q6:** Please list which of the following medications you are giving your cat to help manage their chronic mobility challenges or arthritis.

|  | Yes | Not currently taking this medication |
| --- | --- | --- |
| Non-steroidal anti-inflammatories (NSAIDS), e.g., meloxicam, Onsior® (robenacoxib) |  |  |
| Gabapentin |  |  |
| Cerenia® (maropitant citrate) |  |  |
| Opioid pain medications (e.g., buprenorphine) |  |  |
| Tramadol |  |  |
| Amantadine |  |  |
| Solensia® (frunevetmab), a monoclonal antibody treatment |  |  |
| Adequan® (Polysulfated glycosaminoglycan) |  |  |
| Other (please list) |  |  |

Q7: Is your cat currently utilizing any non-medication/supplement treatments (e.g., acupuncture, weight management, physical therapy) for their chronic mobility challenges or arthritis?

- Yes
- No

**Display This Question:** If Q7= *Yes*

**Q8:** Please list which of the following treatments you are utilizing or have used since your cat was enrolled in this study (i.e., since you filled out our initial survey in July/August), to help manage their chronic mobility challenges or arthritis.

|  | Yes | Not using/have not used this treatment |
| --- | --- | --- |
| Laser therapy/cold laser |  |  |
| Veterinary acupuncture |  |  |
| Veterinary chiropractic care |  |  |
| Medical massage |  |  |
| Warm/cold compresses |  |  |
| Physical therapy (incl. hydrotherapy) |  |  |
| Stem cell therapy |  |  |
| Platelet-rich-plasma (PRP) |  |  |
| Weight management |  |  |
| Surgery (e.g., femoral head ostectomy or FHO, wrist fusion) |  |  |
| Other (please list) |  |  |

FMPI intro **Feline Musculoskeletal Pain Index - short form**

Please take some time to complete the following questions.
Please mark the circle that best describes your cat's ability to perform the following activities.

*(Copyright 2020 North Carolina State University, reproduced with permission)*

**Q9:** 1. Jump up?

- Normal
- Not quite normal
- Somewhat worse than normal
- Barely, or with great effort
- Not at all

**Q10:** 2. Jump up to kitchen-counter height in one try?

- Normal
- Not quite normal
- Somewhat worse than normal
- Barely, or with great effort
- Not at all

**Q11:** 3. Jump down (how well and how easily)?

- Normal
- Not quite normal
- Somewhat worse than normal
- Barely, or with great effort
- Not at all

**Q12:** 4. Play with toys and/or chase objects?

- Normal
- Not quite normal
- Somewhat worse than normal
- Barely, or with great effort
- Not at all

**Q13:** 5. Play and interact with other pets?

- Normal
- Not quite normal
- Somewhat worse than normal
- Barely, or with great effort
- Not at all

**Q14:** 6. Get up from a resting position?

- Normal
- Not quite normal
- Somewhat worse than normal
- Barely, or with great effort
- Not at all

**Q15:** 7. Lie and/or sit down?

- Normal
- Not quite normal
- Somewhat worse than normal
- Barely, or with great effort
- Not at all

**Q16:** 8. Stretch?

- Normal
- Not quite normal
- Somewhat worse than normal
- Barely, or with great effort
- Not at all

**Q17:** 9. Groom himself or herself?

- Normal
- Not quite normal
- Somewhat worse than normal
- Barely, or with great effort
- Not at all

The following questions will focus on your experiences with and opinions on, using **in-clinic** veterinary appointments for your cat's chronic mobility challenges or arthritis.

Please answer each question to the best of your ability. If you make a mistake, you can use the back button to return to previous questions.

**Q18:** How do you feel about veterinary appointments for your cat's chronic mobility challenges or arthritis?

- It is very important for my cat to see a veterinarian for this
- It is somewhat important for my cat to see a veterinarian for this
- It is neither important nor unimportant for my cat to see a veterinarian for this
- It is somewhat unimportant for my cat to see a veterinarian for this
- It is very unimportant for my cat to see a veterinarian for this

**Q19:** Do any of the following **human-related factors** prevent you from accessing veterinary care for your cat's chronic mobility challenges or arthritis?
*(Please move the slider to the number on the slide that is most accurate.)*

|  |  | Not at all | Very little | Somewhat | A moderate amount | A large amount |
| --- | --- | --- | --- | --- | --- | --- |

|  | 0 | 1 | 2 | 3 | 4 | 5 |
| --- | --- | --- | --- | --- | --- | --- |

| Not sure where to get veterinary care | 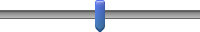 |
| --- | --- |
| Cost of veterinary care | 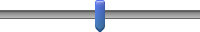 |
| Finding appointments that work with my schedule | 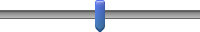 |
| Distance from/to preferred veterinary clinic | 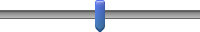 |
| Transportation challenges (e.g., vehicle availability, human mobility/injury, etc.) | 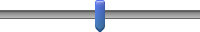 |
| Living with a disability or chronic health condition | 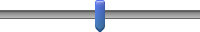 |
| My stress level | 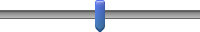 |
| My past experiences with clinic visits | 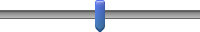 |

**Q20:** Do any of the following **cat-related factors** prevent you from accessing veterinary care for your cat's chronic mobility challenges or arthritis?
*(Please move the slider to the number on the slide that is most accurate.)*

|  |  | Not at all | Very little | Somewhat | A moderate amount | A large amount | Not Applicable |
| --- | --- | --- | --- | --- | --- | --- | --- |

|  | 0 | 1 | 2 | 3 | 4 | 5 |
| --- | --- | --- | --- | --- | --- | --- |

| Finding my cat for their appointment | 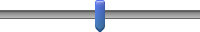 |
| --- | --- |
| Access to a carrier or leash for my cat | 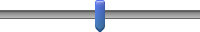 |
| Getting my cat into the carrier or leash | 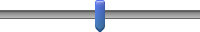 |
| Cat behavior during travel to the clinic | 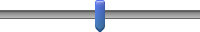 |
| Cat's stress level | 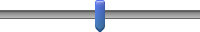 |
| Cat's past experiences with clinic visits | 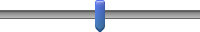 |

The following questions will focus on your experiences with and opinions on, **video conferencing software and telehealth** appointments.

Please answer each question to the best of your ability. If you make a mistake, you can use the back button to return to previous questions.

**Q21:** Have you ever used a video conferencing platform (e.g., Zoom, Facetime, Skype, Microsoft Teams, Google Meet) before?

- No
- Yes

**Q22:** How comfortable are you with using video conferencing platforms (e.g., Zoom, Facetime, Skype, Microsoft Teams, Google Meet)?

- Very comfortable
- Somewhat comfortable
- Neutral
- Somewhat uncomfortable
- Very uncomfortable

**For the following questions, please keep this definition in mind:** Telehealth is the overarching term that encompasses all uses of technology to remotely gather and deliver health information, advice, education, and care.

**Q23:** Have you ever had a **video telehealth** visit for a pet?

In other words, have you ever used video conferencing software such as Zoom, Facetime, Skype, Microsoft Teams, Google Meet, with your veterinarian?
*(Please select all that apply)*

- Yes, for your cat
- Yes, for a pet other than your cat
- No

**Q24:** Have you ever had a **video telehealth** visit for yourself?

In other words, have you ever used video conferencing software such as, but not limited to, Zoom, Facetime, Skype, Microsoft Teams, Google Meet, with a healthcare provider (i.e. physician, nurse, dermatologist, etc.)?

- Yes
- No

**Q25:** Please rate your ability to access the following, for a veterinary video telehealth appointment:

|  | Very easy | Somewhat easy | Neither easy nor difficult | Somewhat difficult | Very difficult |
| --- | --- | --- | --- | --- | --- |
| Accessing the necessary **technology** (e.g., computer, tablet, smartphone, camera/webcam) for an appointment is: |  |  |  |  |  |
| Ensuring **strong,** **reliable internet connection** for an appointment is: |  |  |  |  |  |
| Accessing and using the **websites/apps** needed for an appointment is: |  |  |  |  |  |

**Q26:** If veterinary **video telehealth** were available to you, please rate your interest in using it to help with at-home management of your cat's chronic mobility challenges or arthritis?

- Very interested
- Somewhat interested
- Neutral
- Somewhat uninterested
- Very uninterested

**Q27:** In comparison to what you would expect to pay for a normal in-clinic veterinary recheck appointment (to help with chronic mobility challenges or arthritis management for your cat), how much are you willing to pay for a video telehealth recheck?

- I would not be willing to pay for this service
- Much less
- A little less
- About the same
- A little more
- Much more

The following questions will focus on your **at-home experiences** with your cat.

Please answer each question to the best of your ability. If you make a mistake, you can use the back button to return to previous questions.

**Q28:** Overall, how confident do you feel in your knowledge about your cat's chronic mobility challenges or arthritis needs?

- Very confident
- Somewhat confident
- Neutral
- Somewhat unconfident
- Very unconfident

**Q29:** How supported do you feel by your veterinarian and veterinary staff in your efforts to care for your cat's chronic mobility challenges or arthritis?

- Very unsupported
- Somewhat unsupported
- Neutral
- Somewhat supported
- Very supported
- Not applicable

**Q30:** How helpful do you feel your **current** management strategies are for keeping your cat comfortable at home?

- Very helpful
- Somewhat helpful
- Neutral
- Somewhat unhelpful
- Very unhelpful
- Not applicable/no strategies implemented

**Display This Question:** If Q5 = *Yes* **Or** Q7 = *Yes*

**Q31:** How easy or difficult is it for you to give your cat's medications or supplements by mouth (pills/tablets, liquids)?
*Note: This does not include medications mixed in with food.*

|  | Very Difficult | Somewhat difficult | Neutral | Somewhat easy | Very easy | Not applicable |
| --- | --- | --- | --- | --- | --- | --- |
| Oral tablets/pills |  |  |  |  |  |  |
| Oral liquids |  |  |  |  |  |  |

**Q32:** Which would you prefer, video telehealth or in-clinic, for addressing the following aspects of your cat's chronic mobility challenges or arthritis:

|  | Video telehealth | In-clinic | No preference |
| --- | --- | --- | --- |
| Cat's stress level |  |  |  |
| My stress level |  |  |  |
| Physical examinations |  |  |  |
| Diagnosing my cat's mobility challenges/arthritis |  |  |  |
| Developing treatment plans for my cat's mobility challenges/arthritis |  |  |  |
| Providing guidance on giving prescribed medications to my cat |  |  |  |
| Helping me feel supported & prepared to meet my cat's needs |  |  |  |
| Education about my cat's mobility challenges/arthritis |  |  |  |
| Implementing environmental changes my veterinarian has recommended for my cat |  |  |  |
| Rechecks for my cat's mobility challenges/arthritis |  |  |  |

**Q33:** Would you like to receive the 20-30 minute educational presentation on home-management for cats with chronic mobility challenges or arthritis?

If you select yes, we will email you to schedule a time for this presentation, which will be given by one of our researchers.

- Yes
- No

**Q34**: Is there anything else you would like to add about your experiences with in-clinic or video telehealth for managing your cat's chronic mobility challenges or arthritis?

________________________________________________________________

________________________________________________________________

________________________________________________________________

________________________________________________________________

________________________________________________________________

**S.1.5** Participant US state/territory or Canadian province of residence (N=106).

Only those states/provinces where at least one participant indicated residing, are listed.

| **Category** | **Variable** | **Percentage** |
| --- | --- | --- |
| US State of Residence (n = 95) | Arizona | 4.2 |
|  | California | 29.5 |
|  | Florida | 2.1 |
|  | Georgia | 4.2 |
|  | Idaho | 2.1 |
|  | Illinois | 2.1 |
|  | Indiana | 1.1 |
|  | Iowa | 1.1 |
|  | Kansas | 2.1 |
|  | Maine | 1.1 |
|  | Maryland | 3.2 |
|  | Massachusetts | 1.1 |
|  | Michigan | 2.1 |
|  | Minnesota | 1.1 |
|  | Nevada | 1.1 |
|  | New Hampshire | 1.1 |
|  | New Jersey | 3.2 |
|  | New York | 2.1 |
|  | North Carolina | 3.2 |
|  | Ohio | 3.2 |
|  | Oregon | 6.3 |
|  | Pennsylvania | 2.1 |
|  | South Dakota | 1.1 |
|  | Tennessee | 2.1 |
|  | Texas | 4.2 |
|  | US Virgin Islands | 1.1 |
|  | Utah | 1.1 |
|  | Virgina | 3.2 |
|  | Washington | 5.3 |
|  | West Virginia | 1.1 |
|  | Wisconsin | 1.1 |
|  | Wyoming | 1.1 |
| Canadian Province (n = 11) | British Columbia | 36.4 |
|  | Manitoba | 9.1 |
|  | Nova Scotia | 36.4 |
|  | Ontario | 18.2 |

**S.1.6** Percentage of participants indicating they utilize supplements, medications, and other interventions for their cat’s chronic mobility problems (initial and final surveys; N=106), and the length of time (<3m, 3-6m, 7-12m, >1yr) their cat had been receiving these interventions (initial survey only; N=106).

| **Category** | **Variable** | **Initial Survey Responses** | | | | | | **Final Survey Responses** | | |  |
| --- | --- | --- | --- | --- | --- | --- | --- | --- | --- | --- | --- |
|  |  | No  % | Yes  % | <3 m  % | 3-6m  % | 7-12m  % | >1yr  % | No  % | Yes % | | |
| **Supplements** | Overall | 77.4 | 22.6 |  |  |  |  | 76.4 | 23.6 | | |
|  | Glucosamine | 25.0 | 75.0 | 8.3 | 4.2 | 12.5 | 50.0 | 36.0 | | 64.0 |  |
|  | Omega 3 FAs | 62.5 | 37.5 | 4.2 | 0.0 | 4.2 | 29.2 | 68.0 | | 32.0 |  |
|  | Cannabidiol | 83.3 | 16.7 | 4.2 | 4.2 | 0.0 | 8.3 | 92.0 | | 8.0 |  |
|  | Green lipped mussel | 83.3 | 16.7 | 4.2 | 0.0 | 4.2 | 8.3 | 92.0 | | 8.0 |  |
|  | Joint diet | 87.5 | 12.5 | 4.2 | 0.0 | 4.2 | 4.2 | 92.0 | | 8.0 |  |
|  | Other | 66.7 | 33.3 | 8.3 | 4.2 | 12.5 | 8.3 | 68.0 | | 32.0 |  |
| **Medications** | Overall | 47.2 | 52.8 |  |  |  |  | 46.2 | | 53.8 |  |
|  | NSAIDs | 87.5 | 12.5 | 3.6 | 1.8 | 1.8 | 5.4 | 89.5 | | 10.5 |  |
|  | Gabapentin | 64.3 | 35.7 | 10.7 | 3.6 | 8.9 | 12.5 | 70.2 | | 29.8 |  |
|  | Cerenia® | 91.1 | 8.9 | 0.0 | 0.0 | 0.0 | 8.9 | 91.2 | | 8.8 |  |
|  | Opioids | 96.4 | 3.6 | 0.0 | 0.0 | 0.0 | 3.6 | 96.5 | | 3.5 |  |
|  | Tramadol | 100.0 | 0.0 | 0.0 | 0.0 | 0.0 | 0.0 | 100.0 | | 0.0 |  |
|  | Amantadine | 100.0 | 0.0 | 0.0 | 0.0 | 0.0 | 0.0 | 100.0 | | 0.0 |  |
|  | Solensia® | 28.6 | 71.4 | 17.9 | 25.0 | 25.0 | 3.6 | 31.6 | | 68.4 |  |
|  | Adequan® | 83.9 | 16.1 | 3.6 | 3.6 | 3.6 | 5.4 | 86.0 | | 14.0 |  |
|  | Other | 85.7 | 14.3 | 1.8 | 5.4 | 5.4 | 1.8 | 86.0 | | 14.0 |  |
| **Other treatments** | Overall | 80.2 | 19.8 |  |  |  |  | 81.1 | | 18.9 |  |
|  | Cold Laser | 81.0 | 19.0 | 9.5 | 0.0 | 0. | 9.5 | 85.0 | | 15.0 |  |
|  | Acupuncture | 71.4 | 28.6 | 4.8 | 9.5 | 14.3 | 0.0 | 75.0 | | 25.0 |  |
|  | Chiropractic | 95.2 | 4.8 | 0.0 | 0.0 | 4.8 | 0.0 | 95.0 | | 5.0 |  |
|  | Massage | 100.0 | 0.0 | 0.0 | 0.0 | 0.0 | 0.0 | 90.0 | | 10.0 |  |
|  | Warm/cold compress | 81.0 | 19.0 | 0.0 | 4.8 | 0.0 | 14.3 | 80.0 | | 20.0 |  |
|  | Physical therapy | 90.5 | 9.5 | 0.0 | 4.8 | 0.0 | 4.8 | 90.0 | | 10.0 |  |
|  | Stem cell therapy | 100.0 | 0.0 | 0.0 | 0.0 | 0.0 | 0.0 | 100.0 | | 0.0 |  |
|  | Platelet-rich-plasma | 100.0 | 0.0 | 0.0 | 0.0 | 0.0 | 0.0 | 100.0 | | 0.0 |  |
|  | Weight management | 23.8 | 76.2 | 4.8 | 19.0 | 4.8 | 47.6 | 30.0 | | 70.0 |  |
|  | Surgery | 90.5 | 9.5 | 0.0 | 0.0 | 0.0 | 9.5 | 100.0 | | 0.0 |  |
|  | Other | 85.7 | 14.3 | 4.8 | 0.0 | 4.8 | 4.8 | 80.0 | | 20.0 |  |
