# Supplementary material for: Incorporating video telehealth for improving at-home management of chronic health conditions in cats: a focus on chronic mobility problems
Source: Front Vet Sci. 2025 Apr 1;12:1510006. doi: 10.3389/fvets.2025.1510006 (PMC11997976; doi:10.3389/fvets.2025.1510006)
Supplement: Supplementary file 2 [file Supplementary_file_2.pdf]

Home Management of Cats with Chronic Mobility Challenges

Grace Boone[1], Daniel Pang[2], Hao-Yu Shih[3], Carly Moody[1]

[1] University of California, Davis; Davis, California
[2] University of Calgary; Calgary, Alberta, Canada
[3] Mayo Clinic; Rochester, MN

https://animalscience.ucdavis.edu/people/faculty/carly-moody

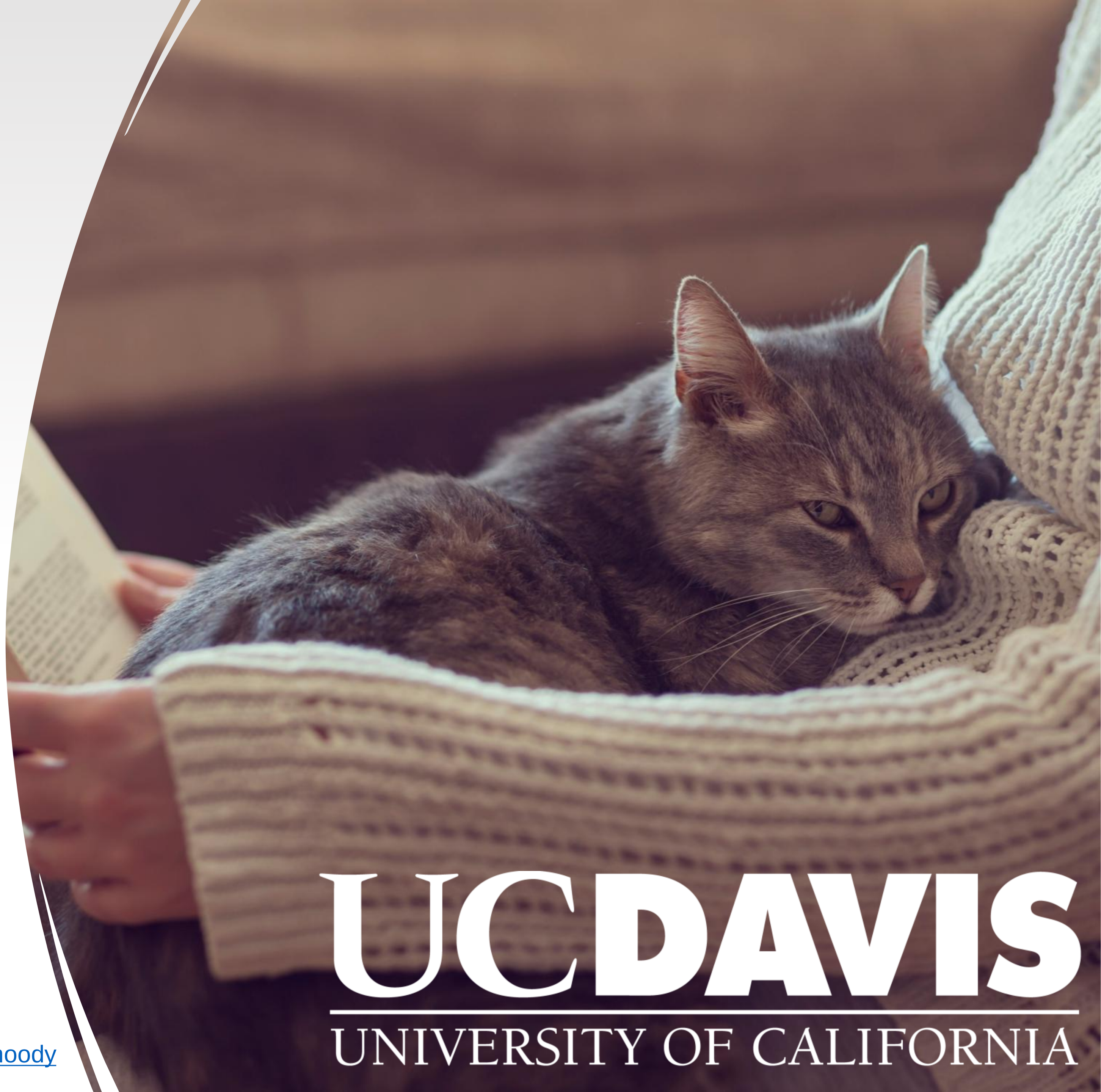

Outline

- Overview of chronic mobility challenges & arthritis
- At-home management strategies
- Tips on giving medications
- When to seek veterinary care or advice

(Boone et al., 2025)

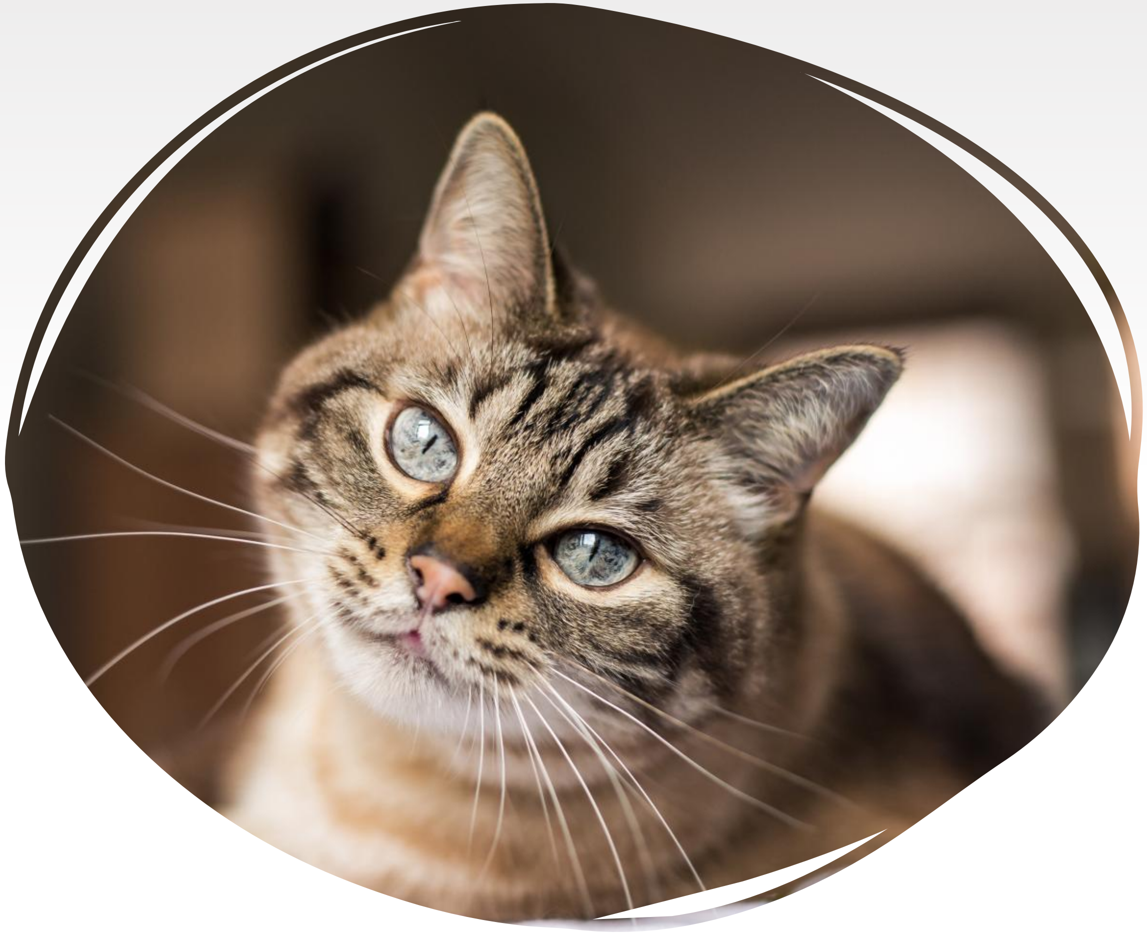

Overview of Chronic Mobility Challenges/Arthritis

(Boone et al., 2025)

Terms & Definitions

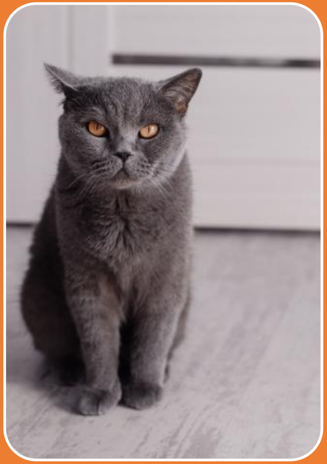

Chronic mobility challenges

Difficulty standing, walking, jumping, using stairs, getting up, going in/out of litter box, grooming. Chronic = more than 3 months?[3]

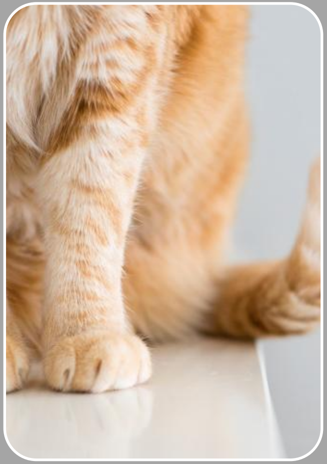

Arthritis

Any condition causing inflammation in the joints, either due to changes in joint tissues or deterioration of cartilage.[1,2]

- Osteoarthritis (OA)
- Degenerative Joint Disease (DJD)

1 American College of Veterinary Surgeons
2 Williams & Yuill
3 American Animal Hospital Association

(Boone et al., 2025)

UC DAVIS

Key Facts

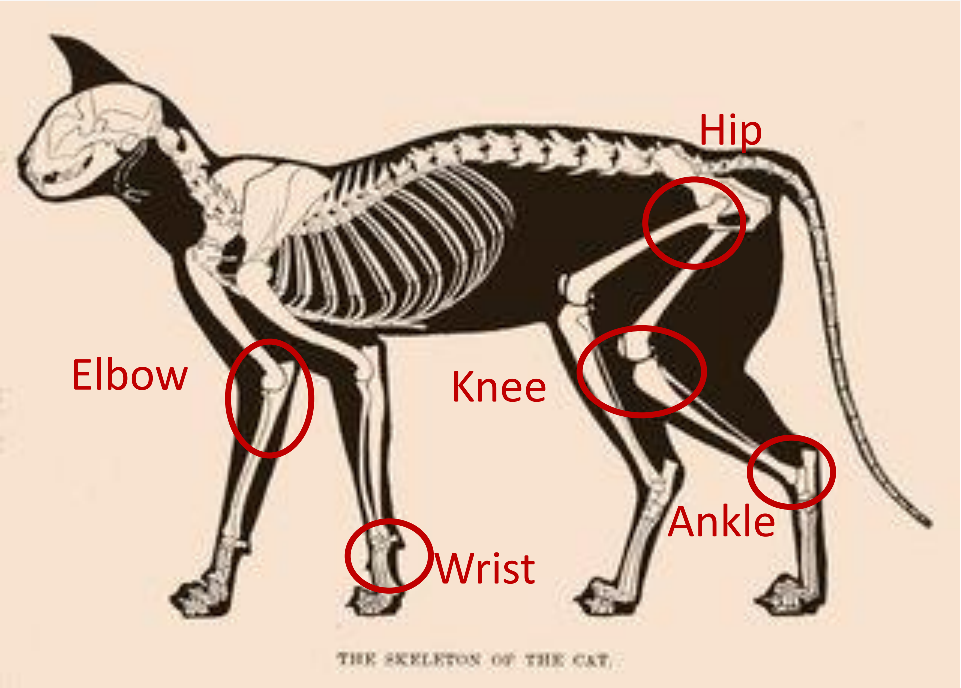

This Photo by Unknown Author is licensed under CC BY-SA

Cats of all ages can have arthritis & it may go undetected[4,5]

- Older cats more likely to be clinically affected
- Cats younger than 5 can be affected!

Joints often affected in cats[1]

- Hip
- Knee
- Ankle
- Elbow
- Wrist

OA underdiagnosed in cats[1]

- Increased awareness → more frequent diagnosis & better care

1 American College of Veterinary Surgeons
4 Lascelles, 2010
5 Lascelles et al, 2010

(Boone et al., 2025)

UC DAVIS

Animal Welfare Epidemiology Lab · UC DAVIS

Behavior Changes

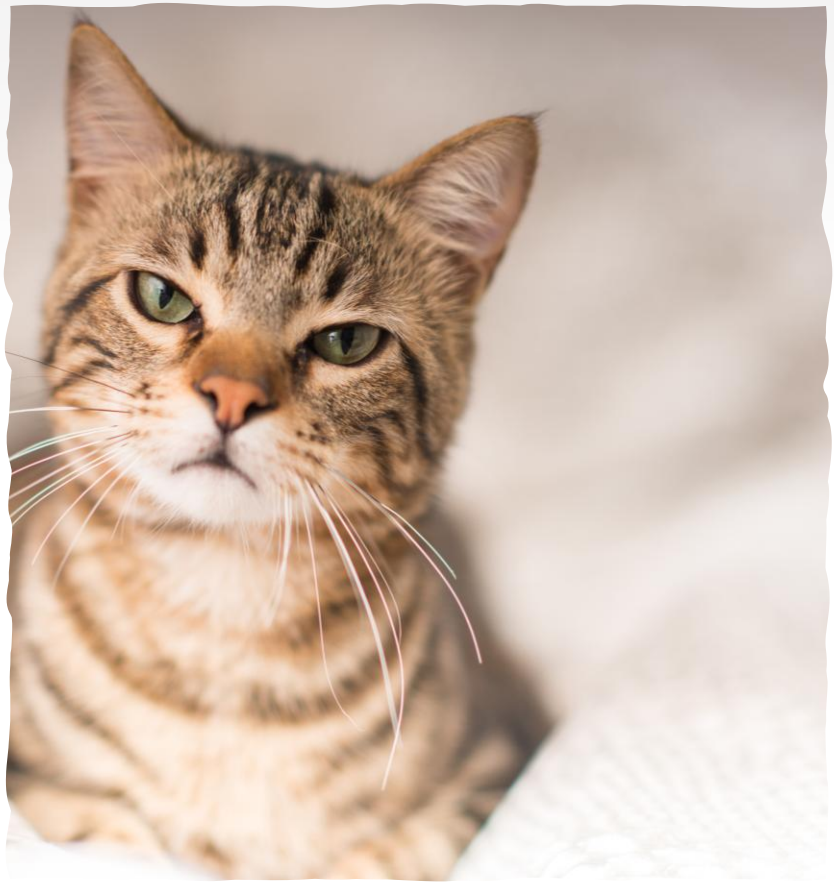

Reduced mobility
- ↓ Jumping
- ↓ Stairs
- ↑ Stiffness/lameness
- ↓ Litter box

Altered grooming
- ↓ Time
- ↑ Matted/scruffy coat
- ↑ Painful joints
- ↑ Overgrown claws

Reduced activity
- ↑ Sleeping/resting
- ↓ Hunting/exploring
- ↓ Interaction & play

Temperament changes
- ↑ Irritable/grumpy
- ↓ People, animals
- ↑ Alone time

 International Cat Care, 2018

(Boone et al., 2025)

UC DAVIS

At-home Management Strategies

- Environmental Modifications
- Human-animal interaction modifications
- Interactions with other pets in the home
- Tips on giving medications

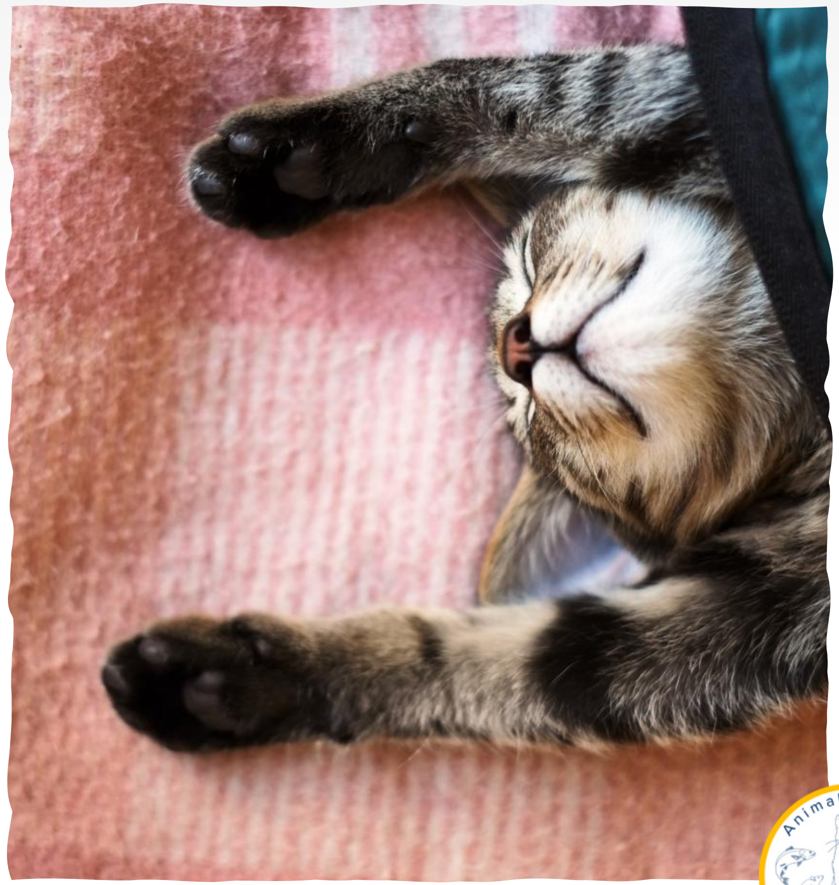

(Boone et al., 2025)

UC DAVIS

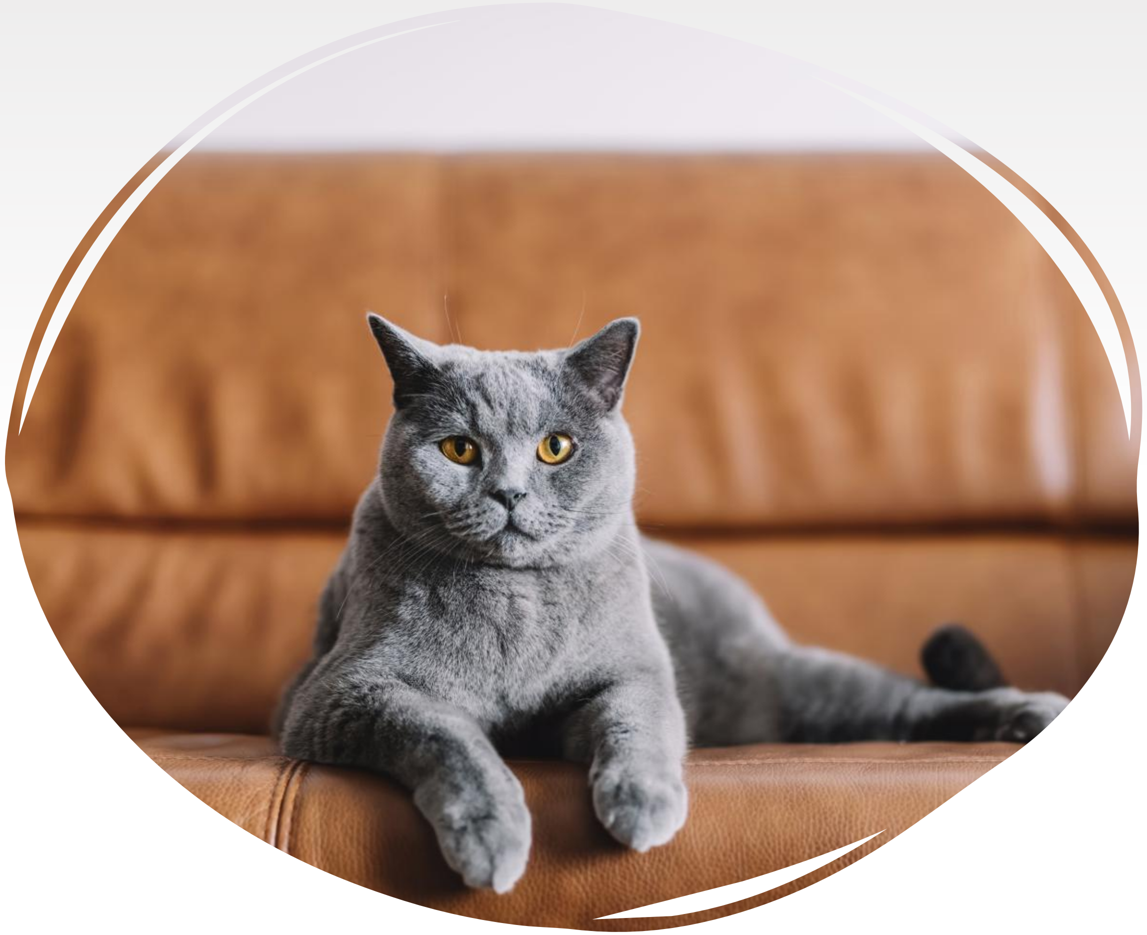

Environmental Modifications

(Boone et al., 2025)

Food & Water

- Water on main level OR each level of the home
- Raised food & water bowls

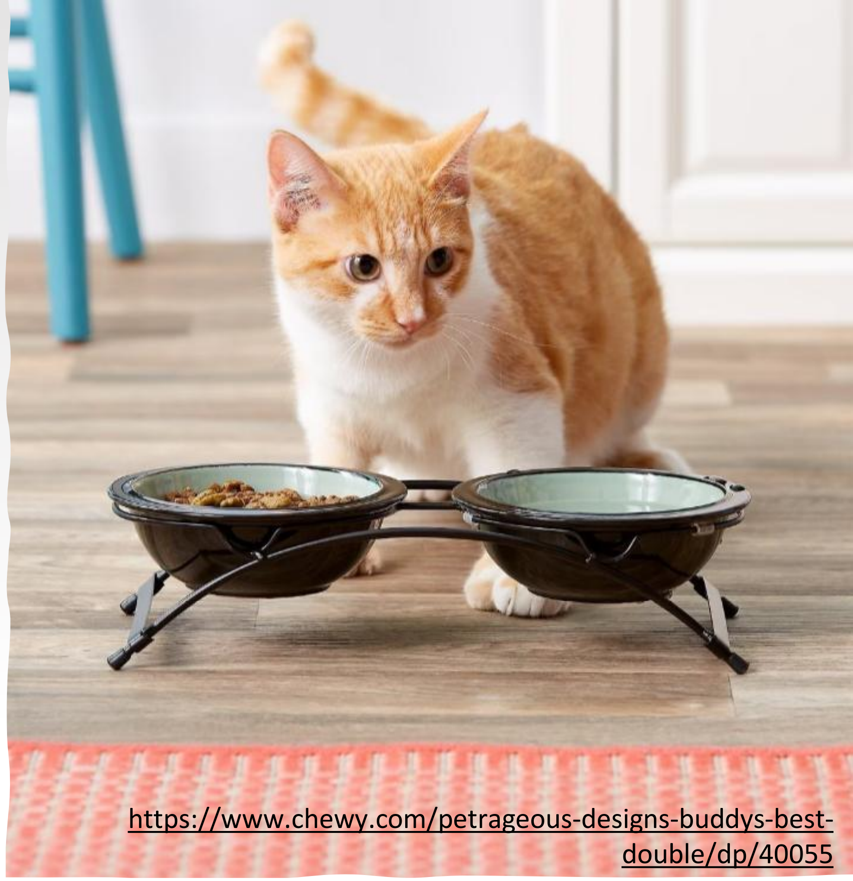

https://www.chewy.com/petrageous-designs-buddys-best-double/dp/40055

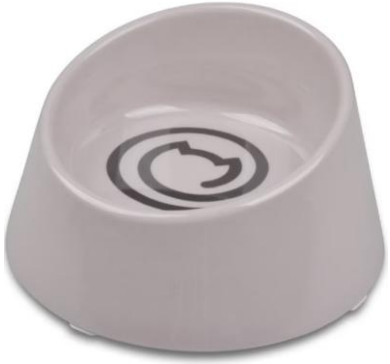

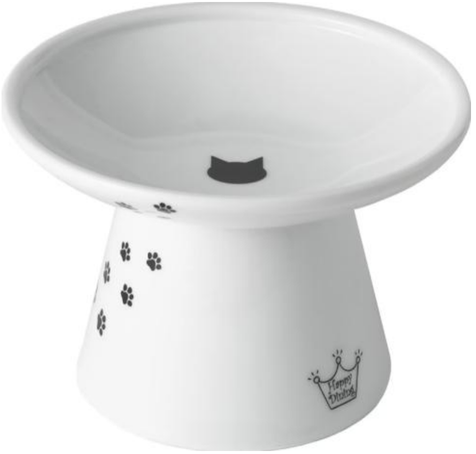

https://www.chewy.com/necoichi-ceramic-elevated-dog-cat/dp/146186

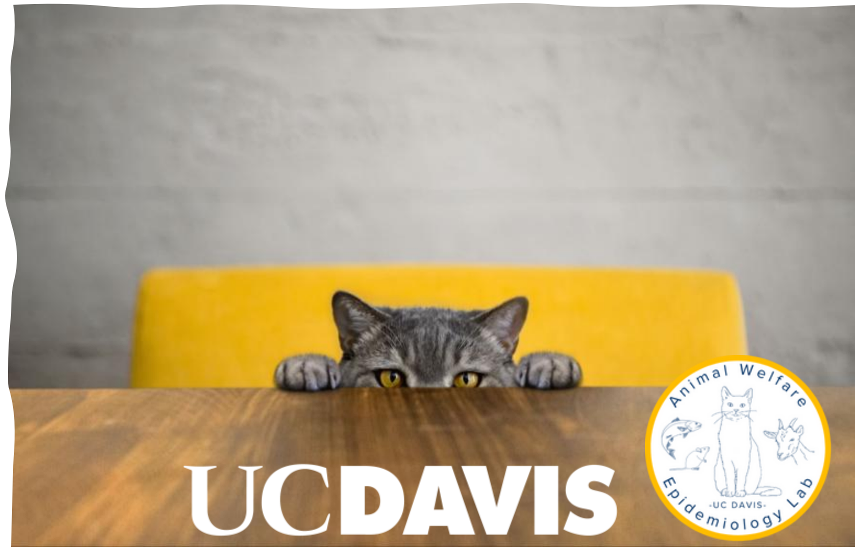

(Boone et al., 2025)

https://www.chewy.com/van-ness-ecoware-raised-cat-bowl-cool/dp/756846

UCDAVIS

Litter Boxes

- On main level OR on each level of the home
- Multiple locations
- Low-traffic & low-noise area
- Low sides/cut an entrance
- Extra large size

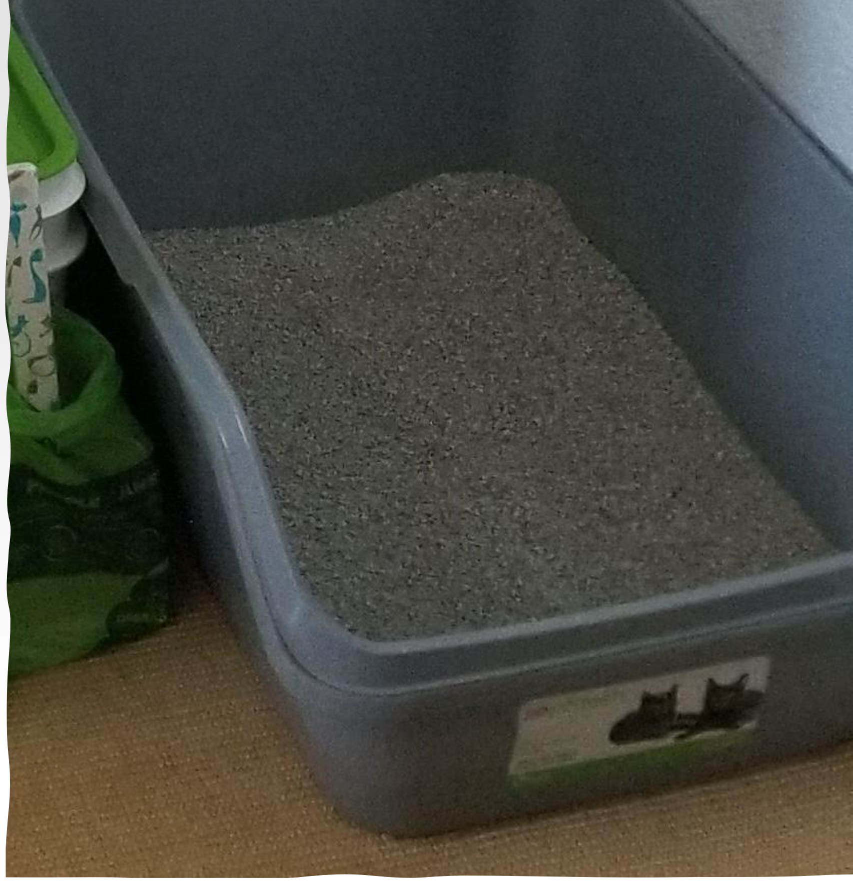

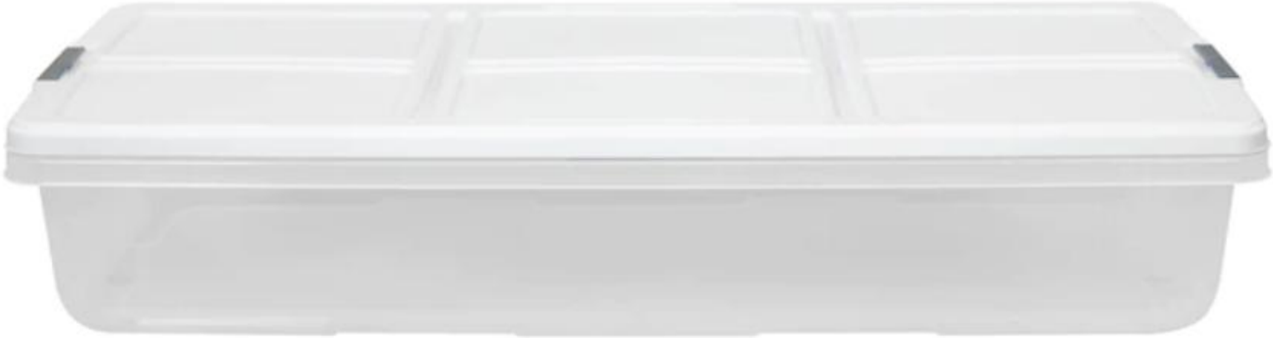

UC DAVIS (Boone et al., 2025)

https://www.lowes.com/pd/Hefty-13-Gallon-52-Quart-Clear-Underbed-Tote-with-Latching-Lid/1000505517

Grooming & Resting

- Grooming
  - Assist cat with keeping clean & tidy
  - Cat-safe wipes/shampoos
- Resting areas
  - Heating mats/pads made for pets
    - OR warm bedding in dryer
  - Soft bedding
  - Floor beds

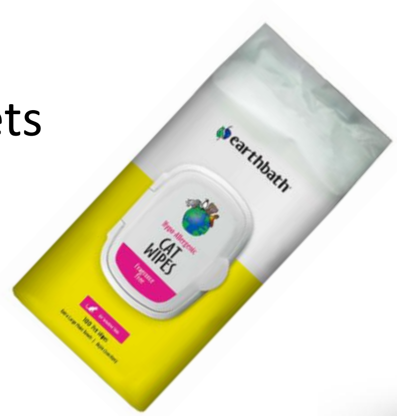

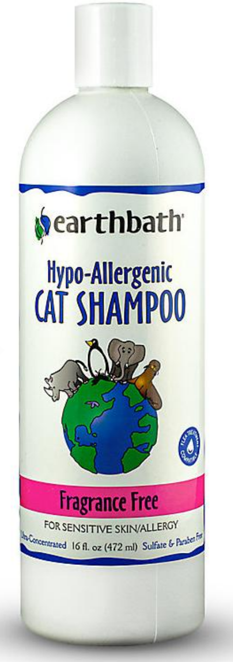

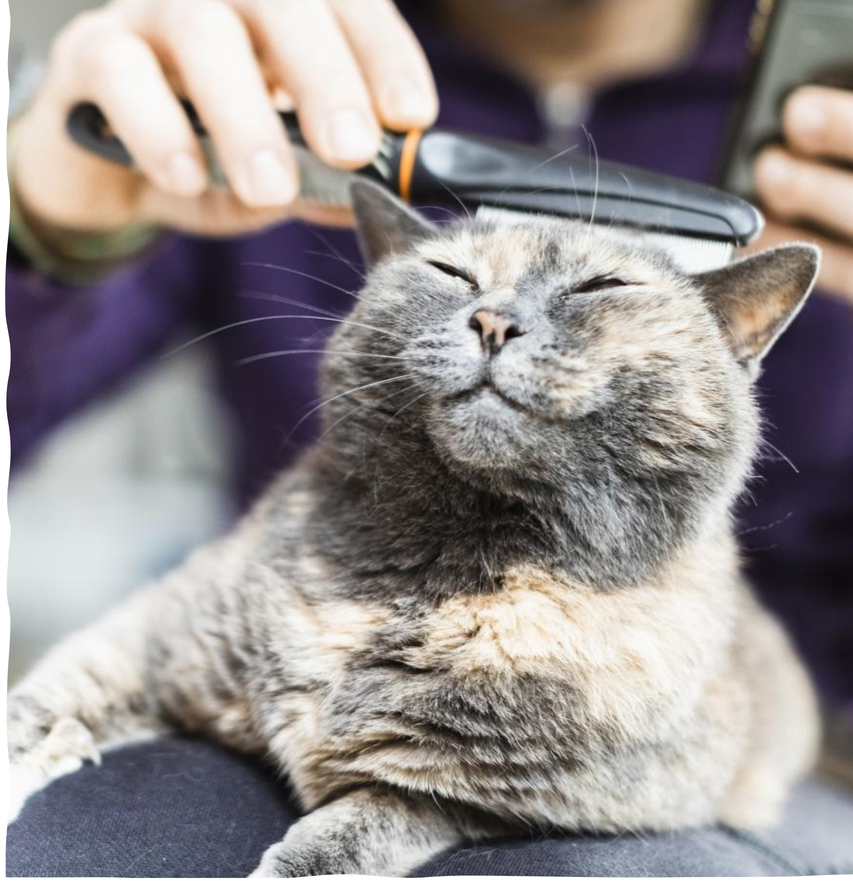

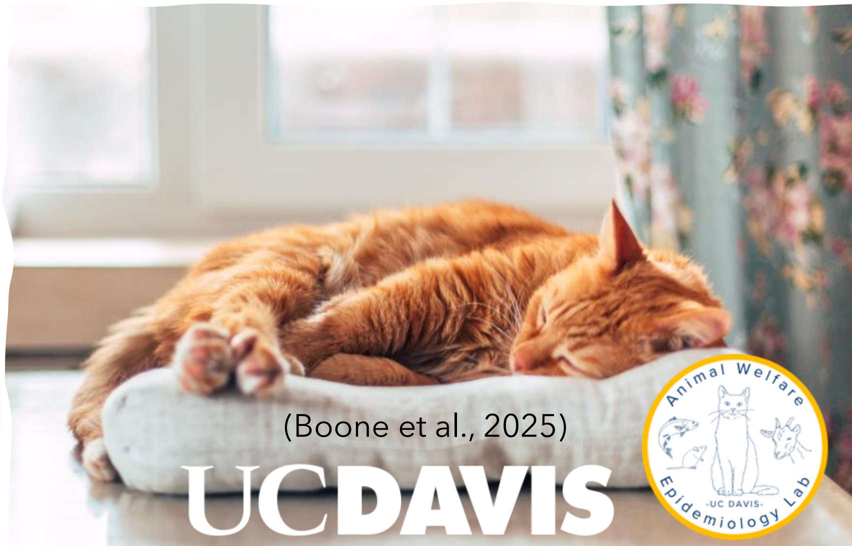

(Boone et al., 2025)

https://www.petsmart.com/cat/grooming-supplies/shampoos-and-conditioners/earthbath-hypo-allergenic-cat-shampoo---fragrance-free-50015.html
https://www.petsmart.com/cat/grooming-supplies/wipes-and-deodorizers/earthbath-hypo-allergenic-cat-wipes---fragrance-free---sensitive-skin---xl-towels---100ct-62176.html

UC DAVIS

Animal Welfare Epidemiology Lab · UC DAVIS

Play

- Keeping cat active is important
- Modify play to suit them
  - Less jumping/lower to the ground
  - Slower movements
  - Shorter sessions
- Puzzle toys

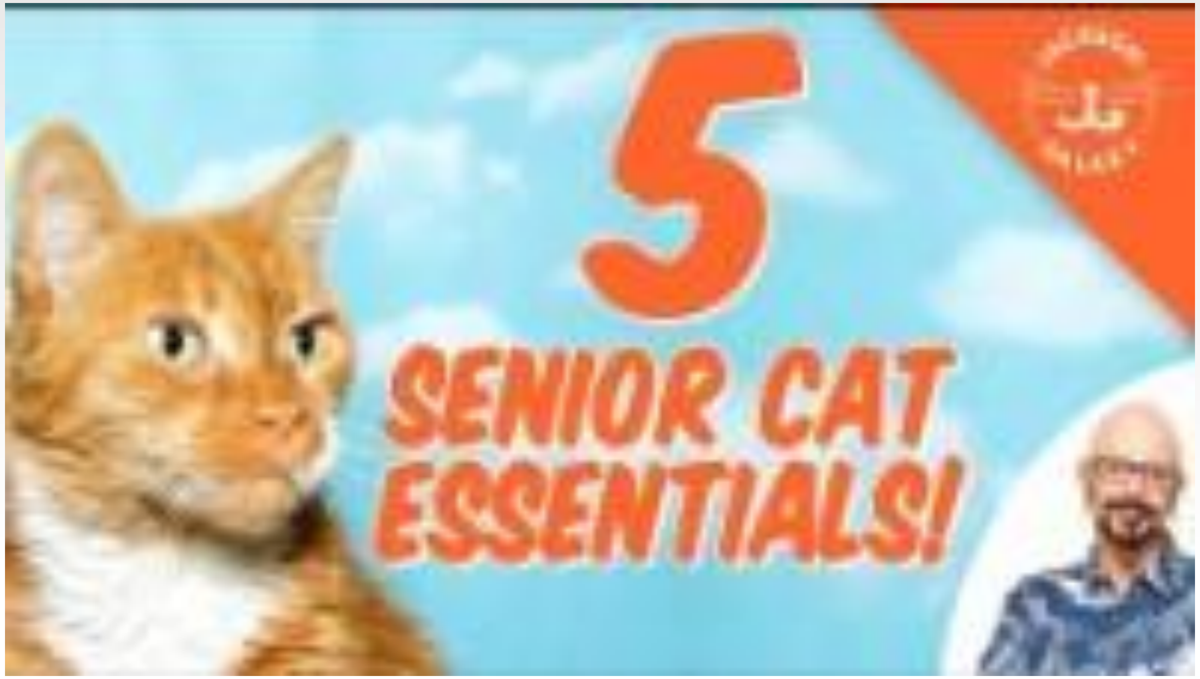

https://www.youtube.com/watch?v=0XPRz8ZUkck&t=658s

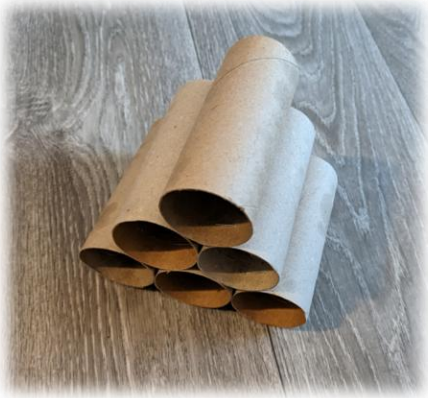

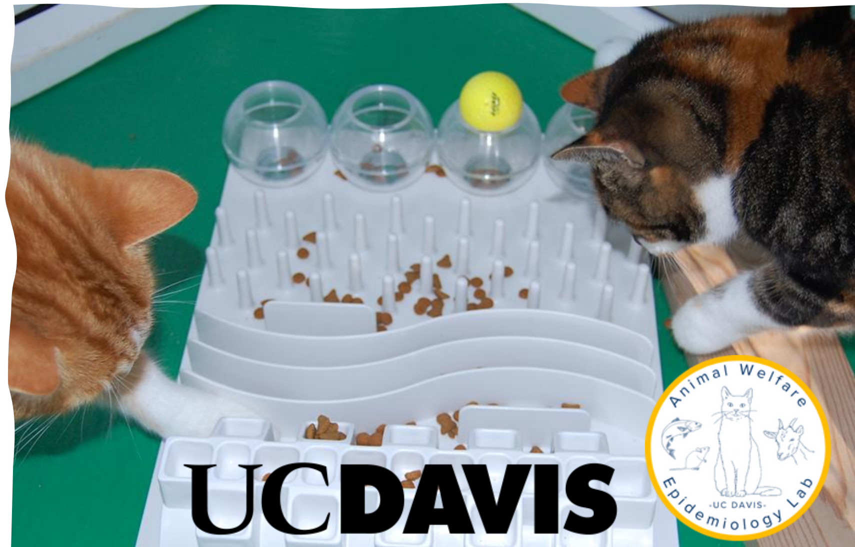

http://foodpuzzlesforcats.com/
https://icatcare.org/advice/puzzle-feeders/

(Boone et al., 2025)

UCDAVIS

Around the House

- Accessing beds, couches, high areas
  - Ramps/stairs
- Mats, rugs
  - Non-slip back to prevent sliding
- Routine, stable environment
  - Memory issues, ↓ stress/anxiety

(Boone et al., 2025)

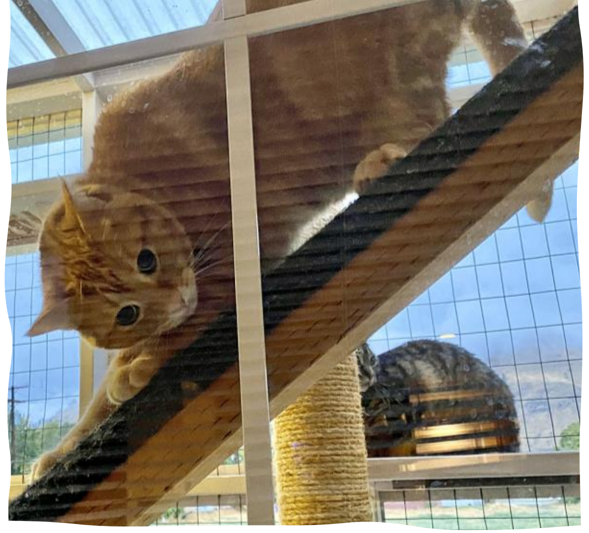

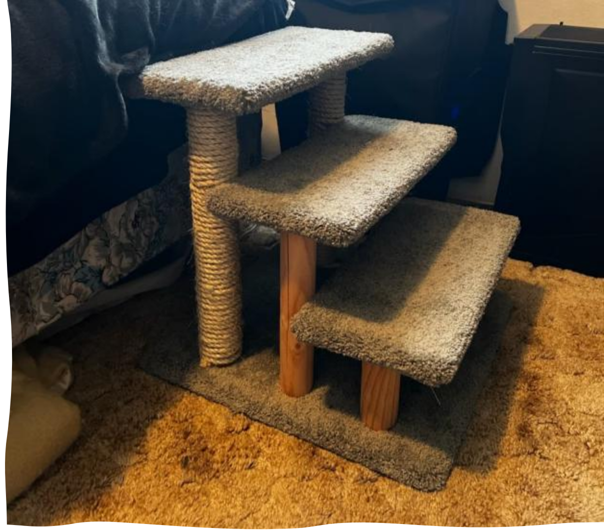

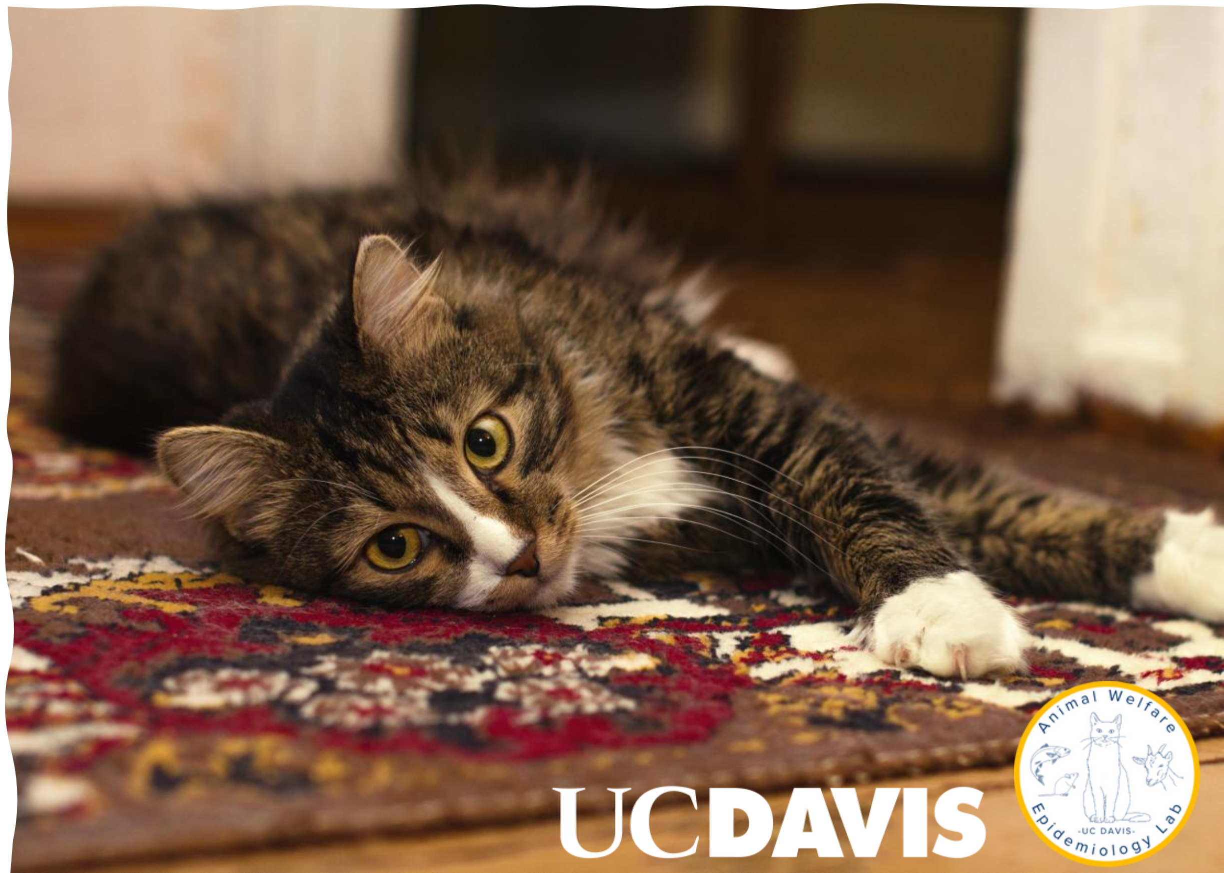

UC DAVIS

Animal Welfare Epidemiology Lab · UC DAVIS

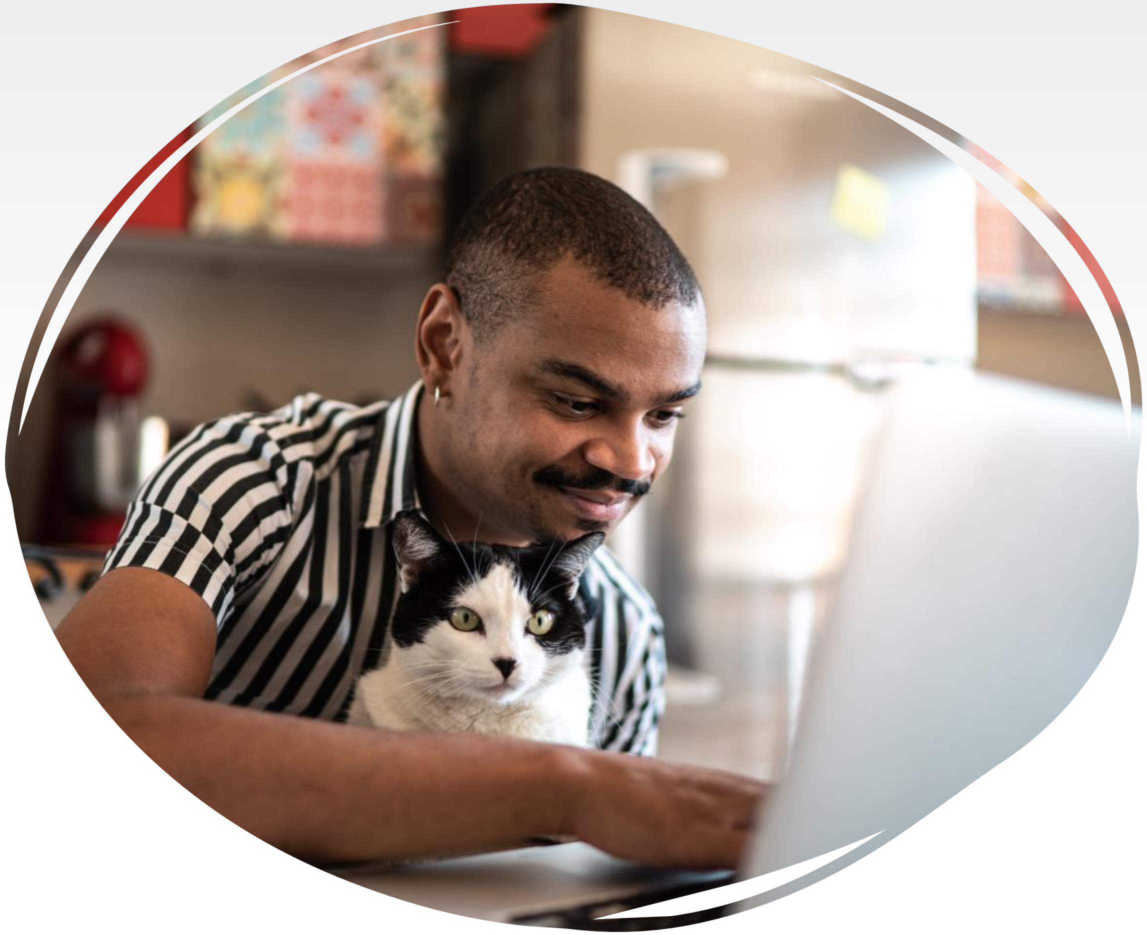

Human-Animal Interactions

(Boone et al., 2025)

Signs of Negative Arousal/Stress

- Ears to the side/back
- Dilated pupils
- Increased breathing/respiration rate
- Struggling/avoidance behavior
- Vocalizing
- Lip licking
  (no food or grooming reason)

Respect your cat's choices!

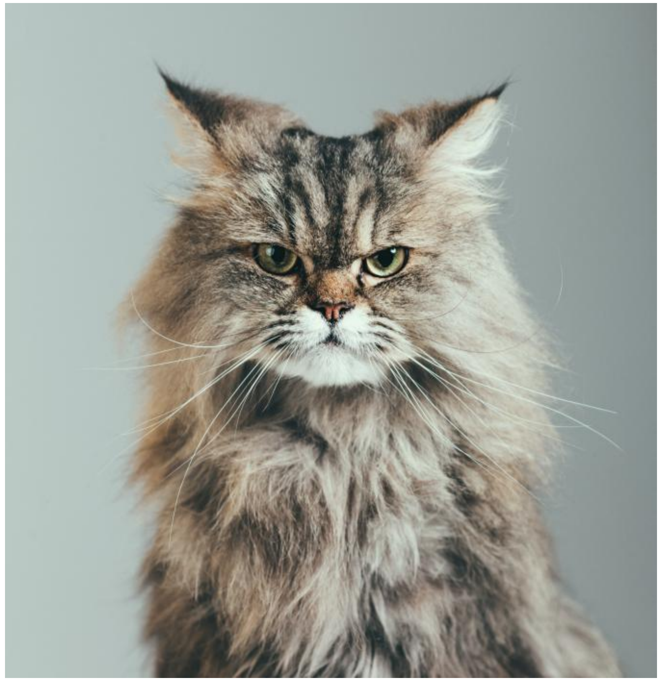

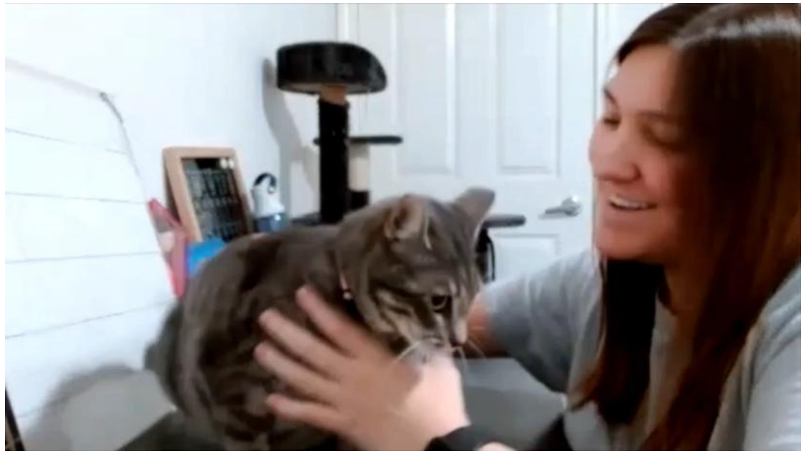

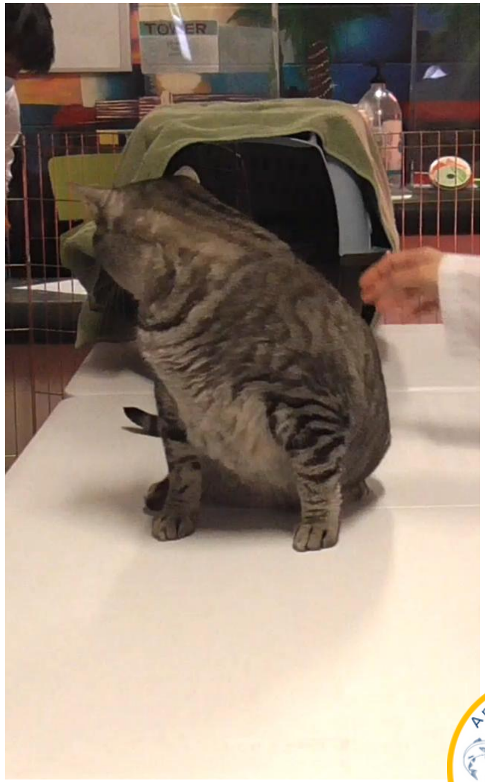

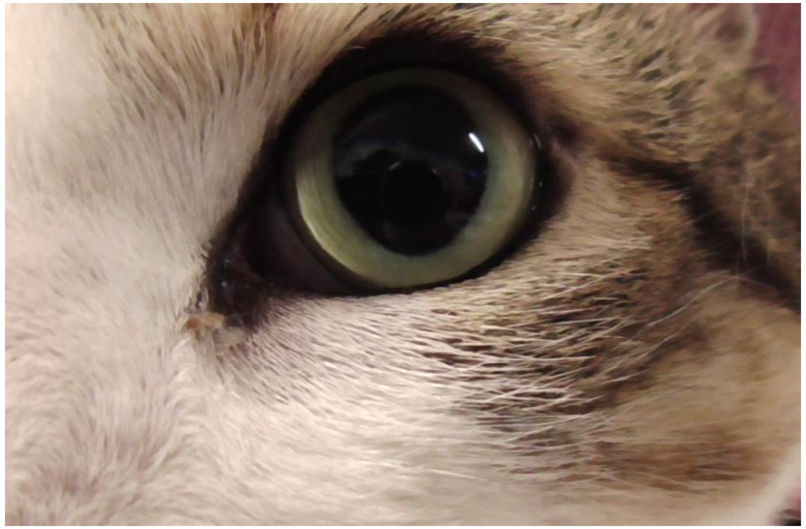

(Boone et al., 2025)

Picking up & Petting

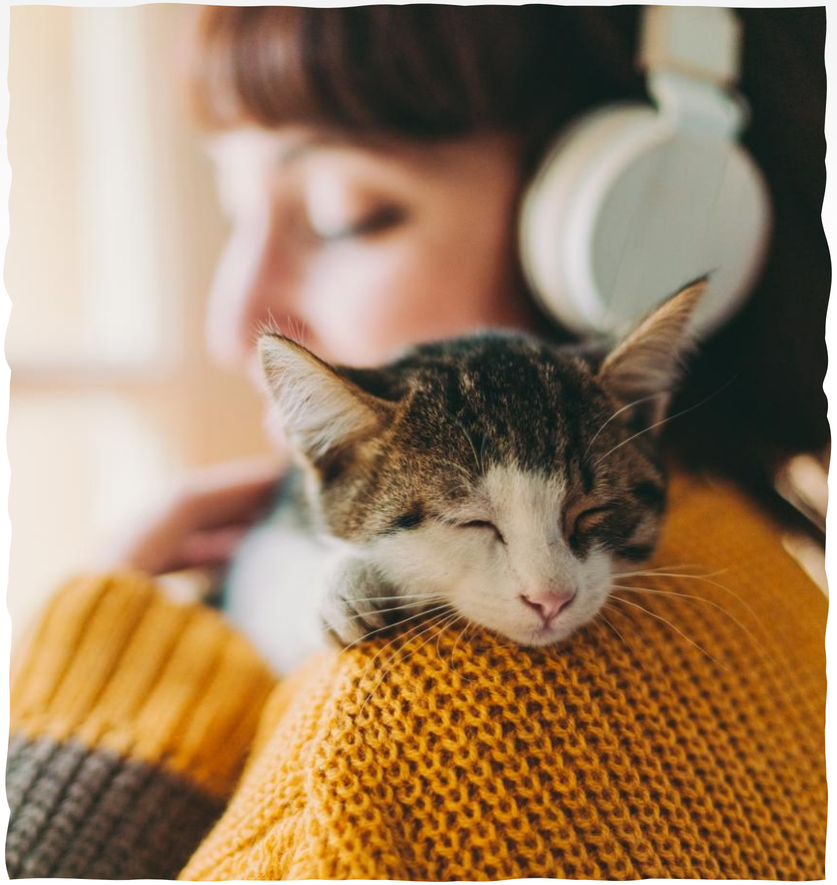

- Minimize picking up if cat seems to dislike
  - Support the whole body
  - Avoid painful areas

- Be gentle when petting
  - Go slow
  - Use a light touch
  - Avoid painful areas
    - Cats tend to prefer the area between the eyes and ears, and around the lips, chin, and cheeks[7]

[7] Ellis et al., 2015

(Boone et al., 2025)

UCDAVIS

Kids

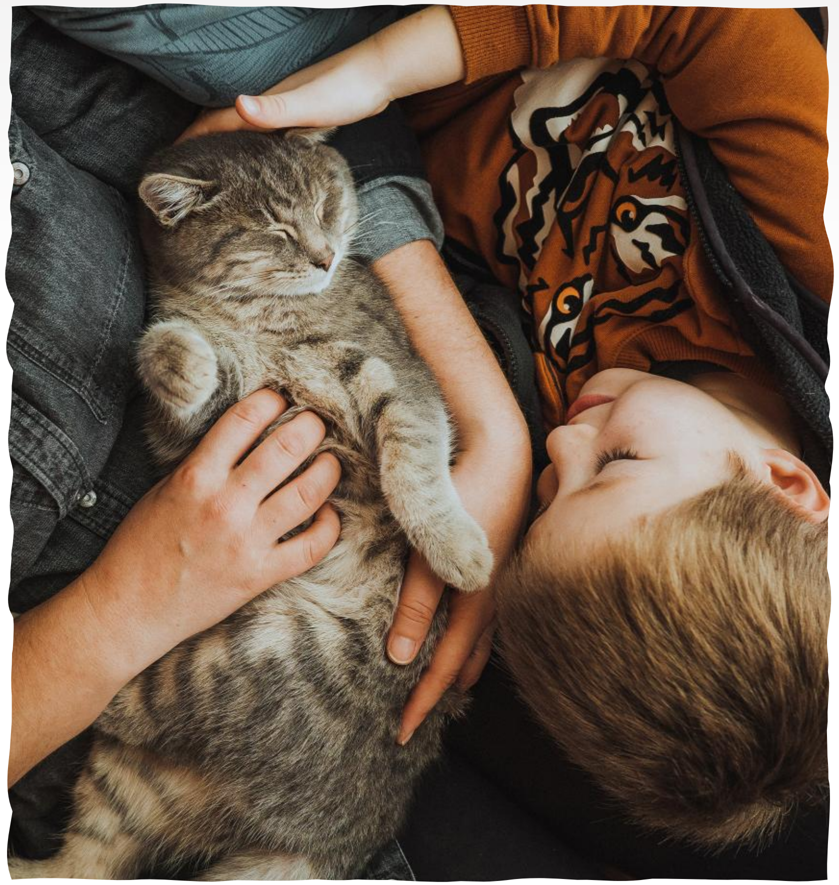

- Supervise interactions
  - Especially with younger children
  - Proper human-cat interaction is a learned skill
- Provide an escape
  - Ability for cat to get away
  - Child-free (or cat-only) areas of the home
- You are the cat interpreter/advocate

(Boone et al., 2025)

UC DAVIS

Animal Welfare Epidemiology Lab · UC DAVIS

Nail Trims etc.

- **Less is more**

- Low-stress handling techniques
  - Passive restraint
  - Towel wraps

- Avoid scruffing & rough or high-pressure handling

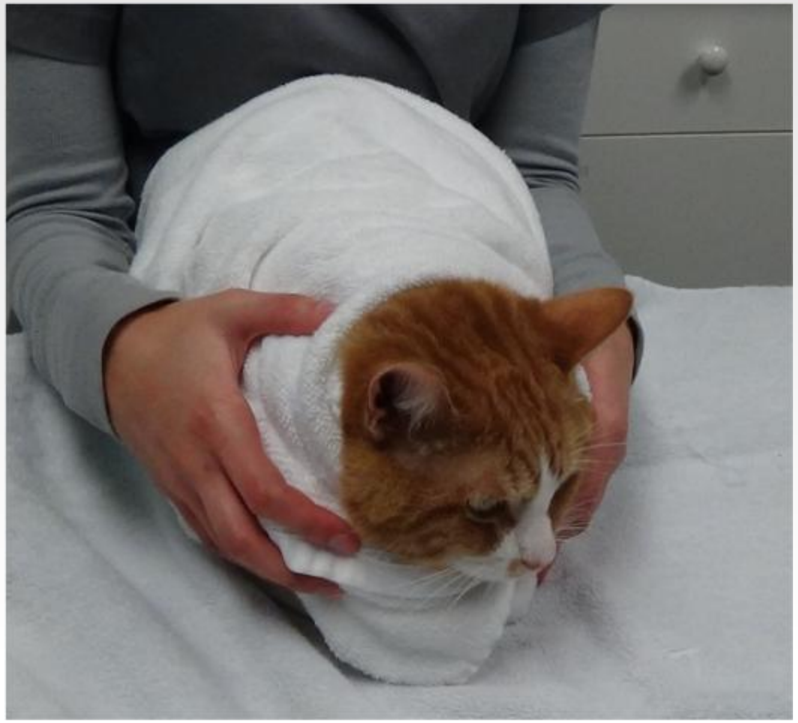

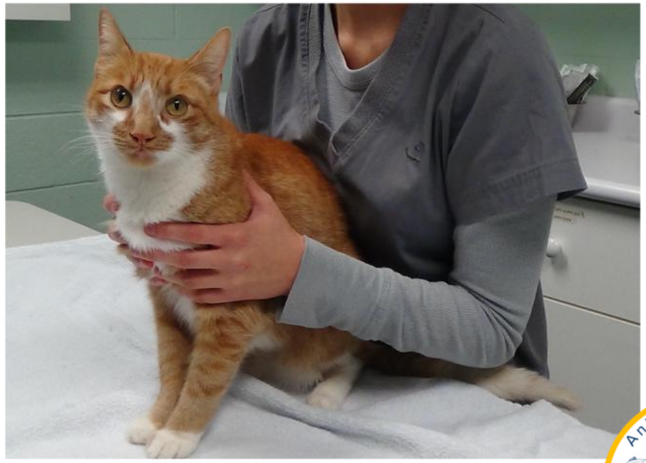

(Boone et al., 2025)

 Rodan et al, 2022

Interactions With Other Pets

- Own resources for each cat
  - Food, water, beds, perching & hiding spots, litter boxes (n+1)
- Ability to get away
  - Escape routes
  - Separate spaces if needed
- Supervise interactions with other pets
  - New: Introductions can take time
  - Younger/healthy: Hiding and inter-animal conflict

(Boone et al., 2025)

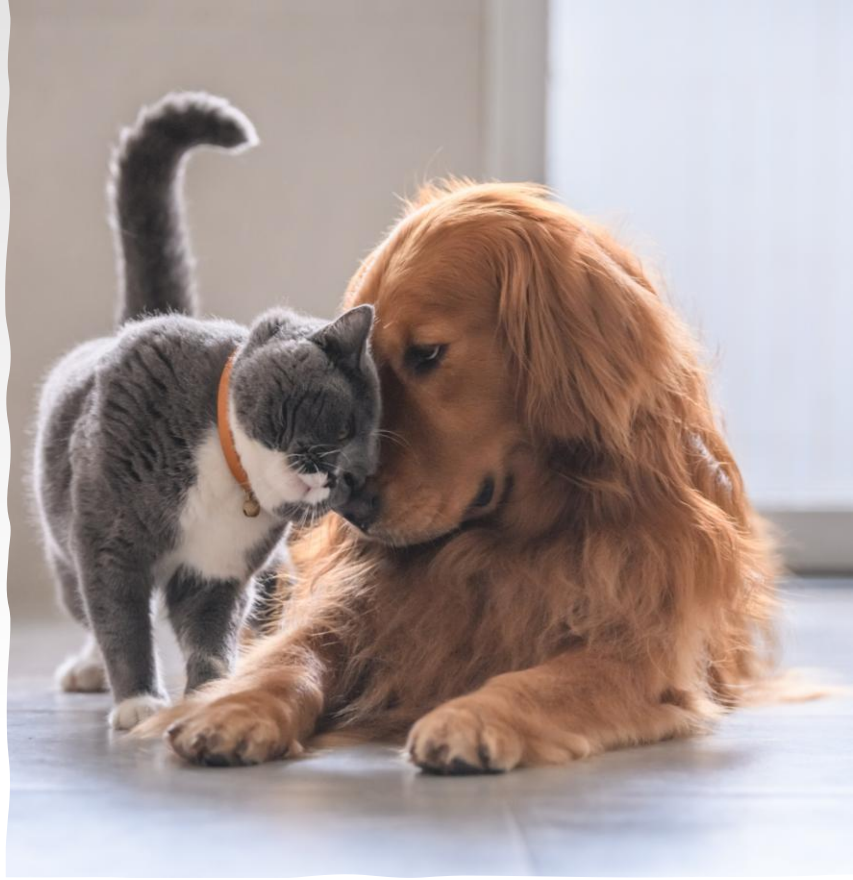

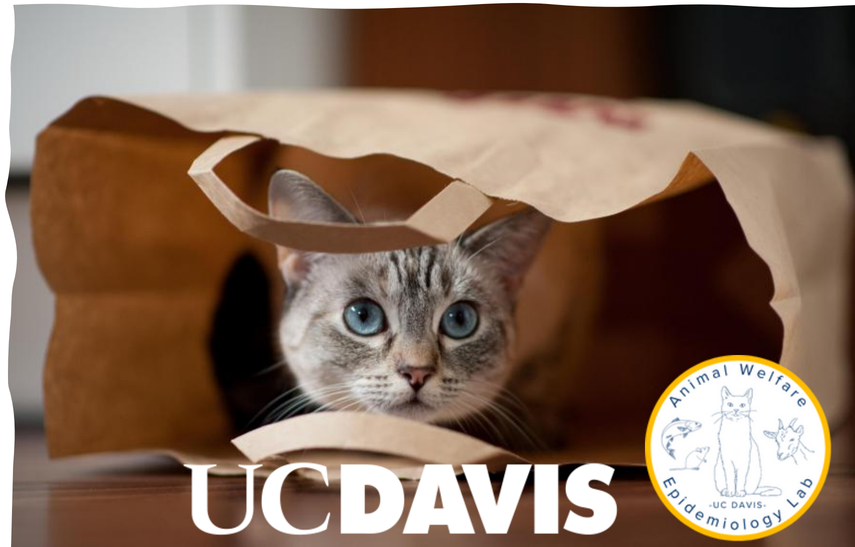

UC DAVIS

Animal Welfare Epidemiology Lab · UC DAVIS

Giving Medications

- Pill training for cats not currently on medications
    - Gravy in syringes
    - Treats in towel wrap

A little now can help a lot later

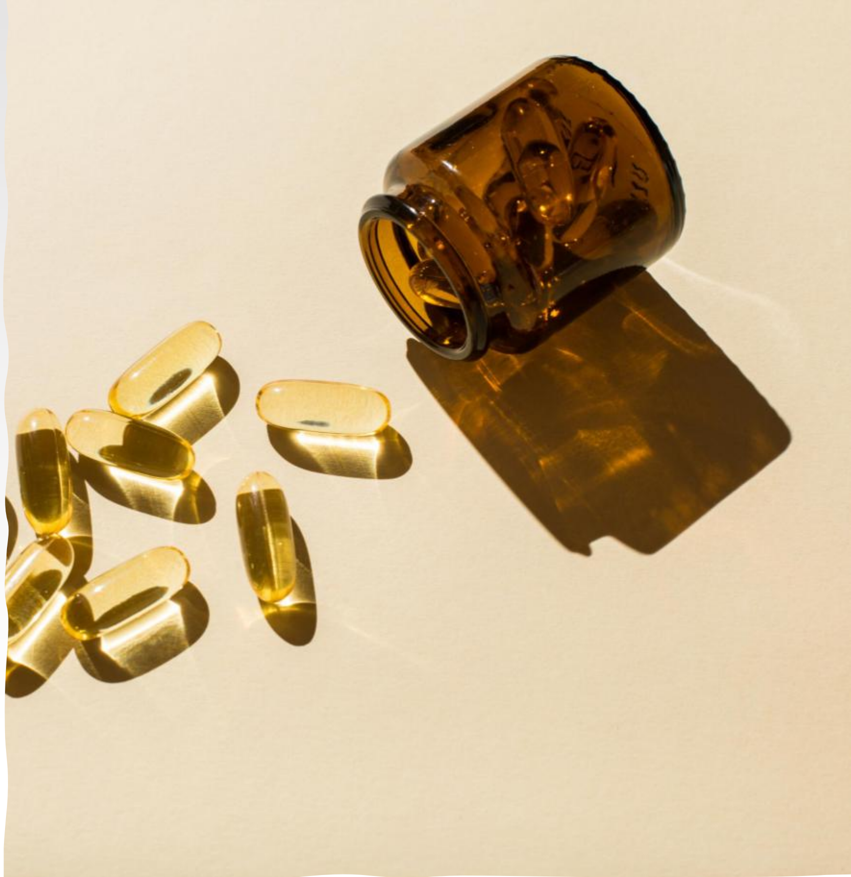

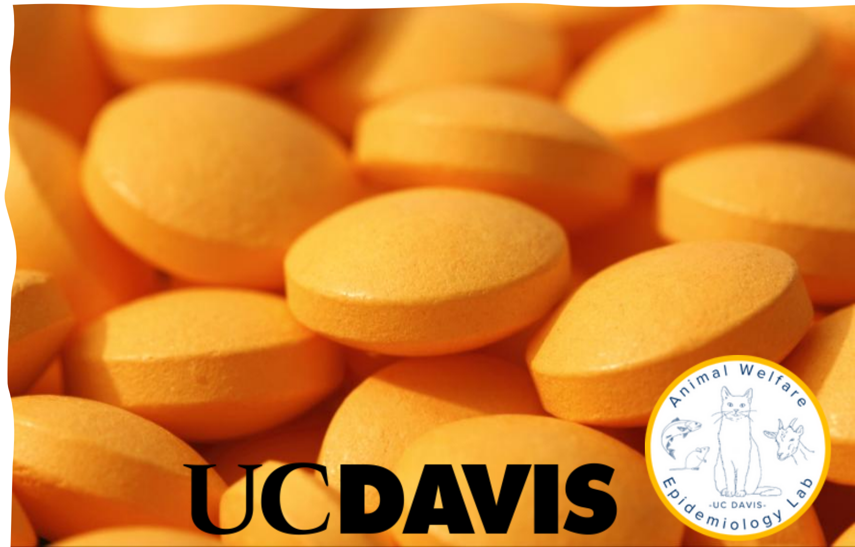

(Boone et al., 2025)

UC DAVIS

Animal Welfare Epidemiology Lab · UC DAVIS

Giving Medications, cont.

Mix with food

(ideal if appropriate)

Towel wrap & leg guard

(positioning)

Treats
(before & after)

Hide pills

(Cat will eat OR not taste when pilled)

Take things slow & be calm

(Boone et al., 2025)

UCDAVIS

Giving Medications, video 1

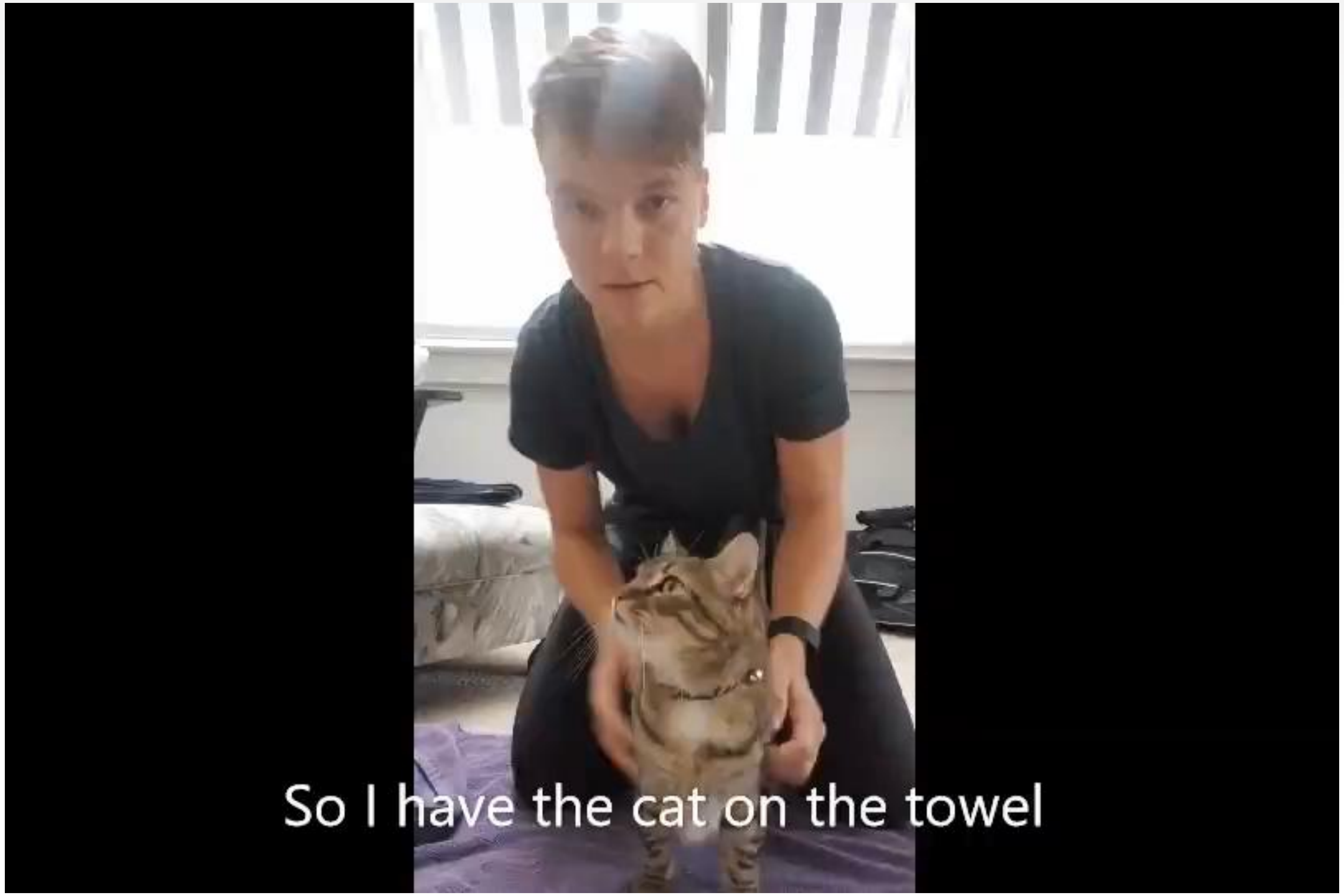

(Boone et al., 2025)

Giving Medications, video 2

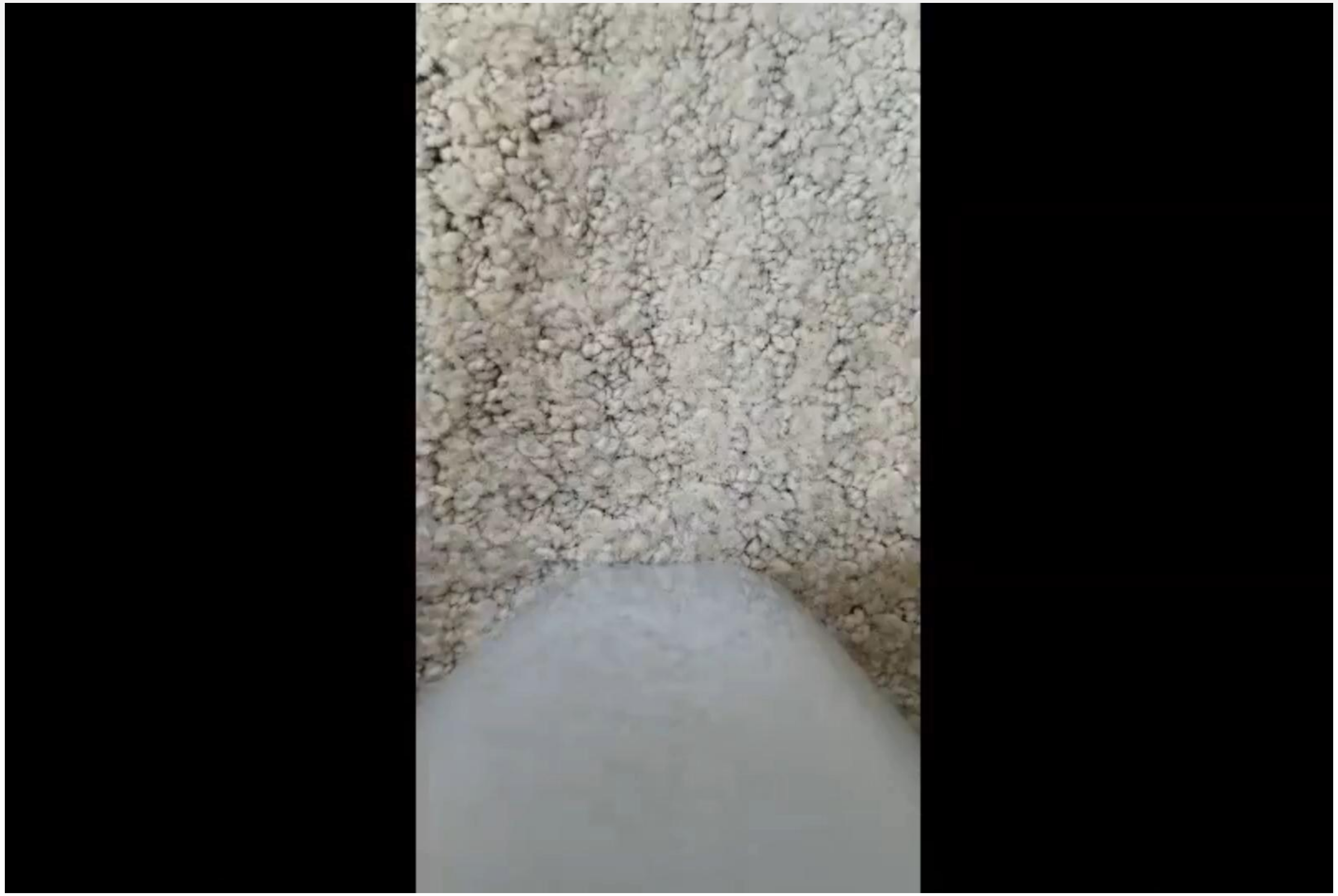

(Boone et al., 2025)

UC DAVIS

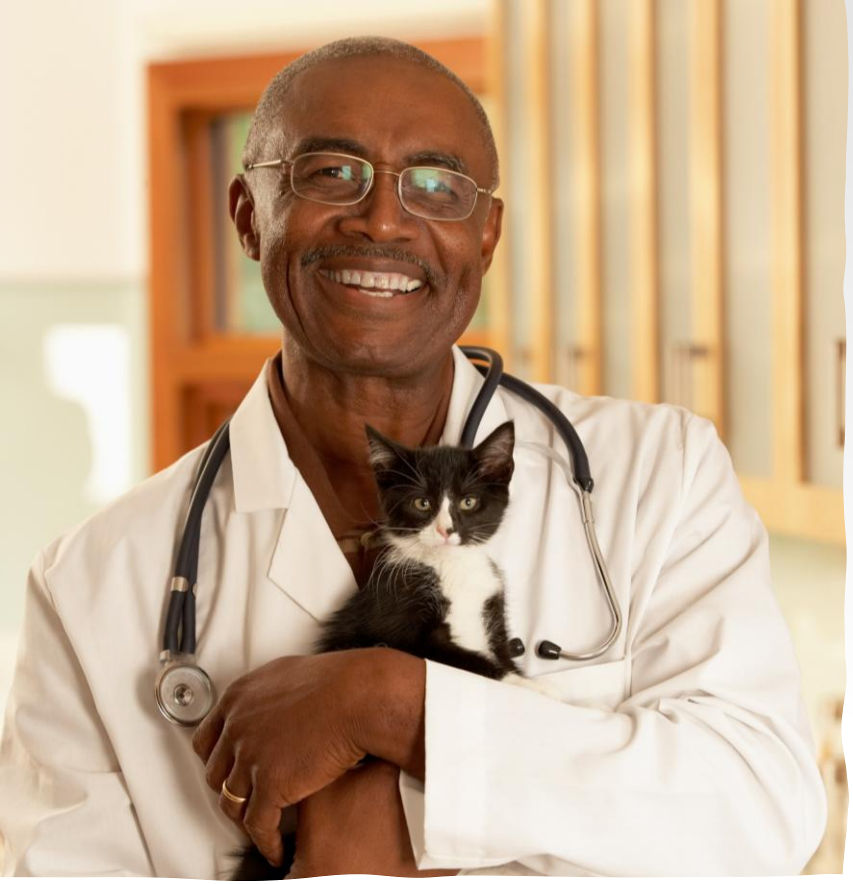

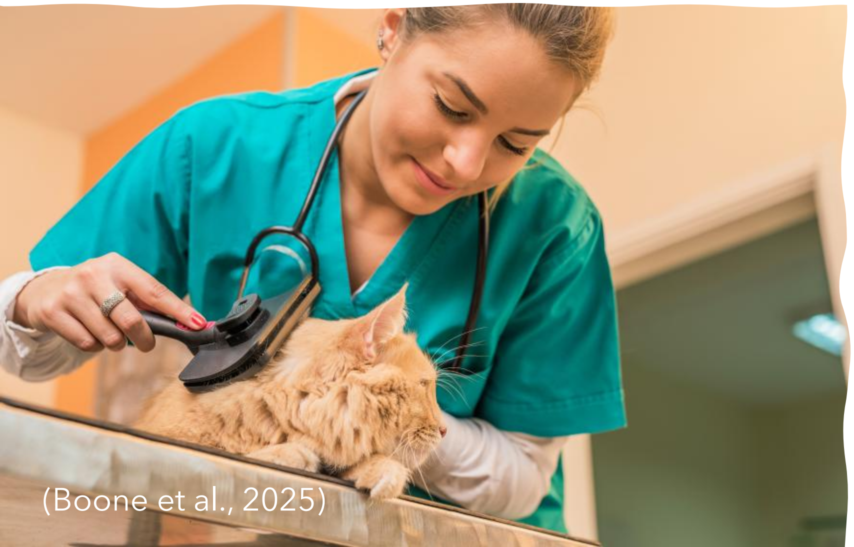

(Boone et al., 2025)

Seeking Veterinary Care

- Cat hasn't been seen by a veterinarian recently [9]
  - 1yr for cats 1-10y
  - 6mos for cats 10-15y
  - 4mos for cats over 15y
- Cat hasn't been seen for mobility challenges
- Sudden, acute changes in mobility/behavior
  - e.g., resents being petted, inappropriate elimination, increased vocalizations, changed interactions with other animals
- Medical causes not ruled out
  - Kidney disease, diabetes, urinary issues

[9] Ray et al., 2021

UC DAVIS

Animal Welfare Epidemiology Lab · UC DAVIS ·

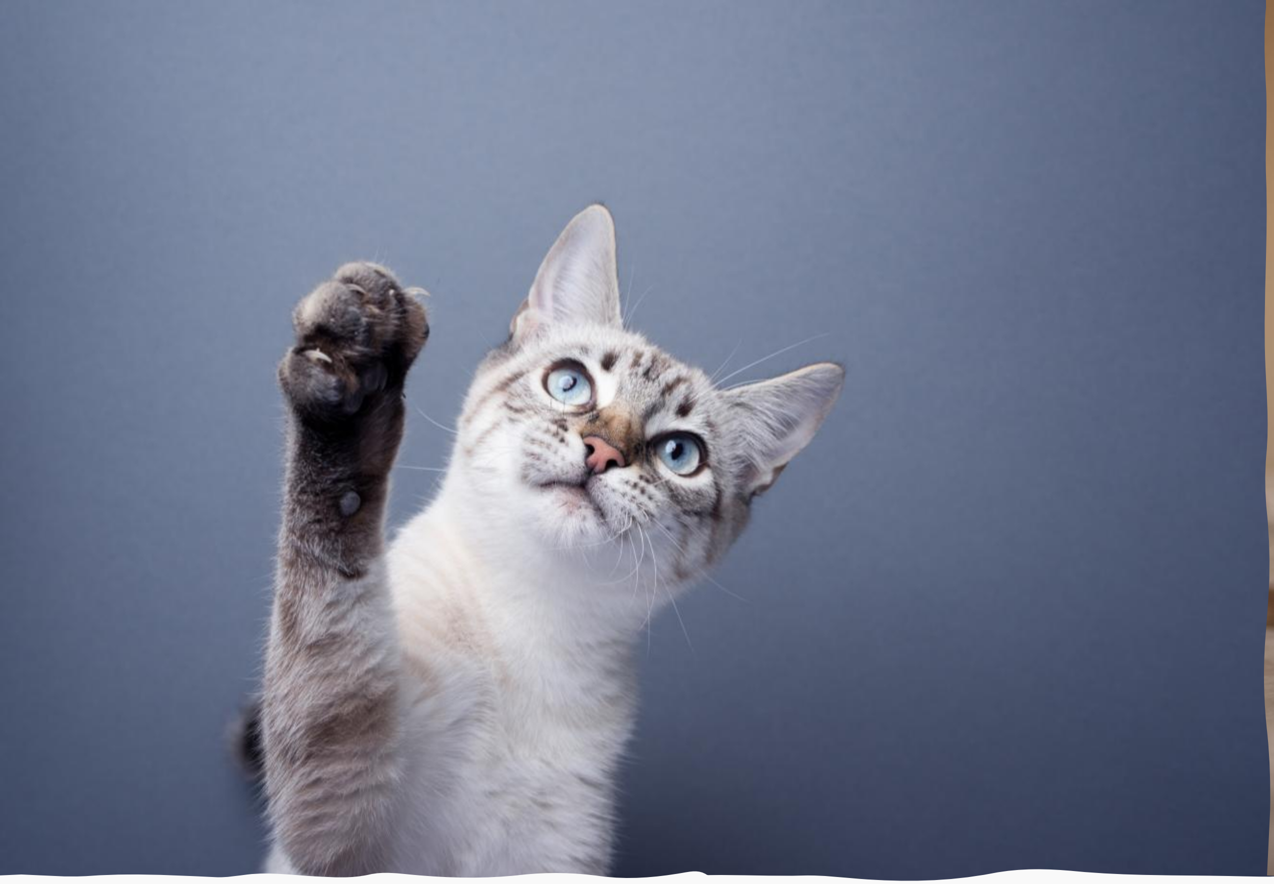

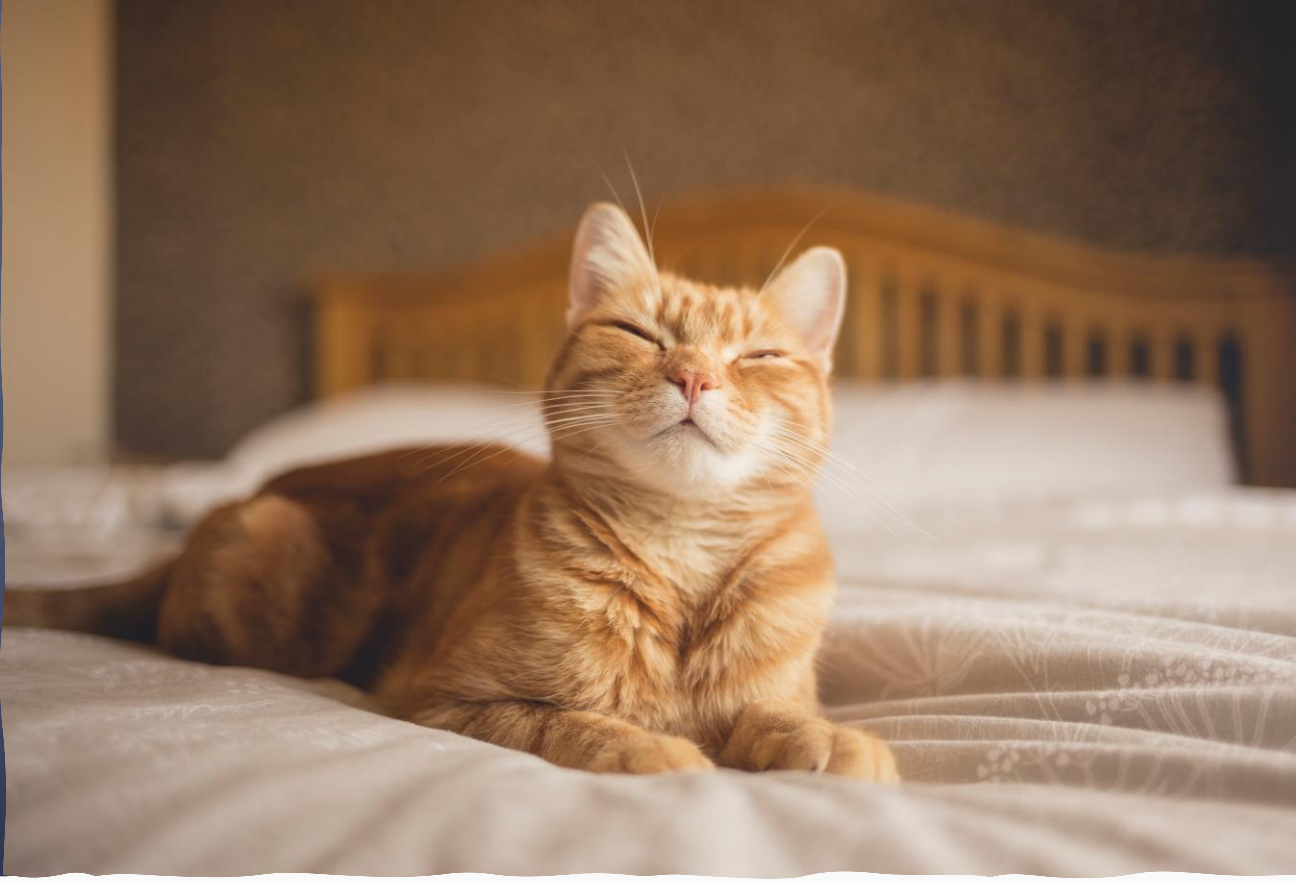

Thank you!

Questions?

(Boone et al., 2025)

References

1. American College of Veterinary Surgeons. Osteoarthritis in cats. Web. Accessed: 04/28/23. (https://www.acvs.org/small-animal/osteoarthritis-in-cats)
2. Williams K & Yuill C. Degenerative joint disease in cats. VCA Animal Hospitals. Web. Accessed: 04/28/23. (https://vcahospitals.com/know-your-pet/degenerative-joint-disease-in-cats)
3. American Animal Hospital Association. Acute vs. chronic pain. 2022. Web. Accessed 04/28/23. (https://www.aaha.org/aaha-guidelines/2022-aaha-pain-management-guidelines-for-dogs-and-cats/developing-a-therapy-plan/acute-vs.-chronic-pain)
4. Lascelles, BDX. Feline degenerative joint disease. Veterinary Surgery. 2010; 39: 2-13. https://doi.org/10.1111/j.1532-950X.2009.00597.x
5. Lascelles BDX, Henry III JB, Brown J, Robertson I, et al. Cross-sectional study of the prevalence of radiographic degenerative joint disease in domesticated cats. Veterinary Surgery. 2010; 39: 535-544. https://doi.org/10.1111/j.1532-950X.2010.00708.x
6. International Cat Care. Arthritis & degenerative joint disease in cats. 2018. Web. Accessed 04/28/23. (https://icatcare.org/advice/arthritis-and-degenerative-joint-disease-in-cats/)
7. Ellis SLH, Thompson H, Guijarro C, Zulch HE. The influence of body region, handler familiarity and order of region handled on the domestic cat's response to being stroked. Applied Animal Behaviour Science. 2015; 173: 60-67. https://doi.org/10.1016/j.applanim.2014.11.002. (https://www.sciencedirect.com/science/article/pii/S0168159114002779)
8. Rodan I, Dowgray N, Carney HC, et al. 2022 AAFP/ISFM Cat friendly veterinary interaction guidelines: Approach and handling techniques. Journal of Feline Medicine and Surgery. 2022; 24(11):1093-1132. https://doi.org/10.1177/1098612X221128760
9. Ray M, Carney HC, Boynton B, et.al. 2021 AAFP feline senior care guidelines. Journal of Feline Medicine and Surgery. 2021; 23: 613-638. DOI: 10.1177/1098612X211021538. (https://journals.sagepub.com/doi/pdf/10.1177/1098612X211021538)

(Boone et al., 2025)

Slide Links

Raised bowl examples:

- https://www.chewy.com/van-ness-ecoware-raised-cat-bowl-cool/dp/756846
- https://www.chewy.com/necoichi-ceramic-elevated-dog-cat/dp/146186

Large tote (litterbox) example:

- https://www.lowes.com/pd/Hefty-13-Gallon-52-Quart-Clear-Underbed-Tote-with-Latching-Lid/1000505517

Cat shampoo, wipes examples:

- https://www.petsmart.com/cat/grooming-supplies/shampoos-and-conditioners/earthbath-hypo-allergenic-cat-shampoo---fragrance-free-50015.html
- https://www.petsmart.com/cat/grooming-supplies/wipes-and-deodorizers/earthbath-hypo-allergenic-cat-wipes---fragrance-free---sensitive-skin---xl-towels---100ct-62176.html

Food puzzles:

- http://foodpuzzlesforcats.com/
- https://icatcare.org/advice/puzzle-feeders/

Jackson Galaxy Video:

- https://www.youtube.com/watch?v=0XPRz8ZUkck&t=658s

(Boone et al., 2025)
